# Genome-wide identification and expression analyses of the homeobox transcription factor family during ovule development in seedless and seeded grapes

**DOI:** 10.1038/s41598-017-12988-y

**Published:** 2017-10-03

**Authors:** Yunduan Li, Yanxun Zhu, Jin Yao, Songlin Zhang, Li Wang, Chunlei Guo, Steve van Nocker, Xiping Wang

**Affiliations:** 1 State Key Laboratory of Crop Stress Biology in Arid Areas, College of Horticulture, Northwest A&F University, Yangling, Shaanxi China; 2 Key Laboratory of Horticultural Plant Biology and Germplasm Innovation in Northwest China, Ministry of Agriculture, Northwest A&F University, Yangling, Shaanxi China; 3 Department of Horticulture, Michigan State University, East Lansing, Michigan USA

## Abstract

Seedless grapes are of considerable importance for the raisin and table grape industries. Previous transcriptome analyses of seed development in grape revealed that genes encoding homeobox transcription factors were differentially regulated in seedless compared with seeded grape during seed development. In the present study, we identified a total of 73 homeobox-like genes in the grapevine genome and analyzed the genomic content and expression profiles of these genes. Based on domain architecture and phylogenetic analyses grape homeobox genes can be classified into eleven subfamilies. An analysis of the exon-intron structures and conserved motifs provided further insight into the evolutionary relationships between these genes. Evaluation of synteny indicated that segmental and tandem duplications have contributed greatly to the expansion of the grape homeobox gene superfamily. Synteny analysis between the grape and Arabidopsis genomes provided a potential functional relevance for these genes. The tissue-specific expression patterns of homeobox genes suggested roles in both vegetative and reproductive tissues. Expression profiling of these genes during the course of ovule development in seeded and seedless cultivars suggested a potential role in ovule abortion associated with seedlessness. This study will facilitate the functional analysis of these genes and provide new resources for molecular breeding of seedless grapes.

## Introduction

Homeobox (HB) transcription factors often act as master regulators of organ identity and are encoded by a large and conserved gene family. These were originally characterized as regulators of morphogenesis in the fruit fly, *Drosophila melanogaster* ^1^. In plants, the founding member of the HB gene family is KNOTTED1, which has a role in meristem function in maize^2^. Subsequently, numerous HB-encoding genes have been identified from a range of eukaryotes^3^, including Arabidopsis^4^, rice^5^, barley^6^, and humans^7^.

The homeodomain (HD) is a conserved, ~60 amino acids long DNA-binding domain, and is encoded by a ~180-nucleotide sequence referred to as the HB^8^. The characteristic three-dimensional structure of the HD comprises three alpha-helices, with the first and second helices forming a loop structure^9^. The second and third helices form a helix-turn-helix motif, which can interact with specific DNA sequences, allowing for regulation of expression of target genes^10,11^. Based on the conserved sequence of the HD along with other characteristic domains and motifs, HB genes are classified into 14 families^12^, including HD-ZIP I-IV, KNOX, WOX, PHD, NDX, BEL, PLINC, DDT, LD, SAWADEE and PINTOX. Each HB gene family is named according to unique typical domains and motifs outside of the HD, features that may enable functional differences of each subfamily.

HB genes are involved in many aspects of plant growth and development, and participate in various hormone response pathways^13–15^. For example, members of HD-ZIP, the largest HB protein family, play roles in epidermal cell differentiation^16^, floral organogenesis, fruit ripening^15^, embryonic shoot meristem formation, embryo patterning and vascular development^17^. Interestingly, this family is apparently specific to plants^18^. Members of the KNOX family are known to be required for nuclear localization and homo-dimerization, and suppress target gene expression^19,20^. Additionally, studies have shown that KNOX genes are involved in cell differentiation and shoot apical meristem maintenance^21,22^. BEL family members play a critical regulatory role in ovule development^23^, phyllotactic patterning, and stem cell maintenance and fate^24^.

In grape (*Vitis vinifera*), seedlessness is of particular importance for both fresh and dry fruit. Seedlessness in grape results from two distinct mechanisms, parthenocarpy and stenospermocarpy. The formation of stenospermocarpic fruit is associated with progressive ovule abortion following full bloom. Previous transcriptome analyses of seed development in grape hybrids revealed numerous genes, including HB genes, with expression associated with seedlessness^25^. However, there is little information about the number, organization and regulation of HB genes in grapevine. In this study, we evaluated the genomic content and expression profiles of HB genes in grapevine. We identified 73 grape HB genes, and classified these into 11 families based on phylogenetic analysis with Arabidopsis. Tissue-specific expression analysis in vegetative and reproductive organs suggests that many of the HB genes are developmentally regulated. Furthermore, comparison of ovule-specific expression during the course of ovule development in seeded and seedless grapes suggests that at least a subset of HB genes might be important for ovule development. Taken together, these results will provide a few candidate genes involved in ovule development for future targeted functional characterization, which may be useful in seedlessness-related molecular breeding programs.

## Results

### Genome-wide identification of HB genes in grape

To identify potential HB genes in the grapevine genome, we first obtained an HMM algorithm (HMMER) for the conserved HD (PFAM PF00046)^26^, and then used this in conjunction with PSI-BLAST to search a draft grape genome sequence (Grape Genome Website: http://www.genoscope.cns.fr)^27^. Subsequently, the structural integrity of conserved domains was evaluated, and redundant sequences were eliminated. Using this approach, we identified a total of 73 HB genes. These genes were designated as *Vitis vinifera* HB (*VvHB*) 1–73 based on their chromosomal positions (Table 1). The gene locus identifiers, gene accession numbers obtained from NCBI, position and length of the coding sequences, and length of the open reading frames are shown in Table 1.

**Table 1:** Characteristics of grape HB genes.

Gene locus ID	Gene ID	Accession no.	Chromosome	Start	End	CDS (bp)	ORF (aa)
GSVIVT01011754001	*VvHB01*	CBI26907	Chr1	4488301	4491290	678	225
GSVIVT01011738001	*VvHB02*	CBI26893	Chr1	4685930	4687708	1014	337
GSVIVT01013790001	*VvHB03*	CBI28570	Chr1	7552215	7562392	957	318
GSVIVT01020078001	*VvHB04*	CBI32024	Chr1	10605111	10607758	966	321
GSVIVT01020033001	*VvHB05*	CBI31991	Chr1	11304894	11307266	768	255
GSVIVT01019399001	*VvHB06*	CBI34373	Chr2	330476	334581	1338	445
GSVIVT01019655001	*VvHB07*	CBI34588	Chr2	2249075	2251461	669	222
GSVIVT01019880001	*VvHB08*	CBI34768	Chr2	4164201	4172140	876	291
GSVIVT01013073001	*VvHB09*	CBI33830	Chr2	8739164	8746005	2397	798
GSVIVT01024224001	*VvHB10*	CBI38029	Chr3	40975	48142	1752	583
GSVIVT01037894001	*VvHB11*	CBI22504	Chr3	6571801	6592129	2934	977
GSVIVT01035361001	*VvHB12*	CBI20629	Chr4	746923	749384	1035	344
GSVIVT01035612001	*VvHB13*	CBI20838	Chr4	2700607	2710044	2520	839
GSVIVT01035827001	*VvHB14*	CBI21017	Chr4	4701759	4710455	1029	342
GSVIVT01035921001	*VvHB15*	CBI21101	Chr4	5879265	5888462	660	219
GSVIVT01035238001	*VvHB16*	CBI27350	Chr4	10997910	11025564	2145	714
GSVIVT01019043001	*VvHB17*	CBI17588	Chr4	17429918	17436016	2037	678
GSVIVT01019012001	*VvHB18*	CBI17561	Chr4	17798124	17799885	636	211
GSVIVT01018787001	*VvHB19*	CBI17382	Chr4	19837737	19839174	843	280
GSVIVT01026638001	*VvHB20*	CBI37641	Chr4	20843708	20847365	720	239
GSVIVT01026636001	*VvHB21*	CBI37640	Chr4	20861325	20865130	711	236
GSVIVT01025220001	*VvHB22*	CBI16340	Chr6	3098092	3107496	2241	746
GSVIVT01025193001	*VvHB23*	CBI16318	Chr6	3507793	3517339	2535	844
GSVIVT01025075001	*VvHB24*	CBI16222	Chr6	4759942	4760340	399	132
GSVIVT01037575001	*VvHB25*	CBI24428	Chr6	10835007	10839521	1722	573
GSVIVT01037260001	*VvHB26*	CBI24231	Chr6	16796502	16815796	1530	509
GSVIVT01003431001	*VvHB27*	CBI33148	Chr7	14952294	14954822	843	280
GSVIVT01029942001	*VvHB28*	CBI31211	Chr8	1900037	1901212	711	236
GSVIVT01011146001	*VvHB29*	CBI22785	Chr8	7222570	7227039	1470	489
GSVIVT01034073001	*VvHB30*	CBI30476	Chr8	15380687	15387772	1686	561
GSVIVT01033744001	*VvHB31*	CBI30218	Chr8	18187600	18188969	663	220
GSVIVT01033481001	*VvHB32*	CBI30005	Chr8	20357119	20358771	753	250
GSVIVT01017010001	*VvHB33*	CBI36079	Chr9	3414695	3425101	2508	835
GSVIVT01017073001	*VvHB34*	CBI36129	Chr9	3967669	3972062	2253	750
GSVIVT01012643001	*VvHB35*	CBI23181	Chr10	300629	304962	2181	726
GSVIVT01021113001	*VvHB36*	CBI30611	Chr10	1562230	1582041	5049	1682
GSVIVT01021144001	*VvHB37*	CBI30635	Chr10	1805314	1808227	1395	464
GSVIVT01021625001	*VvHB38*	CBI30999	Chr10	8333352	8341424	2538	845
GSVIVT01004811001	*VvHB39*	CBI32996	Chr10	92751	99853	750	249
GSVIVT01012897001	*VvHB40*	CBI25599	Chr11	6584507	6590529	1242	413
GSVIVT01010860001	*VvHB41*	CBI40100	Chr11	16762386	16772741	2184	727
GSVIVT01020605001	*VvHB42*	CBI21902	Chr12	3840176	3862721	5613	1870
GSVIVT01030488001	*VvHB43*	CBI28850	Chr12	6115199	6119855	747	248
GSVIVT01030605001	*VvHB44*	CBI28946	Chr12	7101002	7106874	2274	757
GSVIVT01016458001	*VvHB45*	CBI31605	Chr13	3701110	3709563	2808	935
GSVIVT01016360001	*VvHB46*	CBI31530	Chr13	4546379	4547834	522	173
GSVIVT01016272001	*VvHB47*	CBI31458	Chr13	5640475	5655021	2523	840
GSVIVT01001366001	*VvHB48*	CBI32169	Chr13	24027379	24029185	1074	357
GSVIVT01031241001	*VvHB49*	CBI39595	Chr14	928360	935678	933	310
GSVIVT01032491001	*VvHB50*	CBI34940	Chr14	27846210	27848937	774	257
GSVIVT01011377001	*VvHB51*	CBI22185	Chr14	29151428	29152873	852	283
GSVIVT01018247001	*VvHB52*	CBI16641	Chr15	12566181	12593884	879	292
GSVIVT01027508001	*VvHB53*	CBI38766	Chr15	16132617	16138896	2316	771
GSVIVT01027407001	*VvHB54*	CBI38687	Chr15	16989617	16991196	582	193
GSVIVT01010600001	*VvHB55*	CBI31820	Chr16	16145022	16162952	2658	885
GSVIVT01038619001	*VvHB56*	CBI23065	Chr16	21439501	21441052	678	225
GSVIVT01008424001	*VvHB57*	CBI15564	Chr17	2230450	2232935	1044	347
GSVIVT01008065001	*VvHB58*	CBI15277	Chr17	6134096	6136429	792	263
GSVIVT01007715001	*VvHB59*	CBI15003	Chr17	9989635	10000315	960	319
GSVIVT01029396001	*VvHB60*	CBI35303	Chr17	16227031	16230509	2025	674
GSVIVT01013388001	*VvHB61*	CBI40730	Chr18	780748	784482	840	279
GSVIVT01009083001	*VvHB62*	CBI19202	Chr18	4806872	4808947	939	312
GSVIVT01009273001	*VvHB63*	CBI19343	Chr18	6870320	6876771	1026	341
GSVIVT01009274001	*VvHB64*	CBI19344	Chr18	6881993	6883411	897	298
GSVIVT01009453001	*VvHB65*	CBI19488	Chr18	8514131	8515605	636	211
GSVIVT01009633001	*VvHB66*	CBI19630	Chr18	10068356	10074521	1050	349
GSVIVT01009779001	*VvHB67*	CBI19758	Chr18	11196526	11198339	1413	470
GSVIVT01009781001	*VvHB68*	CBI19760	Chr18	11201410	11205044	1587	528
GSVIVT01014258001	*VvHB69*	CBI20236	Chr19	2065687	2074580	1260	419
GSVIVT01014276001	*VvHB70*	CBI20251	Chr19	2259485	2272052	1350	449
GSVIVT01036776001	*VvHB71*	CBI24184	Chr19	22688699	22717134	3567	1188
GSVIVT01005821001	*VvHB72*	CBI29502	ChrUn	22117052	22119297	894	297
GSVIVT01002447001	*VvHB73*	CBI41059	ChrUn	34773498	34774970	801	266

Abbreviations: Chr, chromosome; CDS, coding sequence; ORF, open reading frame; Un, unknown chromosome.

The result of multiple sequence alignments of the 73 grape HD protein sequences revealed five highly conserved amino acids within the HD (Leu-16, Trp-48, Phe-49, Asn-51, and Arg-53), which may be necessary for the *VvHB* genes (Supplementary Fig. S1 **)**.

### Phylogenetic analysis of grape and Arabidopsis HB genes

**Figure 1:**
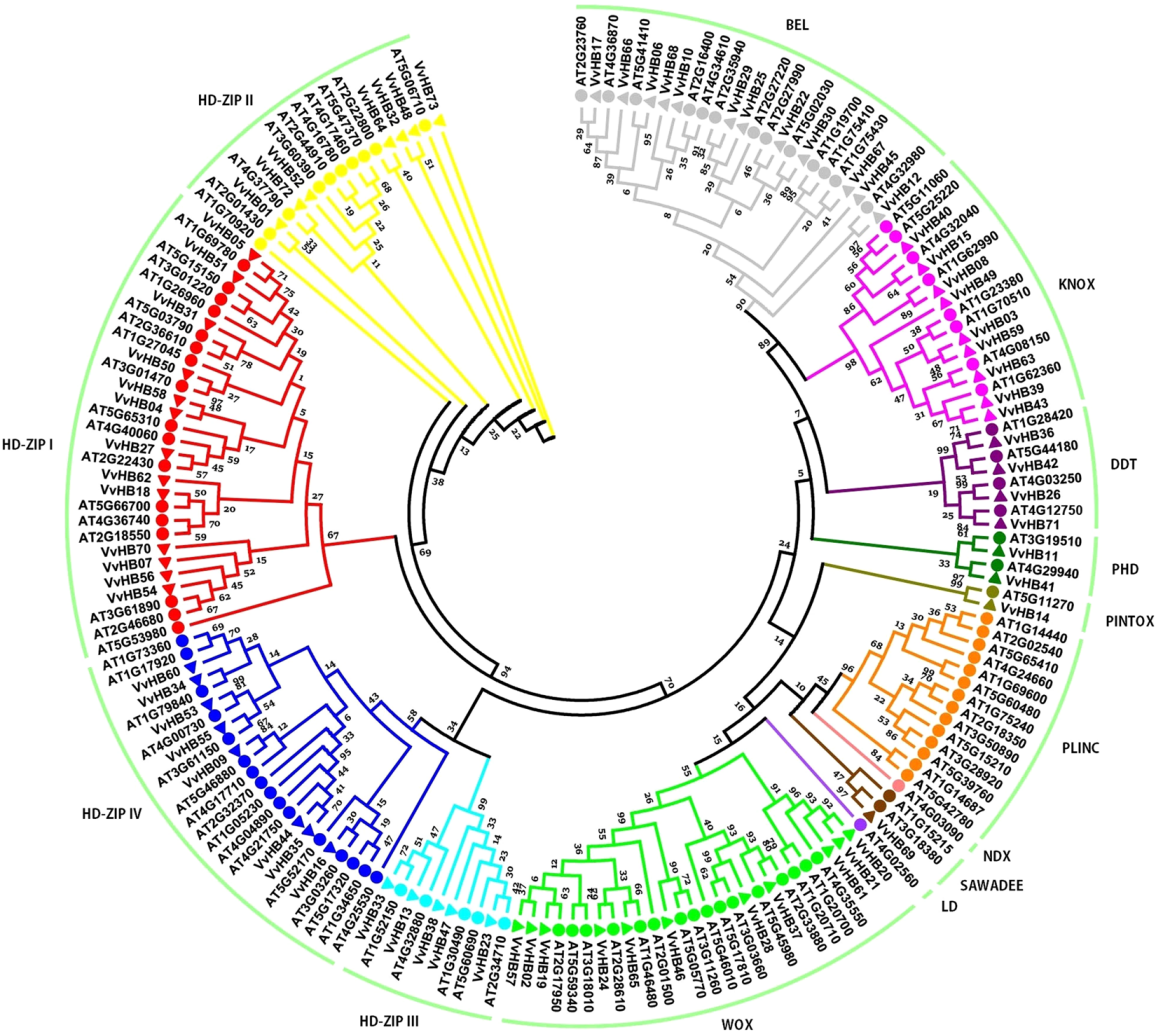
Phylogenetic analysis of HB proteins from grape and Arabidopsis. The phylogenetic tree was constructed with alignments of HD sequences from each gene using MEGA5.0 software. Circles represent Arabidopsis proteins and triangles represent grape proteins. Different colors of branch lines of subtrees indicate different subfamilies. Numbers at the nodes indicate bootstrap values.

Multiple sequence alignment (Supplementary Fig. S1) of grape HD proteins showed that the most conserved structural feature is the HD, which consists of three alpha-helices. To gain a better understanding about the evolutionary relationships of HB genes among plant species, a total of 183 HB genes, including the 73 genes from grape identified in this study and 110 genes from Arabidopsis, were used to construct a phylogenetic tree based on the HD. Fourteen main clades (HD-ZIP I, HD-ZIP II, HD-ZIP III, HD-ZIP IV, KNOX, WOX, PHD, NDX, BEL, PLINC, DDT, LD, SAWADEE and PINTOX) were identified. Most of the main clades contained representatives from both grape and Arabidopsis, suggesting that a common ancestor of each subfamily must have existed before the divergence of these plant lineages (Fig. 1 and Supplementary Table S1 **)**. Based on the domain composition and evolutionary relationships of their encoded proteins, the grape HB genes were divided into 11 subfamilies. Interestingly, in grape, no members belonged to the three subgroups PLINC, NDX or LD, which existed in the Arabidopsis genome, suggesting that gene deletions may have occurred during evolution. This was especially striking for the PLINC clade, which contained 14 genes in Arabidopsis but none in grape. The numbers of grape HB genes in most other groups (HD-ZIP I, HD-ZIP II, HD-ZIPIII, KNOX, BEL, DDT, PHD, PINTOX and SAWADEE) were similar to Arabidopsis.

### Sequence and structure analyses of grape HB genes

**Figure 2:**
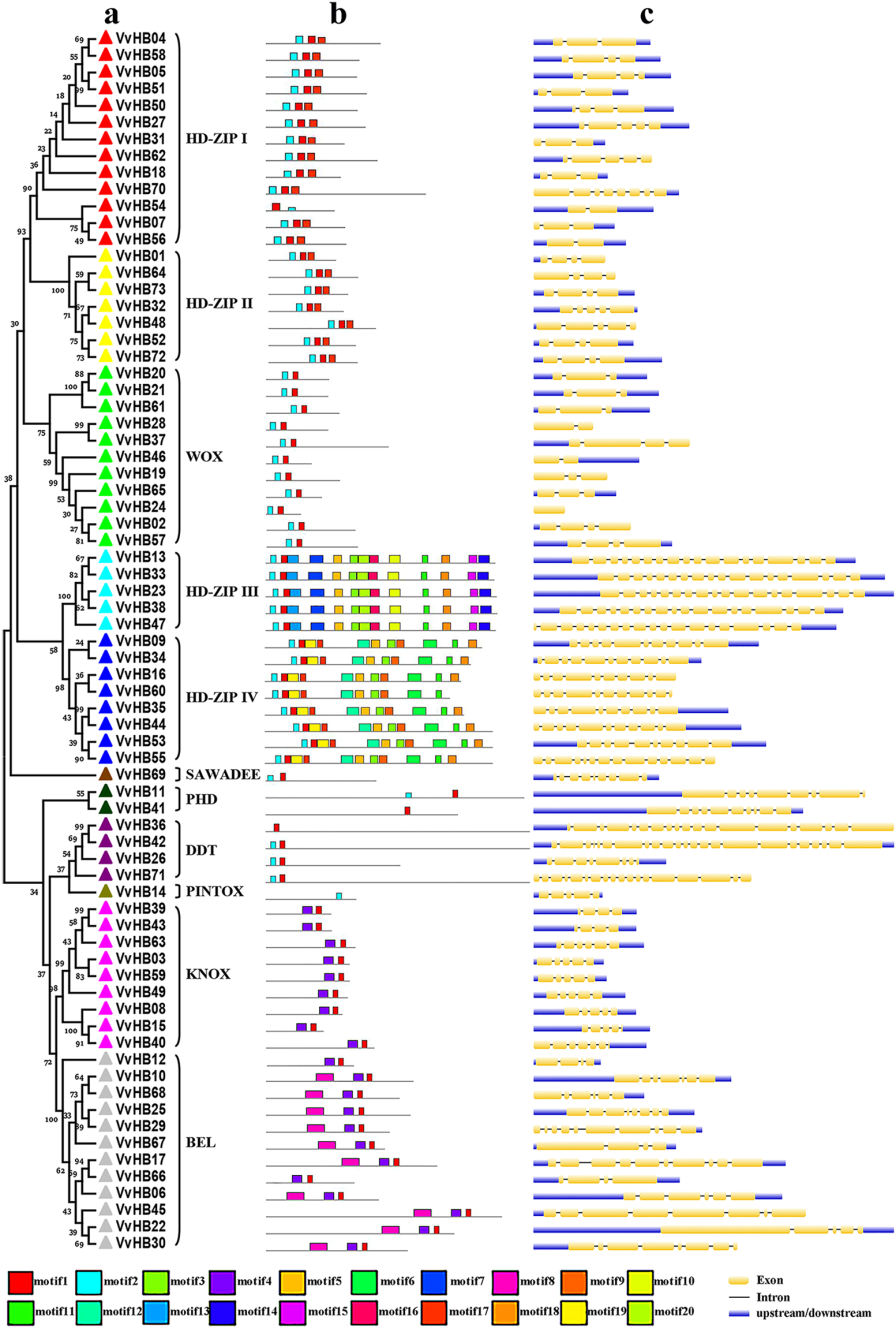
The structure analysis of grape HB genes. (**a)** Phylogenetic analysis and classification of grape genes. The phylogenetic tree was constructed with alignments of HD sequences from each gene using MEGA 5.0 software. Subtree branch lines are colored indicating different subfamilies. Numbers near the tree branches indicate bootstrap values. (**b**) Motif analysis of grape proteins. Motifs, numbered 1-20, were identified using MEME 4.11.2 software and are distinguished by color. Peptide sequence of each motif is provided in Supplementary Table S2. (**c**) Exon-intron structures of grape HB genes. Exons are marked as yellow boxes, and introns are represented by black lines connecting two exons. Upstream/downstream sequences are shown as blue boxes. Only exons and upstream/downstream sequences are drawn to scale.

We used the HD sequences from the 73 grape HB proteins identified here to build a phylogenetic tree, using the neighbor-joining method (Fig. 2a). HD proteins from the same subfamilies within grape clustered together, and the topology was similar to that constructed with HDs from Arabidopsis and grape. In order to further explore the phylogenetic relationship and classification of *VvHB* genes and the evolutionary relationship between *VvHB* genes and *ATHB* genes, we determined the distribution of their conserved motifs using MEME software (http://meme-suite.org/tools/meme) (Fig. 2b and Supplementary Fig. S2). In the current study, a total of 20 conserved motifs were identified within VvHB proteins and ATHB proteins. Nearly all of the HB proteins contain a highly conserved motif 1, corresponding to the alpha-helix III of the HD, which forms a helix-turn-helix structure with the alpha-helix II, and this structure can recognize and bind to specific DNA sequences to regulate the expression of target genes^10,11^. The motif 2 existing in most subfamilies corresponds to the HD alpha-helix I, which forms a loop structure with alpha-helix II. BEL subgroup members exhibited a motif 8, corresponding to POX domain, and this motif was present only in the BEL subgroup (Fig. 2b and Supplementary Table S2). Members of the HD-ZIP III and HD-ZIP IV subgroups exhibited 13 and 11 motifs, respectively. The two subgroups had four common motifs (motif 3, motif 5 and motif 11 correspond to START domain, motif 18 corresponds to HD-SAD domain). Additionally, there are two characteristic motifs in HD-ZIP III subgroup corresponding to MEKHLA structure. Overall, VvHB proteins within a given subgroup showed a similar distribution of conserved motifs, and this distribution was similar to that in Arabidopsis. For instance, all VvHB proteins from the HD-ZIP III group contained the same 13 conserved motifs (1, 2, 3, 5, 7, 10, 11, 13, 14, 15, 16, 18 and 20), which were also present in members of the ATHB subgroup (Supplementary Fig. S2 and Supplementary Table S2).

Divergence of exon/intron structure is known to play a vital role in the evolution of gene families. Accordingly, we analyzed the exon/intron structures of *VvHB* genes to gain insight into evolution of the *VvHB* superfamily^28^. As shown in Fig. 2c, the number of exons ranged dramatically, from 1 to 23. Most of *VvHB* genes showed three to five exons (10 with three exons, 14 with four exons and 12 with five exons). However, *VvHB24* has only one exon, *VvHB55* has 14 exons, and *VvHB42* has 23 exons, suggesting that during the evolution of the *VvHB* gene family, both exon loss and gain have occurred. This phenomenon may result in functional diversity of closely related genes. In addition, there was an obvious correlation between the phylogeny and exon/intron structure. Within the same family, *VvHB* genes tended to exhibit similar numbers of exons. For example, the number of exons in HD-ZIP III group was relatively large, ranging from eighteen to nineteen, while genes in WOX family exhibited a relatively small number, ranging from one to four. This similarity of exon pattern may be ascribed to a large number of gene duplications. Particularly, in the DDT group, three of the four members have a larger number: *VvHB36* has 19 exons, *VvHB71* has 18 exons and *VvHB42* has 23 exons. In contrast, *VvHB26* has only nine exons, potentially a result of special variations in evolution.

### Domain architecture analysis of grape HB proteins

**Figure 3:**
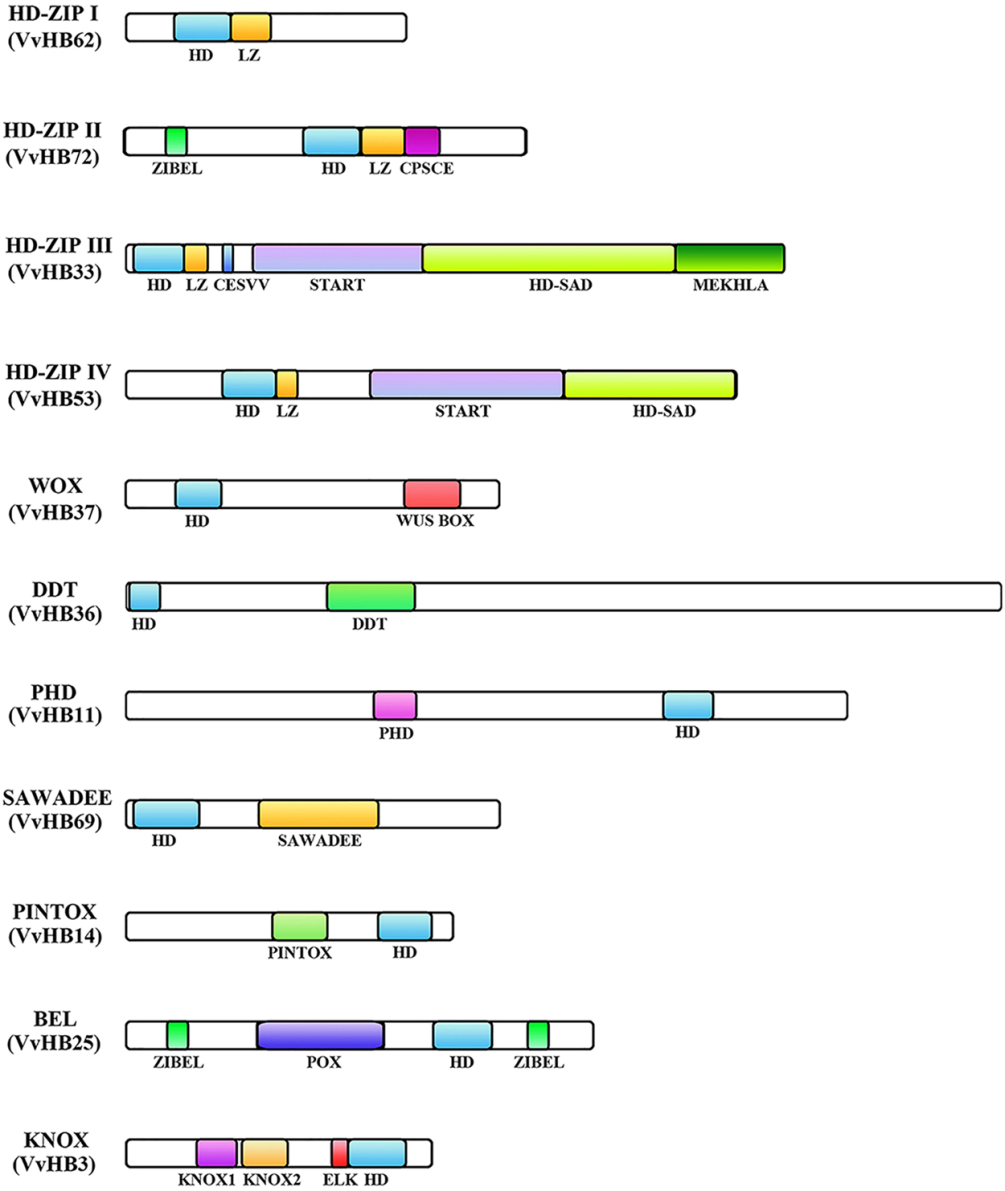
Diagrammatic representation of the characteristic domains of representatives of the 11 subfamilies of grape HB proteins. The following domains and motifs are indicated: HD (homeodomain), LZ (leucine-zipper), ZIBEL motif, CPSCE motif, CESVV motif, START domain, HD-SAD domain (HD-START associated domain), MEKHLA domain, WUS Box, DDT domain, DDT domain, PHD domain, SAWADEE domain, PINTOX domain, POX domain, KNOX domain (1 and 2), ELK motif. Characteristic motifs and domains were identified using SMART and MEME software.

Plant HB proteins can be divided into 14 distinct families with robust (generally 70% or more) bootstrap support^12^. We found that the VvHB proteins represented 11 of these families. A schematic diagram of the domain architecture of representatives of each of these families was shown in Fig. 3. The category and distribution of these typical structural domains identified in grape were similar to previous studies in other plants such as Arabidopsis, poplar, maize and rice^12^. This result showed that these structural domains have been highly conserved in plant evolution.

Similar to other plants, the grape HD-ZIP superfamily contains most of the members (33 in our alignment). The HD-ZIP superfamily comprises four individual families, HD-ZIP I, HD-ZIP II, HD-ZIP III, and HD-ZIP IV. All of these are characterized by the presence of a leucine zipper motif, which may mediate protein-protein interactions. The CPSCE motif in HD-ZIP II proteins is found immediately adjacent to the leucine zipper, and plays a role as a redox sensor^29^. HD-ZIP II proteins also include a ZIBEL motif, which has been shown to be important for interaction between HD-Zip II proteins and BEL HD proteins^12^. The HD-ZIP III and HD-ZIP IV classes both contain a START (STeroidogenic Acute Regulatory protein–related lipid Transfer)^30^ domain and a HD-SAD (START associated conserved domain)^31,32^. HD-ZIP III proteins are distinguished from HD-ZIP IV by virtue of a conserved MEKHLA domain at the carboxyl terminus.

Grape contains 11 members of the WOX class. WOX proteins have an HD of 68 amino acids, which show one or two extra residues between alpha-helices one and two, and four to five extra residues between alpha-helices two and three (Supplementary Fig. S1). WOX class proteins also contain a WUS-box motif at a position carboxyl-terminal to the HD, as well as an acidic amino acid stretch between the HD and WUS-box^33^.

Four HB proteins belong to the DDT class, which contain a DDT domain located carboxyl-terminal to the HD^34^. This domain can be divided into eight additional conserved motifs, D-TOX A to H^35^.

HB proteins containing a PHD domain have been most commonly characterized as pathogenesis-related. Examples include the Pathogenesis-Related HB gene A (PRHA)^36^ and HAT3.1 genes^37^ from Arabidopsis. The PHD domain is several hundred amino acids long and is located amino-terminal to the HD. There are two HB genes encoding HB-PHD proteins in both grape and Arabidopsis.

Members of the SAWADEE class, which are represented by only a single protein in grape, contain a 130-140 amino acids, conserved region located carboxyl-terminal to the HD. This class is characterized by the presence of a 10 amino acid insertion between the second and third alpha-helix (Supplementary Fig. S1). The SAWADEE domain includes several conserved cysteine and histidine residues, suggesting that it participates in metal binding^12^.

PINTOX-class HB proteins, also represented by only a single gene in grape, contain a ~70-amino acids, highly conserved, basic domain located carboxyl-terminal to the HD.

The KNOX and BEL family HB proteins belong to the TALE superfamily, represented by 21 genes in grape. TALE proteins are distinguished by three extra amino acid residues between the first and second helix of the HD^38,39^ (Supplementary Fig. S1). The KNOX family comprises two subfamilies, KNOX I and KNOX II^8^. Proteins included in these families exhibit a conserved KNOX domain amino-terminal to the HD^39^. In addition, they contain a short motif designated ELK immediately adjacent to the HD^2^.

The BEL class is characterized by a conserved, 10-amino acids motif designated ZIBEL, located carboxyl-terminal and N-terminal of the BEL class, as well as in the HD-ZIP II class. Furthermore, we identified a POX domain, located amino-terminal to the HD.

### Expansion patterns of HB genes in grape

**Figure 4:**
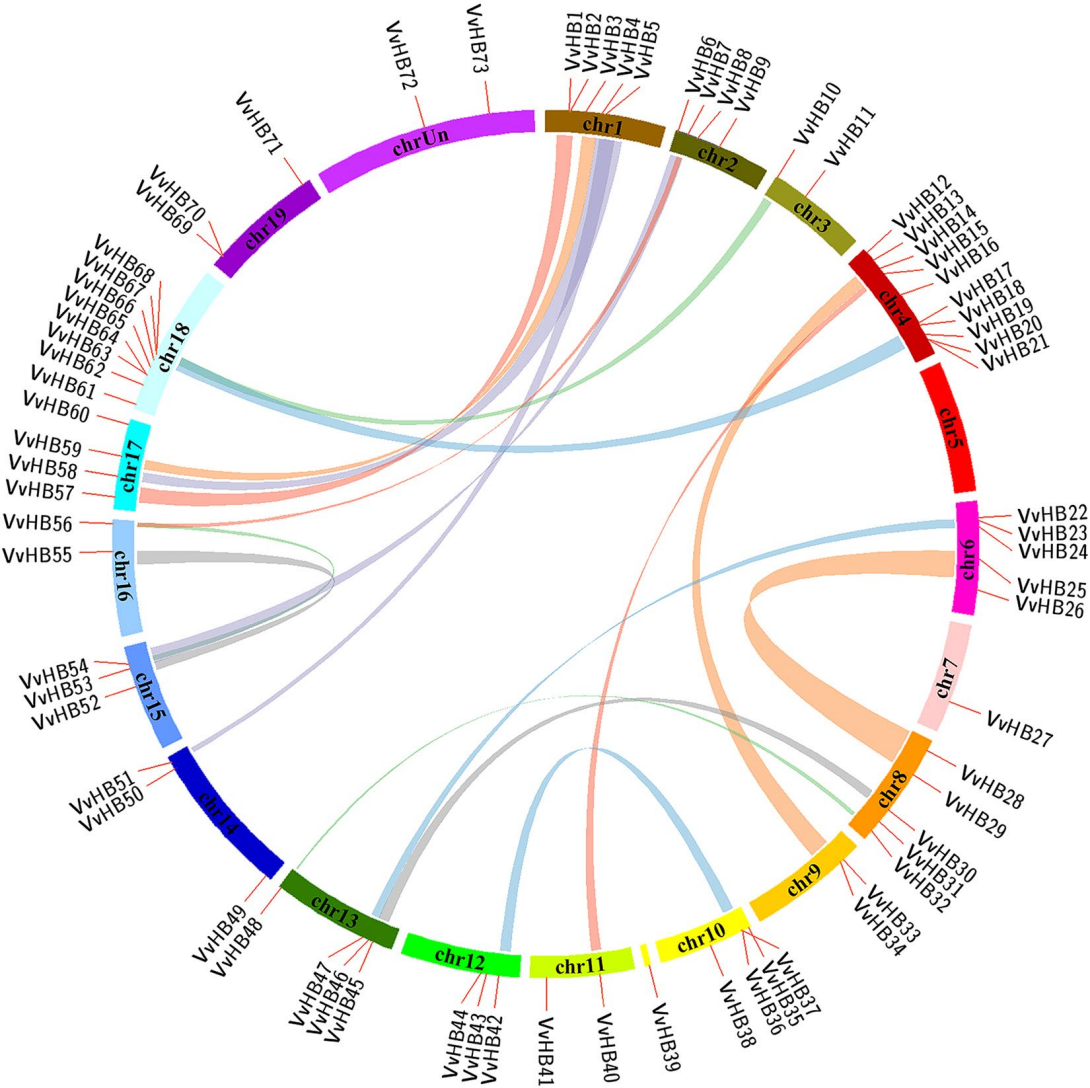
Synteny analysis and chromosomal distribution of grape HB genes. Locations of the grape genes are indicated by orange lines on the grape chromosomes. Colored bars connecting two chromosomal regions denote syntenic regions and the corresponding genes on two chromosomes were regarded as segmental duplications. Chr, chromosomes.

Tandem (two or more genes located on the same chromosome) and segmental duplications (duplicated genes present on different chromosomes), identified based on chromosomal locations, lead to expansion of gene families^40^. In order to gain a better understanding about the evolutionary relationships among members of the grape HB superfamily, the chromosomal locations of the 73 *VvHB* genes were determined. *VvHB* genes are distributed unevenly over 19 of the 20 grape chromosomes (none exists on chromosome 5). For example, 10 *VvHB* genes are located on chromosome 4, whereas only a single *VvHB* gene presents on chromosome 7 (Fig. 4). A chromosomal region within 200 kb containing two or more genes implicates a tandem duplication event^40^. Using this definition, a total of six tandem duplication events were identified (*VvHB1/VvHB2, VvHB20/VvHHB21, VvHB36/VvHB37, VvHB63/VvHB64, VvHB67/VvHB68*, and *VvHB69/VvHB70*), on chromosomes 1, 4, 10, 18, 18 and 19, respectively (Supplementary Table S3). Interestingly, on chromosomes 1, 10, 18 and 19, the *VvHB* genes occur in tandem duplications, however belong to different subfamilies, suggesting that following tandem duplications, sequences of these genes have been altered to a large extent, and protein diversification can be obtained through the reorganization of domains. Therefore, these genes may have lost their original functions or gained new ones after tandem duplications.

Furthermore, as shown in Supplementary Table S4, we also identified 15 pairs of *VvHB* genes associated with segmental duplications (*VvHB48*/*VvHB32*, *VvHB59*/*VvHB3*, *VvHB58*/*VvHB4*, *VvHB57*/*VvHB2*, *VvHB66*/*VvHB17*, *VvHB67*/*VvHB10*, *VvHB55*/*VvHB53*, *VvHB51*/*VvHB5*, *VvHB40*/*VvHB15*, *VvHB47*/*VvHB23*, *VvHB33*/*VvHB13*, *VvHB7*/*VvHB54*, *VvHB7*/*VvHB56*, *VvHB42*/*VvHB36* and *VvHB54*/*VvHB56*). In addition, each pair of *VvHB* genes involved in segmental duplications belongs to the same subfamily. Combining this observation with the results of phylogenetic analyses, it appears that some of these gene pairs (such as *VvHB48*/*VvHB32*, *VvHB59*/*VvHB3, VvHB58*/*VvHB4*, *VvHB57*/*VvHB2* and *VvHB66*/*VvHB17*), clustering in the same phylogenetic subclade (Fig. 2a), may have the same origin. In summary, half of the *VvHB* genes participated in either segmental or tandem duplication events which may provide a reference for the evolutionary relationship and functional prediction of *VvHB* genes.

### Evolutionary relationships between grape and Arabidopsis HB genes

**Figure 5:**
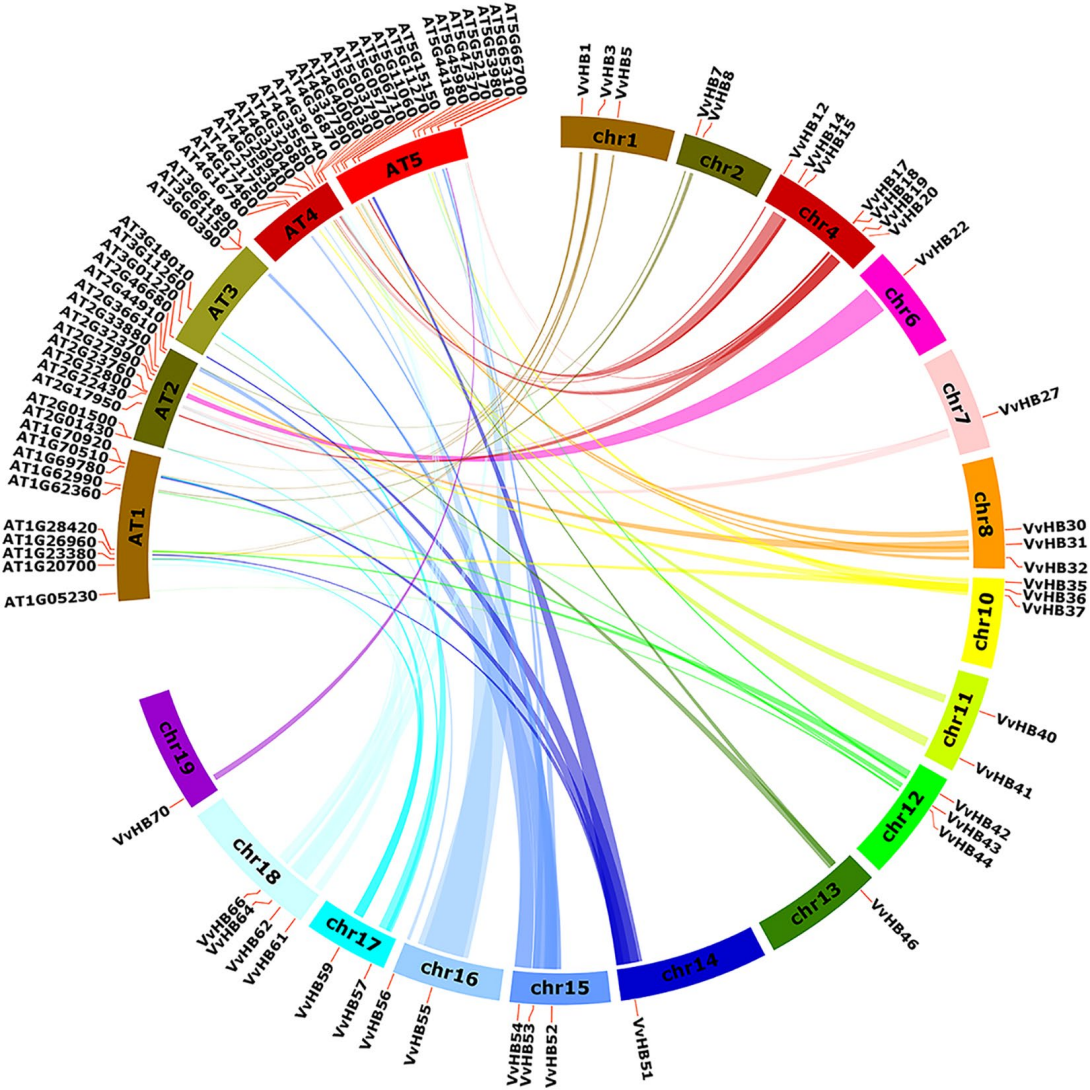
Analysis of synteny of grape HB genes between grape and Arabidopsis. The locations of grape and Arabidopsis genes are indicated by orange lines on respective chromosomes. Colored bars denote syntenic regions between grape and Arabidopsis genes. Chr, chromosomes.

The function of HB genes has been most extensively studied in Arabidopsis. To further clarify the origin, evolutionary process and potential function of grape HB genes we created a comparative synteny map between grape and Arabidopsis HB genes. A total of sixty-four pairs of syntenic relationships were identified, including 54 *AtHB* genes and 39 *VvHB* genes (Fig. 5). The large number of syntenic events suggested that a large-scale expansion have occurred before the divergence of Arabidopsis and grape. Among these pairs, 17 were single, grape/Arabidopsis HB gene correspondences, such as *VvHB61-AT1G20700*, *VvHB43-AT1G62360, VvHB30-AT5G02030* and *VvHB19*-*AT2G17950*. This indicates these genes should have been in the genome of the last common ancestor of grape and Arabidopsis. There were 19 pairs where a single grape gene corresponded to more than one Arabidopsis gene, such as *VvHB62-AT4G40060/AT5G65310, VvHB52-AT3G60390/AT4G16780/AT4G17460/AT2G44910/AT5G47370, VvHB18-AT4G36740/*AT5G66700 and *VvHB55-AT4G25530/AT5G52170*. In contrast, nine pairs of grape HB genes were found that corresponded to only one Arabidopsis HB gene, such as *VvHB7/VvHB54/VvHB56*-*AT2G46680*, *VvHB40/VvHB15-AT4G32040, VvHB51/VvHB5-AT1G26960* and *VvHB62/VvHB27-AT5G65310* (Supplementary Table S5).

Additionally, relating these syntenies to the phylogenetic analysis (Fig. 1), we discovered each pair of genes from grape and Arabidopsis belongs to the same subfamily (Supplementary Table S5), some of these gene pairs (such as *VvHB36-AT1G28420, VvHB8-AT1G62990, VvHB1-AT2G01430, VvHB19-AT2G17950, VvHB27-AT2G22430, VvHB64-AT2G22800, VvHB17-AT2G23760* and *VvHB22-AT2G27990*) even cluster in the same phylogenetic subclade, suggesting that many *VvHB* genes share a common ancestor with their Arabidopsis counterpart, and that they may have the closest genetic relationship. These results could provide a reference for possible functional relevance of *VvHB* genes.

### Expression patterns of grape HB genes in different tissues

**Figure 6:**
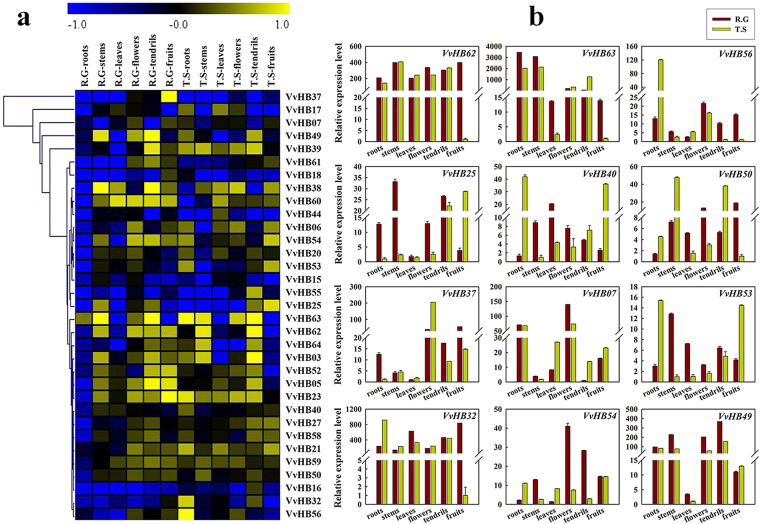
Tissue-specific expression analysis of grape HB genes. Seedless grape cultivar ‘Thompson Seedless’ is denoted as ‘T.S’ and seeded grape cultivar ‘Red Globe’ is denoted as ‘R.G’. (**a**) Semi-quantitative RT-PCR analysis. For each gene, yellow and blue color scale indicates high and low expression levels respectively. Transcripts were normalized to the expression of the *ACTIN1* gene and *EF1-α* gene, and results are shown in Supplementary Figs S3 and S5. Semi-quantitative RT-PCR was analyzed using GeneTools software, and expression values were normalized based on the mean expression value of each gene in all tissues. The heat map was analyzed using MeV 4.8 software. (**b**) Real-time PCR validation of twelve genes (9 differentially expressed genes and 3 randomly selected genes) expressed in different tissues. Transcripts were normalized to the expression of the *ACTIN1* gene; the mean ±SD of three biological replicates is presented.

The known functions of HB genes in other plants suggest that HB genes may play important roles in grape growth and development. Previous transcriptome analysis of grape ovules/seeds at three developmental stages, comparing seedless and seeded grape varieties, identified a large number of differentially expressed genes^25^. Based on transcriptome data (Supplementary Table S6), many grape HB genes showed higher expression levels in seedless grape cultivars relative to seeded cultivars. On this basis, we chose 33 differentially expressed *VvHB* genes for futher analysis. To gain insight into the putative functions of *VvHB* genes in grape growth and development, semi-quantitative RT-PCR was carried out to evaluate expression patterns of these 33 grape HB genes, in six distinct grape tissues (root, stem, leaf, flower, tendril and fruit) of the seedless cultivar ‘Thompson Seedless’ and seeded cultivar ‘Red Globe’ (Fig. 6a and Supplementary Fig. S3). Additionally, a total of 12 *VvHB* genes (9 differentially expressed genes and 3 randomly selected genes) were selected to corroborate their expression levels through quantitative real-time RT-PCR analysis (Fig. 6b).

We found that all the 33 *VvHB* genes were expressed in at least one of the six grape tissues, and the expression levels of most *VvHB* genes (like *VvHB40* and *VvHB50*) varied among the tissues tested, or varied strikingly between ‘Thompson Seedless’ and ‘Red Globe’. Specially, *VvHB37* showed low expression levels in all tissues of ‘Thompson Seedless’ but a moderate level in fruits of ‘Red Globe’. On the contrary, *VvHB23* and *VvHB21* showed high expression levels in most of the tissues. *VvHB61* exhibited predominant expression in reproductive tissues of ‘Red Globe’. In addition, several genes showed distinct, tissue-specific expression, including *VvHB49* which showed a low expression level in leaves in both ‘Thompson Seedless’ and ‘Red Globe’, and *VvHB63* which was not expressed in leaves and fruits in both cultivars. This phenomenon may be due to some species-specific variations. Several genes (such as *VvHB27, VvHB40, VvHB58* and *VvHB59*) exhibited similar expression levels in both ‘Thompson Seedless’ and ‘Red Globe’. This may showed that some genes exhibited conserved expression patterns between the two cultivars. Taken together, these results revealed both similarities and differences in expression patterns between two cultivars.

Combining these results with syntenic blocks and phylogeny reconstructions, we found that some homologous and segmental duplication genes (such as *VvHB40* and *VvHB15*) showed a similar expression pattern, a relatively weak expression level in all tissues. However, other segmental duplication genes showed converse expression patterns. For instance, for the gene pair *VvHB53*/*VvHB55*, *VvHB53* showed relatively strong expression in most organs, while its homolog *VvHB55* exhibited low expression in almost all tissues. For the gene pair *VvHB54*/*VvHB7*, *VvH*B54 was expressed with obviously higher levels in most tissues. The divergences in expression patterns between homologous and segmental duplication genes indicated that some of them may lose function or obtain new function after polyploidy and duplication in evolutionary process.

### Expression patterns of grape HB genes during ovule/seed development

**Figure 7:**
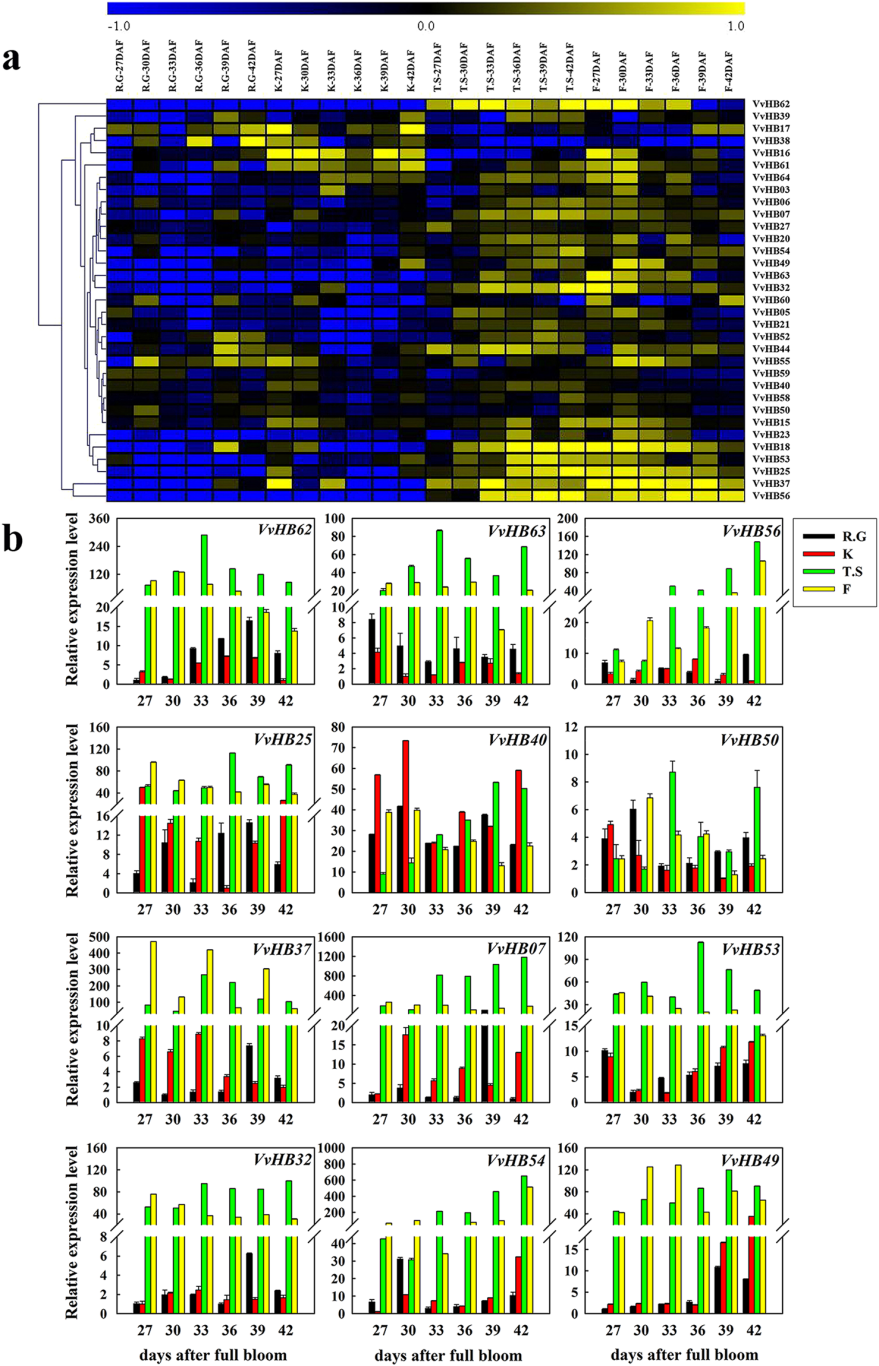
Expression profiles of grape HB genes during ovule development in four grape cultivars. Two seedless grape cultivars, ‘Thompson Seedless’ and ‘Flame Seedless’, are denoted as ‘T.S’ and ‘F’ respectively. Two seeded grape cultivars, ‘Red Globe’ and ‘Kyoho’, are denoted as ‘R.G’ and ‘K’ respectively. (**a**) Semi-quantitative RT-PCR analysis. For each gene, yellow and blue color scale indicates high and low expression levels, respectively. Numbers indicate the number of days after full bloom (DAF). Transcripts were normalized to the expression of the *ACTIN1* gene and *EF1-α* gene and the results are shown in Supplementary Figs S4 and S5. Semi-quantitative RT-PCR measurements were analyzed using GeneTools software, and data was normalized based on the mean expression value of each gene in all development stages. The heat map was analyzed using MeV 4.8 software. (**b**) Real-time PCR validation of twelve genes (9 differentially expressed genes and 3 randomly selected genes) expressed during ovule development in four grape cultivars. Transcripts were normalized to the expression of the *ACTIN1* gene; the mean ± SD of three biological replicates is presented.

To further identify potential roles for *VvHB* genes in ovule development, semi-quantitative RT-PCR was carried out to analyze the expression of these 33 selected *VvHB* genes during ovule development at 27, 30, 33, 36, 39 and 42 days after flowering (DAF), in ‘Red Globe’, another seeded cultivar, ‘Kyoho’, ‘Thompson Seedless’, and additional seedless cultivar, ‘Flame Seedless’ (Fig. 7a and Supplementary Fig. S4). A subset of 12 *VvHB* genes (9 differentially expressed genes and 3 randomly selected *VvHB* genes) were selected to corroborate the results of the semi-quantitative RT-PCR through quantitative real-time RT-PCR analysis (Fig. 7b).

As shown in Fig. 7, several genes (such as *VvHB59, VvHB40, VvHB50* and *VvHB58*) showed similar expression levels in different grape cultivars and displayed no obvious changes in different ovule developmental stages, suggesting that these genes are functionally conserved in all grape cultivars and may be crucial for ovule development. However, most of the *VvHB* genes, including *VvHB62, VvHB63, VvHB25, VvHB7, VvHB53, VvHB49, VvHB54, VvHB32, VvHB18, VvHB37* and *VvHB56*, exhibited higher expression levels in seedless cultivars relative to seeded cultivars. In particular, *VvHB62*, *VvHB56* and *VvHB54* showed remarkable differences in the expression patterns during ovule development between seeded and seedless grape cultivars, with expression almost undetectable in seeded cultivars. These results indicated that these genes may participate in ovule abortion. In contrast, two genes, *VvHB38* and *VvHB17*, showed higher expression levels in seeded cultivars relative to seedless cultivars, suggesting that they may be related to the normal ovule development process. Taken together, we propose that there is a close relationship between *VvHB* genes and grape stenospermocarpy.

## Discussion

HB gene family members play varied and important roles in plants including in embryogenesis^17^, response to ABA^41^ and abiotic stress^42,43^. Many HB TFs have been analyzed in a variety of plants, such as Arabidopsis^12^, poplar^12^, rice^44^ and legumes^35^. However, there have been few studies of HB genes in grape. In the present study, we identified a total of 73 grape HB genes and analyzed their evolutionary relationships, domain architectures and potential functions. Furthermore, we analyzed the expression profiles of 33 key *VvHB* genes in various grape organs and ovule developmental stages to clarify the importance of the *VvHB* genes in the growth and development of grapes.

HB genes can be found in diverse plant species, including angiosperms, bryophyta and lycophytes^12^. The HD consists of an alpha- helix I which is 12 residues length, a connecting loop of 6 amino acid residues leading to helix II (11 aa length), a tight turn of three residues, a helix III (11 aa length) and a flexible and disordered 7 aa length helix IV^45^. We found that almost all of the 73 grape HB genes encode proteins with a complete HD, with the exception of *VvHB54*, in which the first alpha-helix is absent. Previous studies have indicated that helices II and III form a helix-turn-helix motif that is required for regulatory function of the proteins^46^. This means that the second and the third helices may be the most important and necessary, and may similarly influence the functions of eukaryotic genes. Additionally, we found that *VvHB54* showed substantial expression levels in various organs, suggesting that it is not a pseudogene.

During the evolution process of land plants, gene duplication, including tandem duplications, segmental duplications and whole genome duplications (WGDs), has played an important role in genomic expansions and rearrangements^47,48^. Whole genome duplications have recurred in many lineages of the angiosperms^49^, leading to remarkable differences in genome sizes. Synteny and collinearity analyses of plant genomes indicated that an ancient genome triplication (γ-triplication) event occurred in the common ancestor of grape and Arabidopsis, leading to a paleohexaploid^50^. Following the γ-triplication event, two recent paleopolyploidy events, β-duplications and α-duplications occurred in Arabidopsis. However in grape, there was only the common γ-triplication event and no subsequent WGDs^27^. The ancient polyploidization events could influence the number of genes in multiple gene families, through gene loss or expansions^51,52^. These evolutionary relationships might lead to the expansion of ancestral genes in Arabidopsis. Therefore the number of HB genes between Arabidopsis and grape showed a relatively large difference. Because the grapevine genome had not undergone the recent whole genome duplications, tandem duplications and segmental duplications would be the main causes of gene family expansions in grape. In this study, a total of 21 gene pairs from grape HB genes were identified as products of tandem or segmental duplication events (Fig. 4 and Supplementary Tables S3 and S4), consistent with findings in soybean whereby a total of 246 (89.1%) HB genes were found located on duplicated chromosomal blocks^35^, suggesting that tandem and segmental duplications likely played a crucial role in the expansion of the HB gene family in plants. Combining synteny analysis with phylogenetic analysis, we found most of the grape HB genes that clustered in the same phylogenetic subclade were segmental or tandem duplications. Some orthologous gene pairs, such as *VvHB56*/*VvHB7* and *VvHB40*/*VvHB15*, resulting from segmental duplications, showed similar expression patterns during ovule development stages. Additionally, expression patterns of these orthologous gene pairs were more similar than other orthologous pairs that only clustered together in the phylogenetic tree or were syntenic orthologs. However, regarding other segmental duplication orthologous gene pairs, such as *VvHB55*/*VvHB53*, showed the distinctly different transcript levels during ovule development stages. It seems possible that high sequence similarity is not necessarily correlated with similar transcript levels, because they may perform similar biochemical functions in different tissues or periods during plant growth and development. Differences in gene expression pattern were considered to arise through duplication in the evolutionary process. Functional diversification of these segmental duplication orthologous genes was also considered a major feature of the long-term evolution of polyploids^53^. Following gene duplication, functional differentiation may result from pseudogenization, conservation of gene function, non-functionalization and sub-functionalization^54^. Additionally, we also identified 64 gene pairs involved in segmental duplications between grape and Arabidopsis HB genes, suggesting they might have a common ancestor. Based on the reported function of their Arabidopsis homologs, we can speculate the possible functions of these orthologous pairs in grape. Taken together, these results enable further analyses of evolution and potential functions among these genes, and are useful for further study on the functions of homologous genes in different plant species.

Interestingly, we found highly variable numbers of genes on different chromosomes. Chromosome 4 contained 10 *VvHB* genes while chromosome 5 had none. These results indicated that duplication of *VvHB* genes likely occurred in chromosome 4 during the evolution of gene families, which might be associated with gene functions.

During the evolutionary process, various organisms had been participated in gene duplication events, after which gene loss and gene sub-functionalization can occur frequently. Gene loss is a common phenomenon during the evolution of the multigene families. Following gene loss, neo-functionalization and sub-functionalization contributed to the retention of new genes. In this study, gene loss observed in the PLINC, NDX and LD clades is expected to decrease functional redundancy and define the key *VvHB* genes^55^. Alternatively, our data is not inconsistent with these three groups representing Brassicaceae-specific HB gene lineages.

In this study, in order to further define the evolutionary relationship within grape HB genes, we identified 20 highly conversed motifs among the 73 HB proteins. Different motifs were conserved within every group, and motifs within each subgroup tended to exhibit a similar distribution pattern. These results supported the phylogenetic relationship between different HB genes in grape (Fig. 2a). In addition, these motifs were also detected in Arabidopsis, and the distribution pattern was similar between grape and Arabidopsis, suggesting that these motifs were highly conserved during evolution, which could provide a possible functional relevance. The evolution of gene family members could also be analyzed by exon/intron diversification, which includes exon/intron gain/loss, insertion/deletion and exonization/pseudoexonization^56^. In this study, the 73 *VvHB* genes exhibited between 0 and 22 introns. A total of 62 of the genes (85%) have zero to ten introns, whereas only 11 genes have more than 10 introns. Overall, genes within the same group had similar numbers of introns. However, there were exceptions: *VvHB17*, which had twelve exons, is orthologous to *VvHB66* which has only five exons. In general, the gain/loss of the exons or introns may be the main reason for the chromosomal rearrangement and fusion^25^ and the differences of the functions of the grape HB proteins, contributing to functional diversification of these genes. Furthermore, divergence in exon/intron length could also potentially lead to the generation of functionally distinct paralogs or orthologous. Taken together, the grape HB genes show highly similar structures of exons/introns and conserved motifs distribution in the same subgroup.

The HD-ZIP subfamily is the largest group of HB proteins in grape, and contains HD-ZIP I, HD-ZIP II, HD-ZIP III, and HD-ZIP IV. The number of genes in HD-ZIP I (13) is similar to that in Arabidopsis (17) (Supplementary Table S1). Interestingly, genes in HD-ZIP III are longer and contain more exons (Fig. 2c). The number of genes in HD-ZIP IV is smaller than that in Arabidopsis, suggesting that genes included in the HD-ZIP IV subfamily may not be strongly conserved. Arabidopsis *AtHB8* belongs to HD-ZIP III, was expressed in procambial cells of the embryo and developing organs^57^, and played a role in vascular development and differentiation^58^. *AtHB8* is clearly homologous to grape *VvHB13*, suggesting that *VvHB13* may have the similar function as its Arabidopsis counterpart. In addition, *VvHB23*, which belongs to this subgroup, showed differential expression during ovule development between seedless and seeded grape varieties. Other subfamilies of HB genes also have been demonstrated to be important. Genes in the WOX subfamily have been reported to play an important role in early embryogenesis in Arabidopsis^33^. Moreover, WOX family genes have been shown to play a key role in coordinating gene transcription involved in the early phases of embryogenesis in grape. In a previous study, the *VvWOX* genes showed different expression profiles during somatic embryogenesis^59^. During somatic embryogenesis, *VvWOX2* and *VvWOX9* were the most important WOX genes, while *VvWOX3* and *VvWOX11* showed strong expression in the torpedo and cotyledonary stages, but weak expression in the earlier developmental stages. In this study, *VvHB37*, which belongs to the WOX subfamily, showed dramatically higher expression levels during different development stages of ovules in seedless grape cultivars (Fig. 7b). Consistent with previous research, these results suggest that this subfamily may play an important role in embryogenesis. In addition, the WOX family genes in grape also showed various expression profiles in different grapevine tissues^60^, which has also been found in this study. The TALE family includes two families, KNOX and BELL, and the KNOX family is composed of the smaller KNOX I and KNOX II families, which are highly conserved between monocots and dicots^61^. Additionally, members of the small PHD family have been shown to participate in chromatin-mediated transcriptional regulation^62^.

HB genes have been widely studied in legumes^35^ and tomato^15^, and their expression profiles and potential functions during development have been documented. In a recent study, a tomato HD-ZIP I family gene designated *LeHB1* was shown to play key roles in carpel development and fruit maturation^15^. *MdHB1*, the homologous gene of *LeHB1* in apple, has been reported to be involved in the regulation of anthocyanin accumulation^63^. In our study, some *VvHB* genes (*VvHB62*, *VvHB54* and *VvHB7*) belonging to the HD-ZIP I subfamily showed markedly differential expression during ovule development between seedless and seeded grape. Compared to climacteric fruit, such as tomato and apple, there are relatively few studies of non-climacteric fruits like grape. Moreover, the characterization of Arabidopsis HB genes allows for prediction of potential functions of the *VvHB* genes. Besides HB genes, there are many other genes associated with seed morphogenesis in grapevine. For example, *VviAGL11*, a class D MADS-box transcription factor, exhibited relatively high expression levels in seeds at 2, 4 and 6 weeks after fruit set, relative to seedless grape at any developmental stage^64^. The metacaspase gene family of *V. vinifera*, containing six genes, also showed differential expression profiles during ovule abortion in stenospermocarpic seedless grapes^65^. Moreover, there were some other genes in different plant species also related to ovule development. In petunia, the floral binding protein 11 (FBP11) MADS box gene was found only expressed in ovule primordia and subsequently showed a role in ovule formation^66^.

We documented the expression of 33 *VvHB* genes in six organs and six developmental stages of the ovule in distinct grape cultivars, revealing their potential functions in grape growth and development. For example, *VvHB37, VvHB62* and *VvHB63* showed extremely low expression levels in nearly all ovule developmental stages in seeded grape cultivars, but were expressed to relatively high levels in seedless grape cultivars (Fig. 7b), suggesting a role in ovule abortion of seedless grape cultivars. In addition, as demonstrated in a previous study^25^, a substantial number of the 33 selected genes, such as *VvHB25, VvHB7, VvHB53, VvHB49, VvHB54, VvHB32* and *VvHB18*, showed higher expression levels in seedless grape cultivars relative to seeded grape cultivars. We also identified a subset of genes, such as *VvHB38* and *VvHB17*, which showed opposite expression profiles in ovules between seeded and seedless grape cultivars, being expressed more strongly in ovules of seeded grape cultivars (Fig. 7a). This suggests that these two genes may have a function in normal seed development. These results will facilitate the functional analysis of these genes and provide new resources for molecular breeding of seedless grapes. Of course, future studies may address how these HB genes may contribute to the seedless trait.

Interestingly, we found that three genes, *VvHB62, VvHB63* and *VvHB56*, showed low expression levels in fruits of ‘Thompson Seedless’, but high expression levels in ovules. In order to illustrate this phenomenon, the sarcocarp was separated from fruits (at 42 days post anthesis) and ovules (42 days after full bloom) of ‘Thompson Seedless’ and analyzed by real-time RT-PCR (Supplementary Fig. S6). The results showed that these three genes were expressed to lower levels in the sarcocarp than in ovules, consistent with our previous results (Fig. 6 and Fig. 7). Further study can be taken to clarify the reasons that cause these situations.

## Materials and Methods

### Identification and annotation of the grape HB gene family

HB genes in Arabidopsis were as identified in a previous study^12^. Homology with those identified Arabidopsis HB genes was assessed using the NCBI non-redundant protein database. Protein sequence was analyzed for domain structure using PFAM. HB genes in grape were identified by using Hidden Markov Model (HMM) profiles and BLAST-P to search the Grape Genome Database (http://www.genoscope.cns.fr). Sequence integrity of the HD (PF00046) was analyzed using SMART (http://smart.embl-heidelberg.de), and only domains with Expect (e)-values less than ±1e-05 were retained for further analysis^35^. Protein sequences and coding sequences were retrieved from the Grape Genome Database, and SMART software was used to analyze domain structure.

### Multiple sequence alignment and phylogenetic analysis of the grape HB gene

Multiple sequence alignments of a total of 73 grape HD sequences and 110 Arabidopsis HD sequences^12^ were performed using ClustalX 2.1 with default parameters^67^. The phylogenetic tree including sequences from grape and Arabidopsis was constructed using the neighbour-joining (NJ) method^68,69^ and MEGA 5.0 software. Bootstrap analysis was performed using 1000 replicates^70^ with the following parameters: “p-distance”, “Complete Deletion” and gap setting.

### Exon-intron structure analysis, distribution of conserved motifs and characteristic domain architecture in grape HB genes

The exon-intron structures of the grape HB genes were determined based on alignments of transcribed sequences and corresponding genomic sequences, and diagrams were created using the online Gene Structure Display Server 2.0^71^, which showed upstream/downstream sequence, exon/intron position. Conserved motifs and domains of grape HB genes other than the HD were identified using MEME 4.11.2 (http://meme-suite.org/tools/meme) and SMART (http://smart.embl-heidelberg.de) software.

### Synteny analysis of grape HB genes

Tandem and segmental duplications of HB genes in the grape genome were identified based on chromosomal locations. For synteny analysis, adjacent homologous grape HB genes on a single chromosome, without the presence of intervening genes, were defined as tandem duplications. Gene duplication events occuring on different chromosomes were defined as segmental duplications^47^. The list of grape HB genes in duplicated genomic regions, and a comparison of grape and Arabidopsis genomes, were obtained from the Plant Genome Duplication Database^72^. Diagrams were generated using the program Circos version 0.63 (http://circos.ca/).

### Plant material

Grapevine cultivars used in this study included two seeded grapevines, Red Globe (*Vitis vinifera*) and Kyoho (*V. vinifera×V. labrusca*), and two seedless grapevines, Thompson Seedless (*V. vinifera*) and Flame Seedless (*V. vinifera*). Plants were maintained in the grape germplasm resource orchard of Northwest A&F University, Yangling, China (34°20′N 108°24′E). The plant parts collected were young roots, stems, leaves, flowers (at full bloom), tendrils, fruits (42 days after full bloom (DAF). All tissues samples were obtained from 3-year-old ‘Thompson Seedless’ and ‘Red Globe’ plants under natural conditions. Previous studies have reported that seed weight of seedless grapes usually begin to decrease at 27 ~ 33 days after full bloom (DAF)^25,73^. Therefore, ovules samples were collected from berries at 27 DAF, 30 DAF, 33 DAF, 36 DAF, 39 DAF and 42 DAF from two seeded grape cultivars, ‘Kyoho’ and ‘Red Globe’, and two seedless grape cultivars, ‘Thompson Seedless’ and ‘Flame Seedless’. Additionally, all grape samples were immediately frozen in liquid nitrogen and stored at −80 °C for RNA extraction and expression analysis.

### Expression analysis of grape HB genes

Total RNA was extracted from grape samples using an EZNA Plant RNA Kit, according to the manufacturer’s instructions (R6827-01, OMEGA Biotek, USA). A total of 500 ng DNase-treated RNA was subjected to reverse transcription to generate cDNA, using PrimeScript RTase (TransGen Biotech, Beijing, China). Resulting cDNA products were diluted six-fold and stored at −40 °C prior to analysis. We used the grape *ACTIN1* gene (GenBank Accession number NC_012010) with the forward primer (5′-GAT TCT GGT GAT GGT GTG AGT-3′) and reverse primer (5′-GAC AAT TTC CCG TTC AGC AGT-3′), and the grape *EF1-α* gene (GenBank Accession number NC_012012) with the forward primer (5′-AGG AGG CAG CCA ACT TCA CC-3′) and reverse primer (5′-CAA ACC CTG CAT CAC CAT TC-3′) as internal controls.

Gene-specific primers were designed for 33 grape HB genes (Supplementary Table S7) using Primer Premier 6.0. Semi-quantitative RT-PCR was carried out in a volume of 20 μl per reaction containing 1 μl of cDNA template, 1.6 μl of gene-specific primers (1.0 μM), 7.6 μl sterile distilled water and 9.8 μl PCR Master Mix (BIOSCI BIOTHCH CO. LTD, Hangzhou, China). The profile was 94 °C for 2 min, 29–38 cycles of 94 °C for 30 s, 52–63 °C for 30 s and 72 °C for 30 s, with a final extension of 72 °C for 2 min. In each case, 5 μl of the resulting semi-quantitative RT-PCR product was resolved on a 1.5% (w/v) agarose gel, dyed with ethidium bromide, and then photographed under ultraviolet light using GeneSnap software. Each assay was performed with three biological replicates. RT-PCR expression data was visualized using GeneTools software, and the normalized data based on the mean expression value of each gene^73^ in all tissues or ovule developmental stages in all cultivars was log2-transformed to generate heat-maps using MultiExperiment Viewer software (Mev 4.8.1)^72^.

Quantitative real-time PCR analysis was conducted using SYBR Green (TransGen Biotech, Beijing, China) on an IQ5 real time-PCR machine (Bio-Rad, Hercules, CA, USA). The grape *ACTIN1* gene (GenBank Accession number NC_012010) was used as the internal reference genes and each reaction was carried out in triplicate with a volume of 20 μl containing 1 μl of cDNA template, 0.8 μl each primer (1.0 μM), 7.4μl sterile distilled water and 10 μl of SYBR green. PCR was performed following the parameters: 95 °C for 30 s, followed by 40 cycles of 95 °C for 5 s and 60 °C for 30 s. Melting-curve analyses were performed with an intial incubation at 95 °C for 15 s and then a constant increase from 60 °C to 95 °C. For each assay, three independent biological replications were performed. Relative expression levels were analyzed using the IQ5 software and the normalized expression method^73^, visualized using SigmaPlot 10.0.

## Electronic supplementary material

Supplementary Information
