# Publisher Correction: Genome-wide identification and expression analyses of the homeobox transcription factor family during ovule development in seedless and seeded grapes

**DOI:** 10.1038/s41598-017-16207-6

**Published:** 2017-11-17

**Authors:** Yunduan Li, Yanxun Zhu, Jin Yao, Songlin Zhang, Li Wang, Chunlei Guo, Steve van Nocker, Xiping Wang

**Affiliations:** 1 State Key Laboratory of Crop Stress Biology in Arid Areas, College of Horticulture, Northwest A&F University, Yangling, Shaanxi China; 2 Key Laboratory of Horticultural Plant Biology and Germplasm Innovation in Northwest China, Ministry of Agriculture, Northwest A&F University, Yangling, Shaanxi China; 3 Department of Horticulture, Michigan State University, East Lansing, Michigan USA

*Scientific Reports* **7**:12638; doi:10.1038/s41598-017-12988-y; Article published online 03 October 2017

The original HTML version of this Article contained errors in the Results section under subheading ‘Domain architecture analysis of grape HB proteins’.

“We found that the comment = “Style change has been done. Please check.“VvHB proteins represented 11 of these families”.

now reads:

“We found that the VvHB proteins represented 11 of these families”.

“There are two comment = “Style change has been done. Please check.“HB genes encoding HB-PHD proteins in both grape and Arabidopsis”.

now reads:

“There are two HB genes encoding HB-PHD proteins in both grape and Arabidopsis”.

These errors have now been corrected in the HTML version of the Article; the PDF version was correct from the time of publication.

