# Supplementary material for: Genome-wide identification and expression analyses of the homeobox transcription factor family during ovule development in seedless and seeded grapes
Source: Sci Rep. 2017 Oct 3;7:12638. doi: 10.1038/s41598-017-12988-y (PMC5626728; doi:10.1038/s41598-017-12988-y)
Supplement: Supplementary file 1 — Supplementary Information [file 41598_2017_12988_MOESM1_ESM.pdf]

Genome-wide identification and expression analyses of the homeobox transcription factor family during ovule development in seedless and seeded grapes

Yunduan Li[1,2], Yanxun Zhu[1,2], Jin Yao[1,2], Songlin Zhang[1,2], Li Wang[1,2], Chunlei Guo[1,2], Steve van Nocker[3], Xiping Wang[1, 2]*

[1]State Key Laboratory of Crop Stress Biology in Arid Areas, College of Horticulture, Northwest A&F University, Yangling, Shaanxi, China

[2]Key Laboratory of Horticultural Plant Biology and Germplasm Innovation in Northwest China, Ministry of Agriculture, Northwest A&F University, Yangling, Shaanxi, China

[3]Department of Horticulture, Michigan State University, East Lansing, Michigan, USA

* Corresponding author: Xiping Wang

College of Horticulture, Northwest A&F University,

Yangling 712100, Shaanxi, China

E-mail: wangxiping@nwsuaf.edu.cn

Tel: 86-29-87082429

Fax: 86-29-87082613

E-mail addresses of all authors:

YL: yunduan.li@nwsuaf.edu.cn

YZ: yanxun.zhu@nwsuaf.edu.cn

JY: 2014014898@nwsuaf.edu.cn

SZ:18821636061@163.com

LW: valley2013060091@gmail.com

CG: guo0208chun@163.com

SVN: vannocke@msu.edu

Supplementary Figure S1: Alignment of the homeodomain in grape and *Arabidopsis.* Five highly conserved amino acids within the HD (Leu-16, Trp-48, Phe-49, Asn-51, and Arg-53) were indicated.

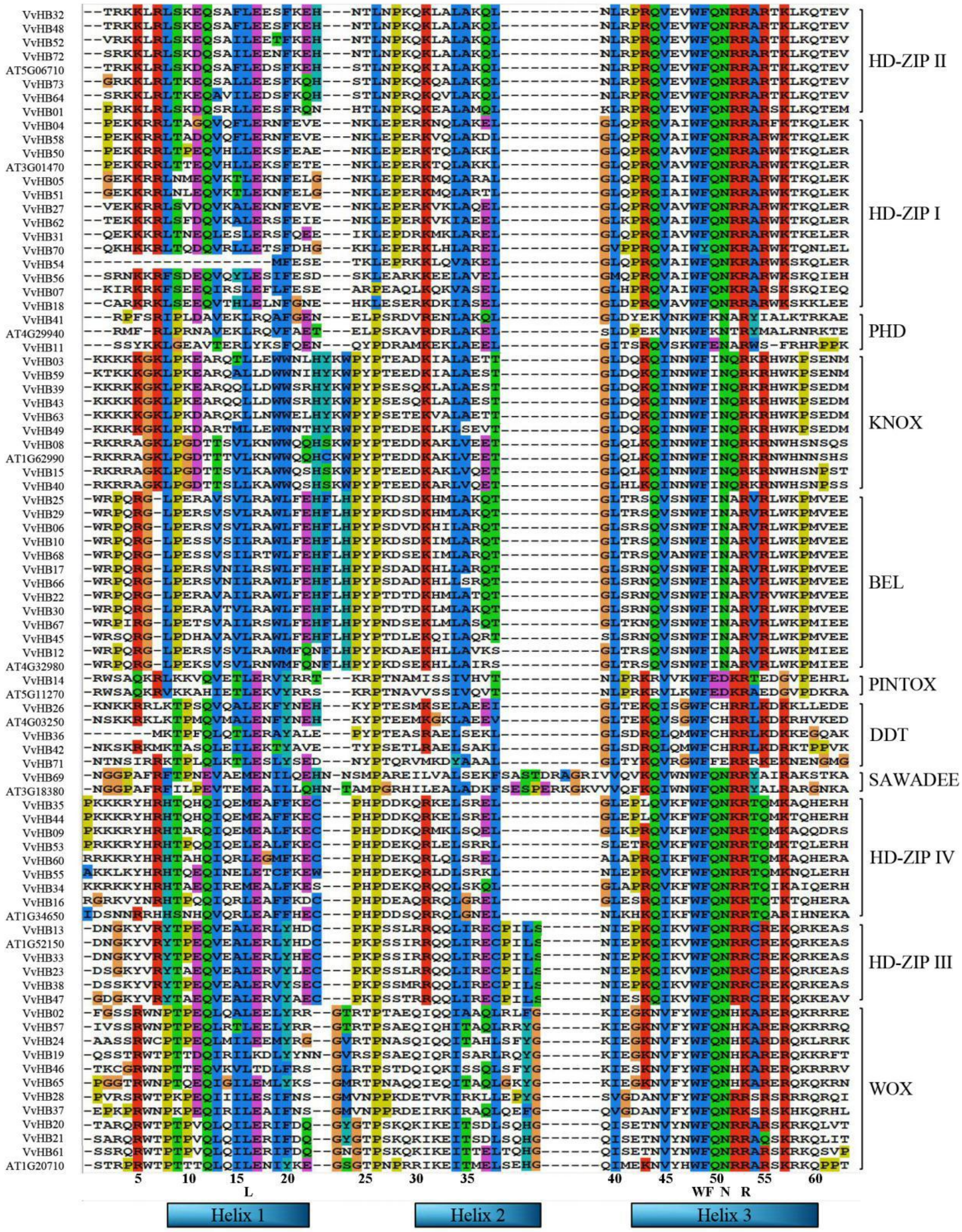

Supplementary Figure S2: Conserved motifs of *Arabidopsis* homeobox genes

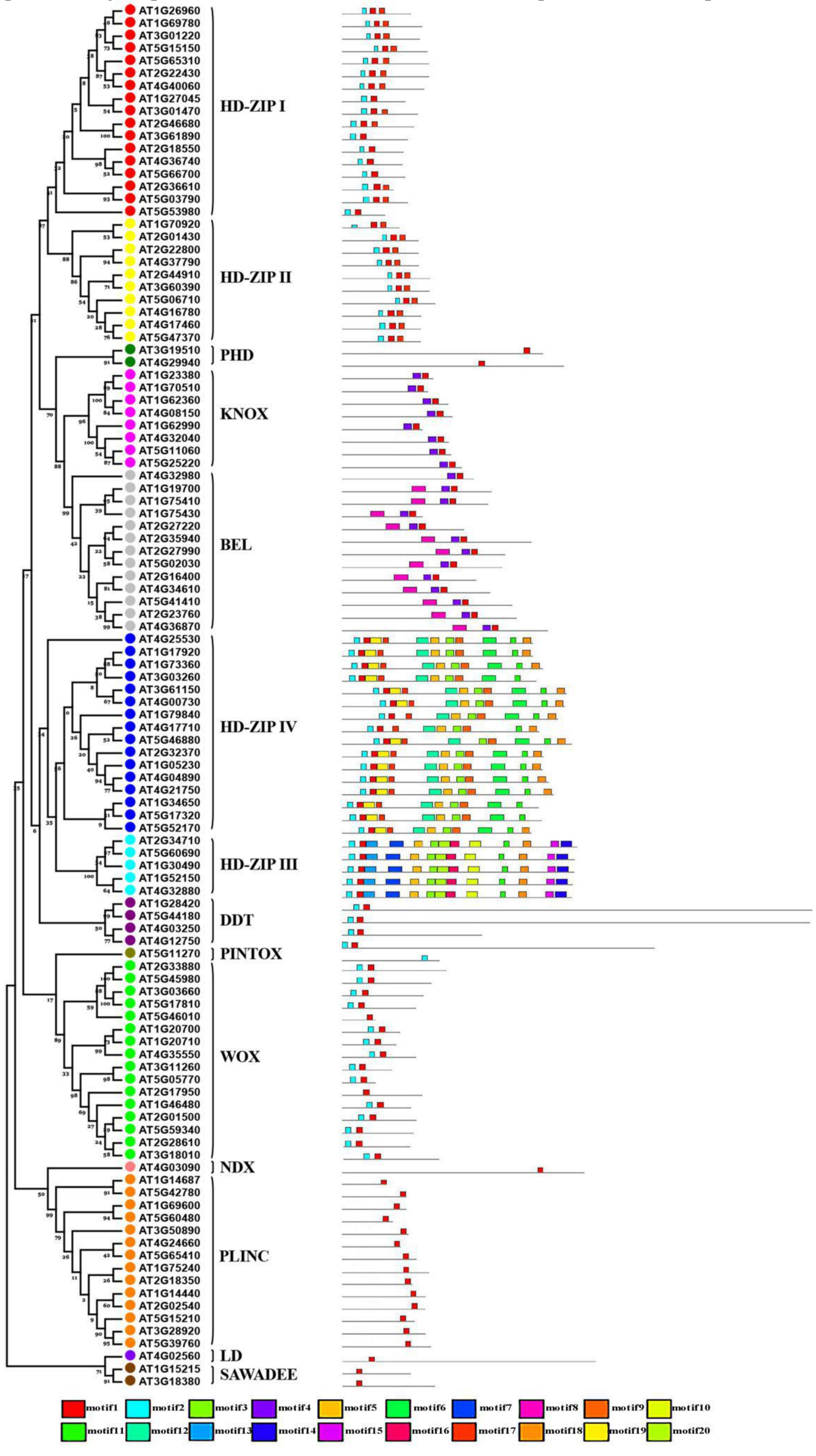

Supplementary Figure S3: Expression profiles of 33 *VvHB* genes in different grape organs measured by semi-quantitative RT-PCR. 'Thompson Seedless' is represented by 'T.S' and 'Red Globe' is represented by 'R.G'. *Actin1* (GenBank Accession number NC_012010) and *EF1-α* (GenBank Accession number NC_012012) were used as internal controls. The grouping of each gene was cropped from different parts of the same gel, including all the tissues from two grape varieties. The exposures of bands in the same gel are consistent and exposures (ms) of each grouping are shown in the figure.

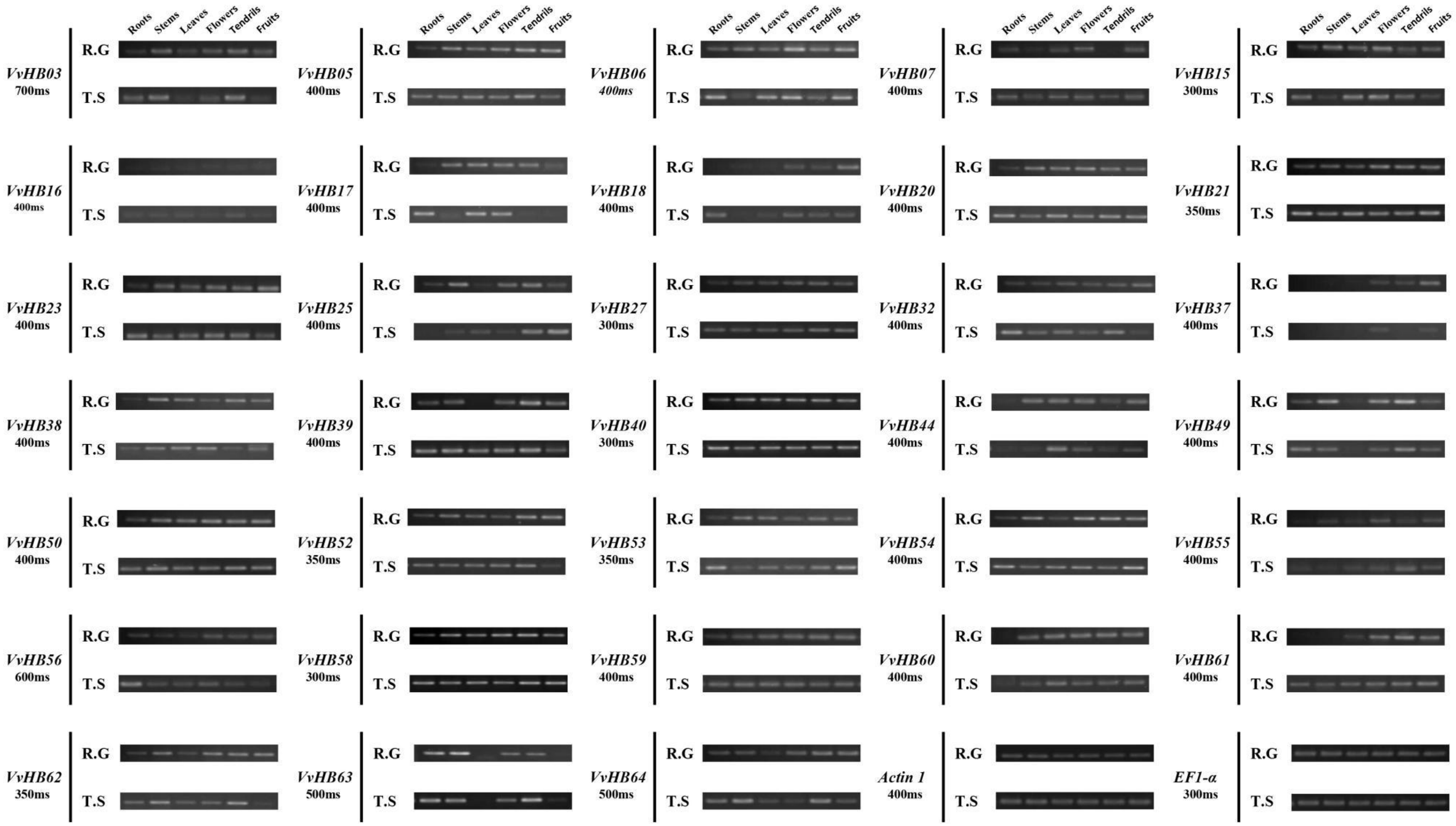

Supplementary Figure S4: Expression patterns of 33 *VvHB* genes in different developmental stages of ovules measured by semi-quantitative RT-PCR. Abbreviations: T.S, Thompson Seedless; R.G, Red Globe; K, Kyoho; F, Flame Seedless. *Actin1* (GenBank Accession number NC_012010) and *EF1-α* (GenBank Accession number NC_012012) were used as internal controls. The grouping of each gene was cropped from different parts of the same gel, including all ovule development stages from four grape varieties. The exposures of bands in the same gel are consistent and exposures (ms) of each grouping are shown in the figure.

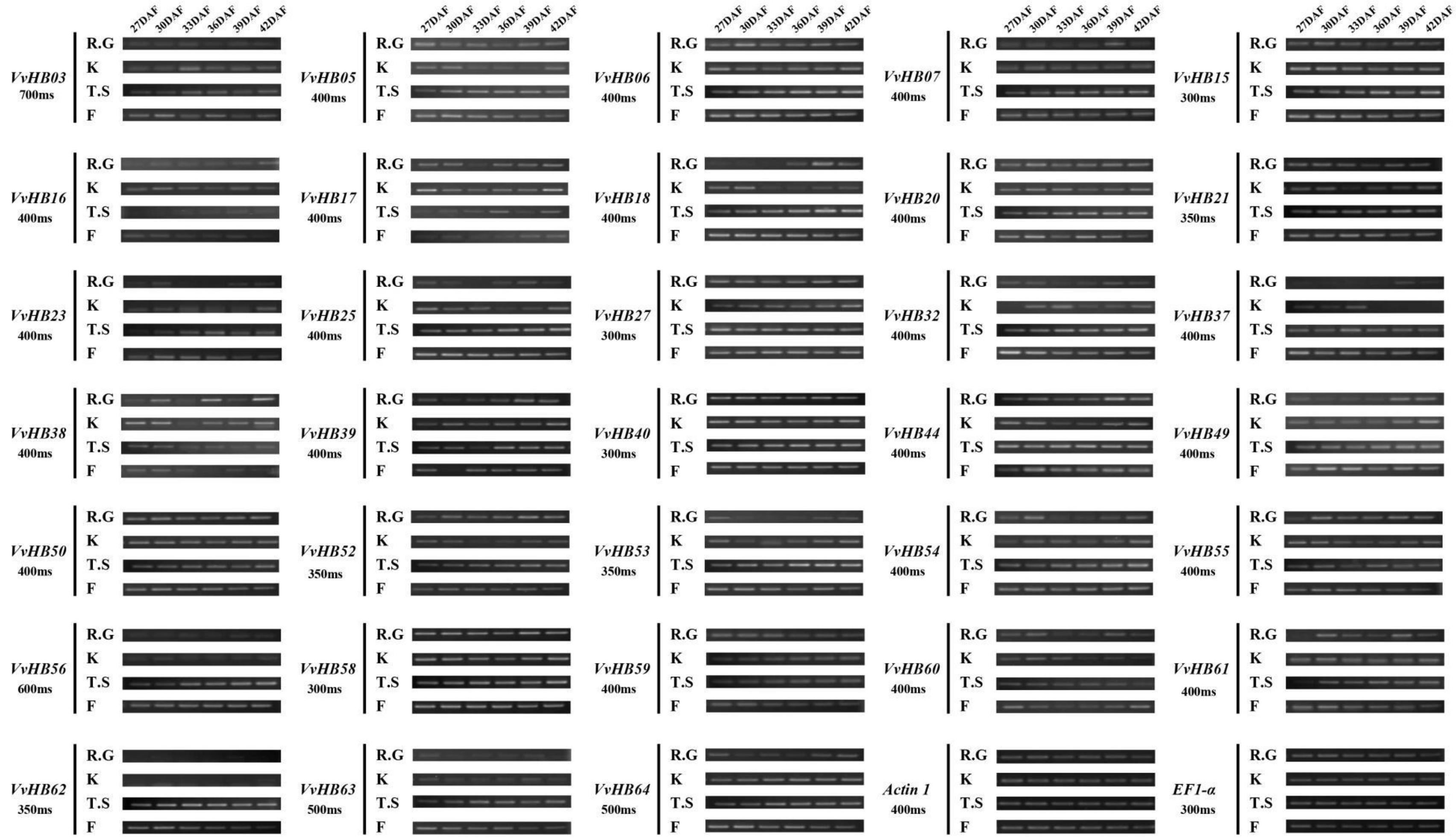

Supplementary Figure S5: The full-length gels of expression patterns performed by semi-quantitative RT-PCR. Abbreviations: T.S, Thompson Seedless; R.G, Red Globe; K, Kyoho; F, Flame Seedless. DAF, after full bloom.

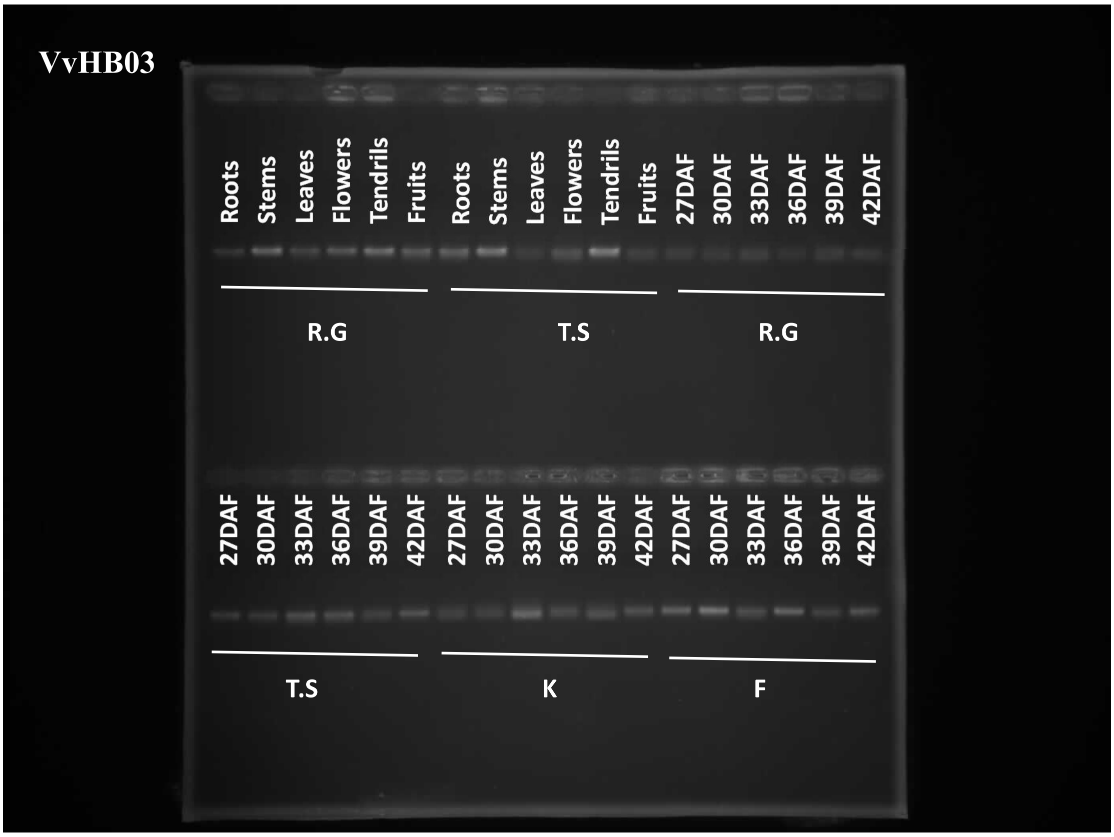

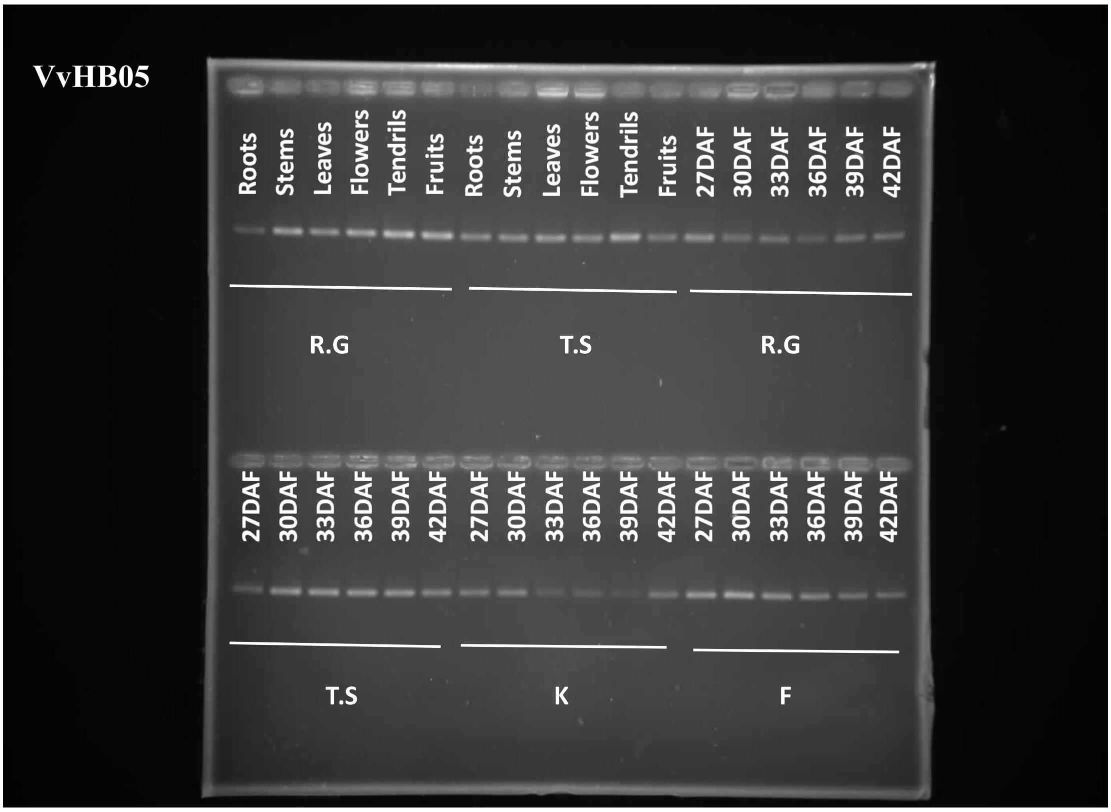

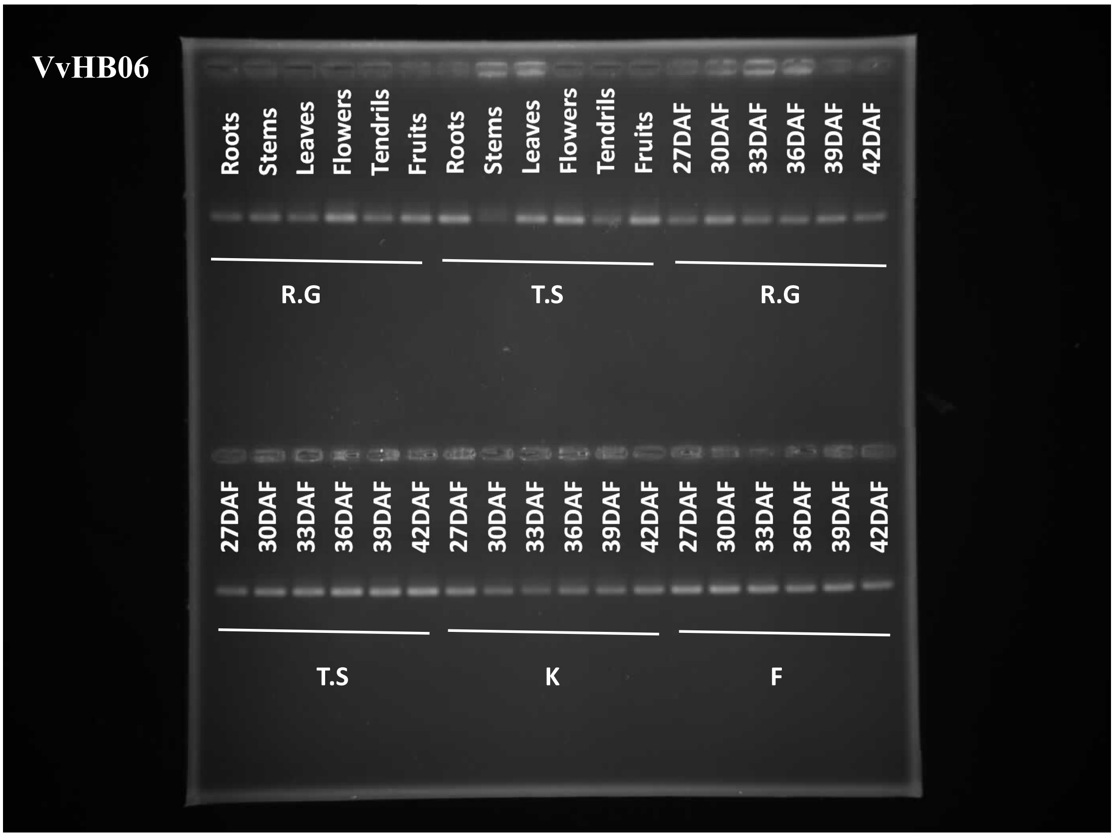
VvHB06
Roots
Stems
Leaves
Flowers
Tendrils
Fruits
Roots
Stems
Leaves
Flowers
Tendrils
Fruits
27DAF
30DAF
33DAF
36DAF
39DAF
42DAF
R.G
T.S
R.G
27DAF
30DAF
33DAF
36DAF
39DAF
42DAF
27DAF
30DAF
33DAF
36DAF
39DAF
42DAF
27DAF
30DAF
33DAF
36DAF
39DAF
42DAF
T.S
K
F

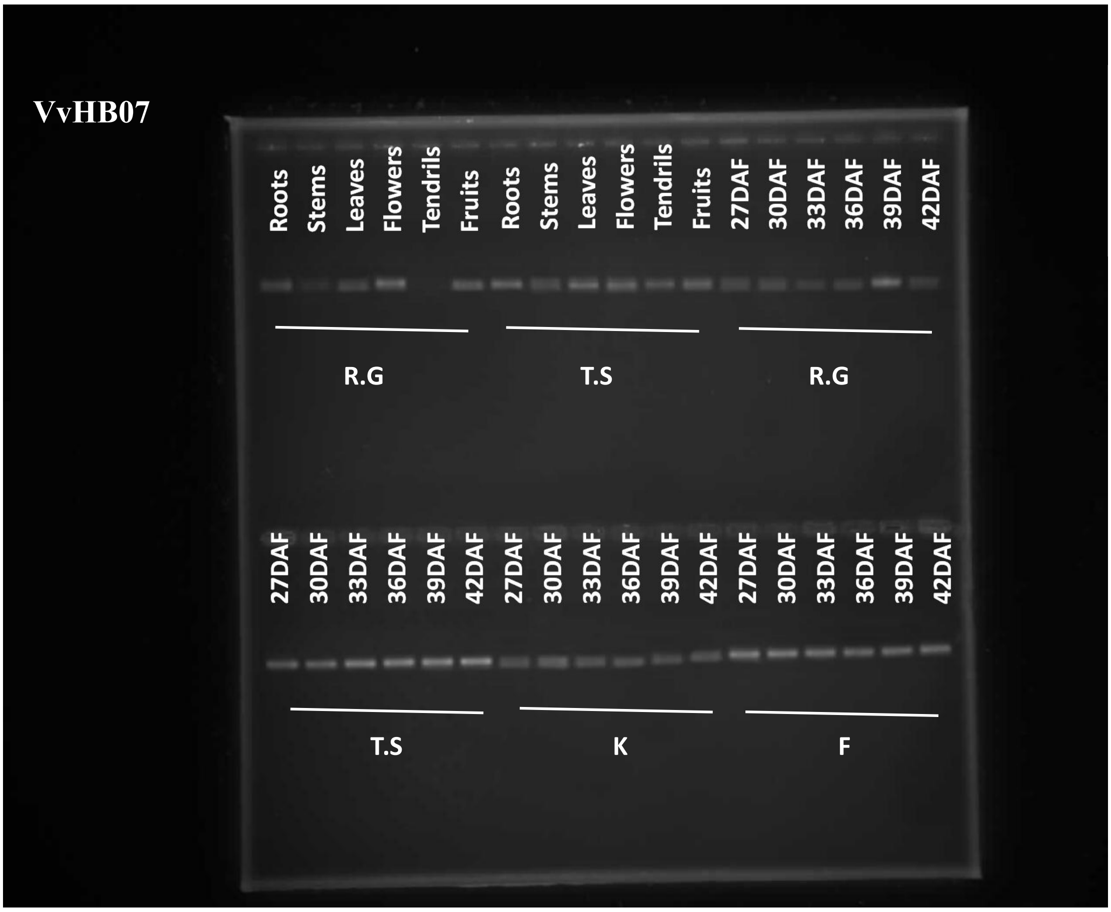
VvHB07
Roots
Stems
Leaves
Flowers
Tendrils
Fruits
Roots
Stems
Leaves
Flowers
Tendrils
Fruits
27DAF
30DAF
33DAF
36DAF
39DAF
42DAF
R.G
T.S
R.G
27DAF
30DAF
33DAF
36DAF
39DAF
42DAF
27DAF
30DAF
33DAF
36DAF
39DAF
42DAF
27DAF
30DAF
33DAF
36DAF
39DAF
42DAF
T.S
K
F

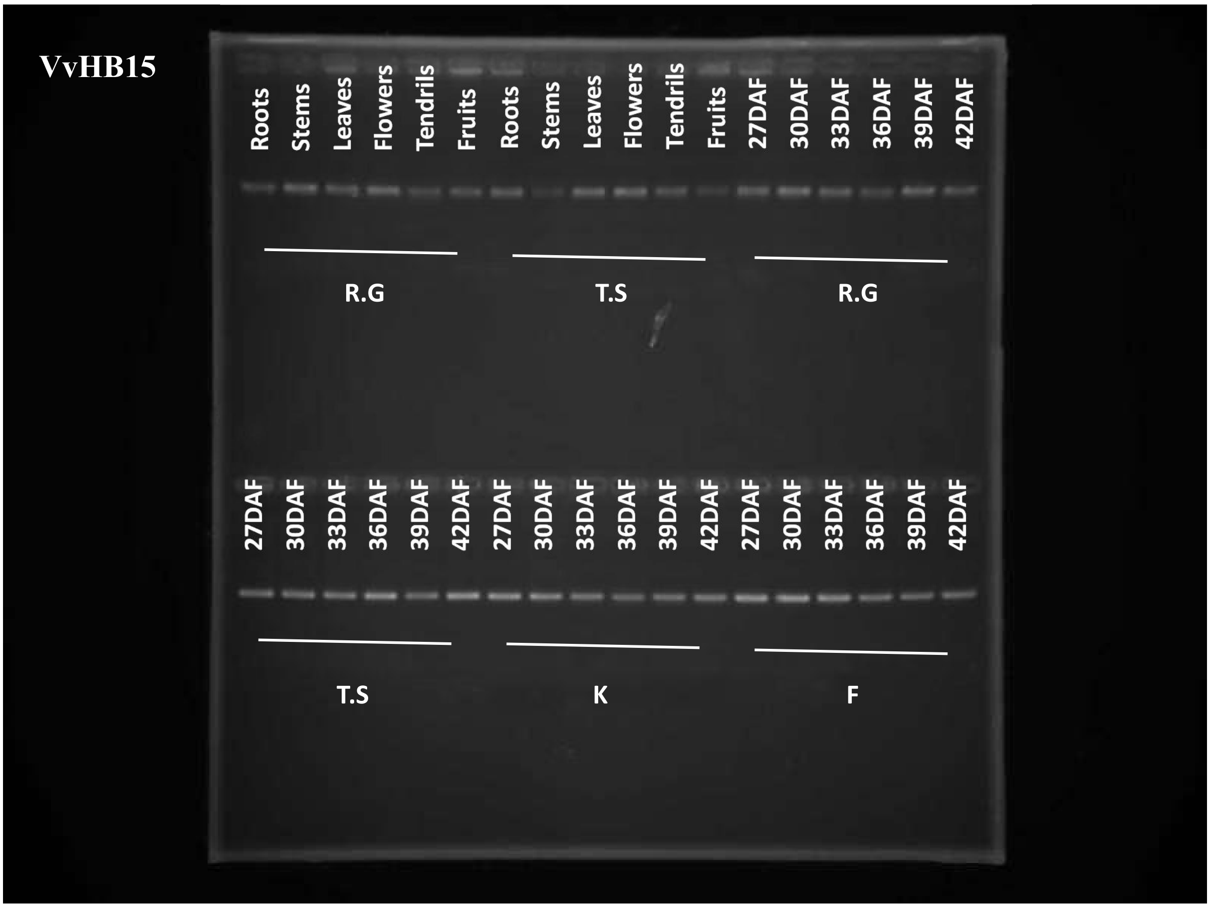
VvHB15
Roots
Stems
Leaves
Flowers
Tendrils
Fruits
Roots
Stems
Leaves
Flowers
Tendrils
Fruits
27DAF
30DAF
33DAF
36DAF
39DAF
42DAF
R.G
T.S
R.G
27DAF
30DAF
33DAF
36DAF
39DAF
42DAF
27DAF
30DAF
33DAF
36DAF
39DAF
42DAF
27DAF
30DAF
33DAF
36DAF
39DAF
42DAF
T.S
K
F

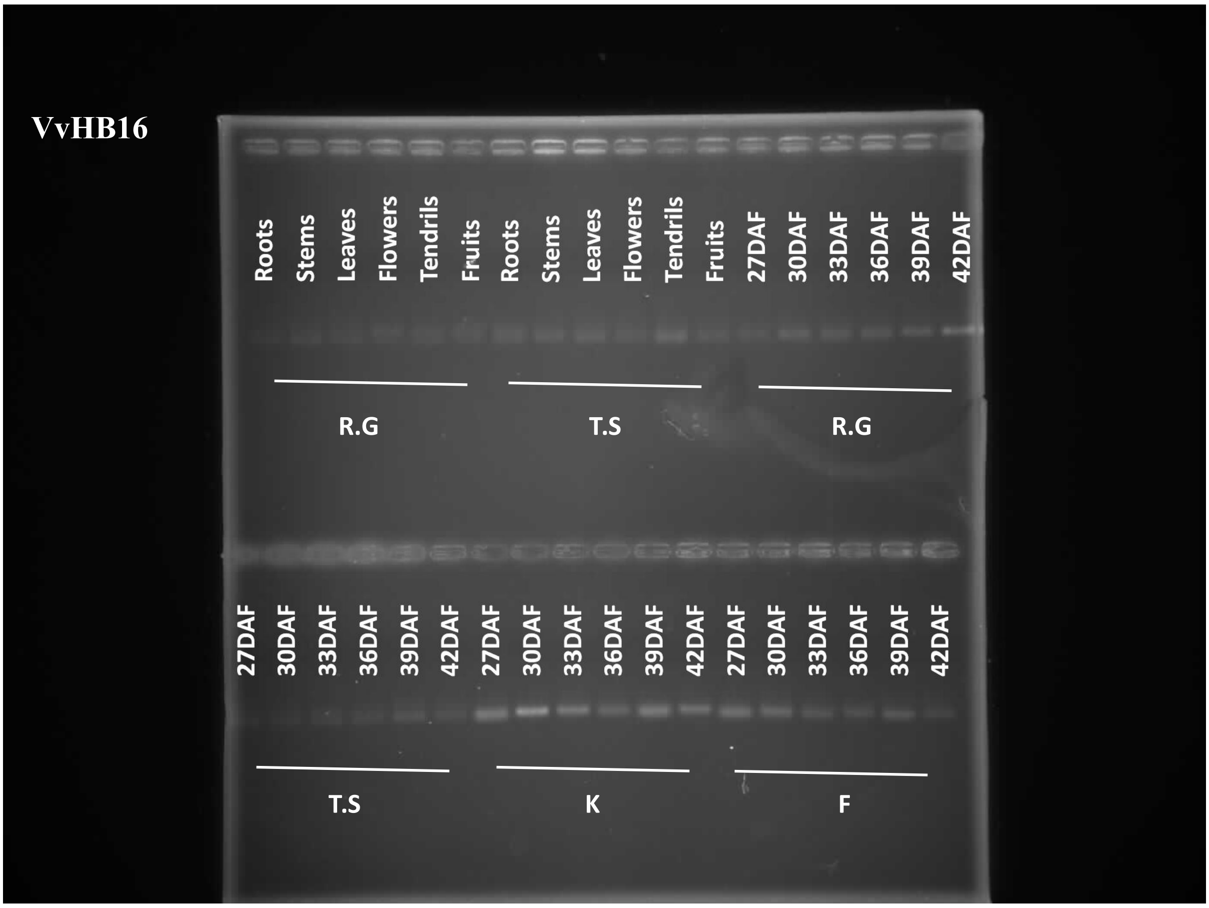
VvHB16
Roots
Stems
Leaves
Flowers
Tendrils
Fruits
Roots
Stems
Leaves
Flowers
Tendrils
Fruits
27DAF
30DAF
33DAF
36DAF
39DAF
42DAF
R.G
T.S
R.G
27DAF
30DAF
33DAF
36DAF
39DAF
42DAF
27DAF
30DAF
33DAF
36DAF
39DAF
42DAF
27DAF
30DAF
33DAF
36DAF
39DAF
42DAF
T.S
K
F

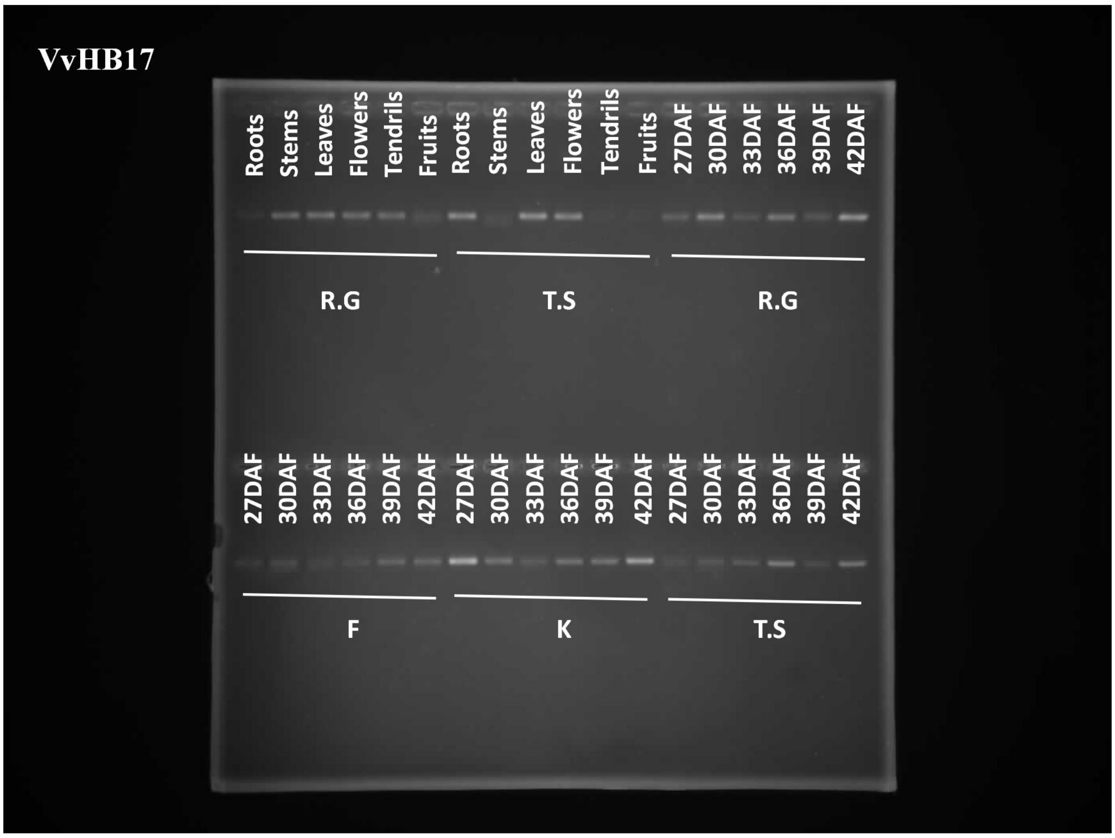
VvHB17
Roots
Stems
Leaves
Flowers
Tendrils
Fruits
Roots
Stems
Leaves
Flowers
Tendrils
Fruits
27DAF
30DAF
33DAF
36DAF
39DAF
42DAF
R.G
T.S
R.G
27DAF
30DAF
33DAF
36DAF
39DAF
42DAF
27DAF
30DAF
33DAF
36DAF
39DAF
42DAF
27DAF
30DAF
33DAF
36DAF
39DAF
42DAF
F
K
T.S

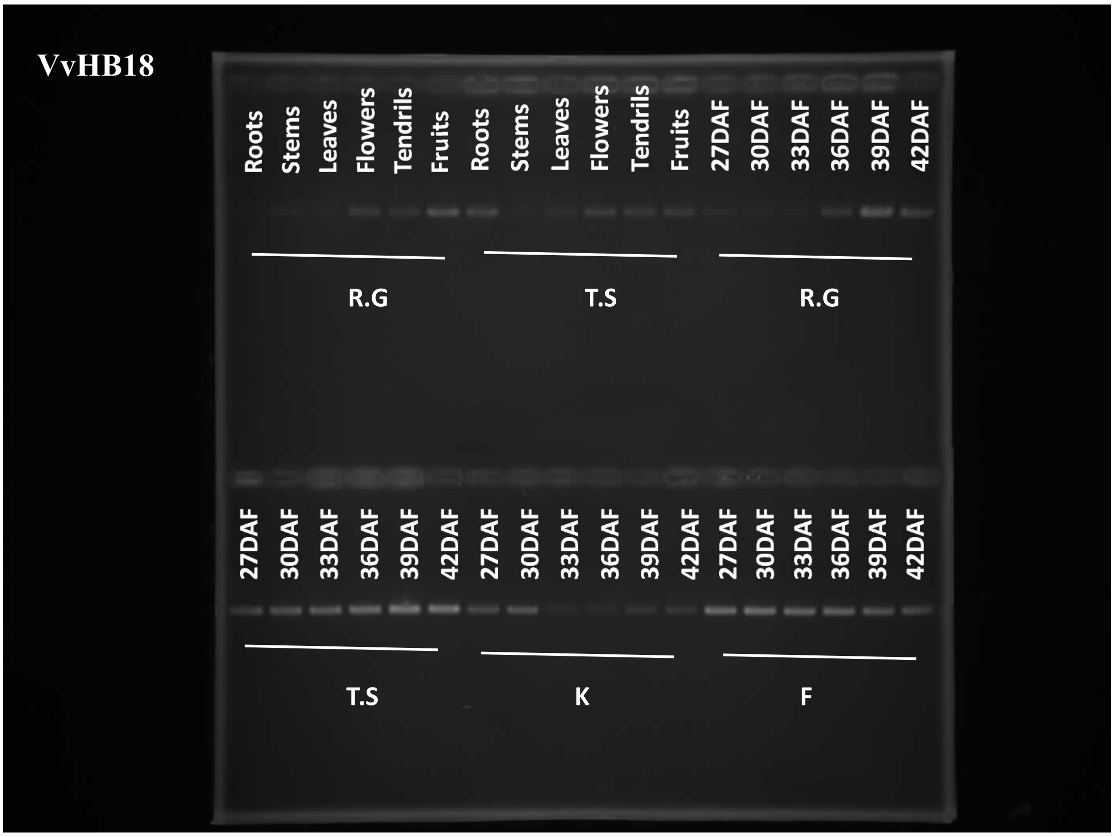
VvHB18
Roots
Stems
Leaves
Flowers
Tendrils
Fruits
Roots
Stems
Leaves
Flowers
Tendrils
Fruits
27DAF
30DAF
33DAF
36DAF
39DAF
42DAF
R.G
T.S
R.G
27DAF
30DAF
33DAF
36DAF
39DAF
42DAF
27DAF
30DAF
33DAF
36DAF
39DAF
42DAF
27DAF
30DAF
33DAF
36DAF
39DAF
42DAF
T.S
K
F

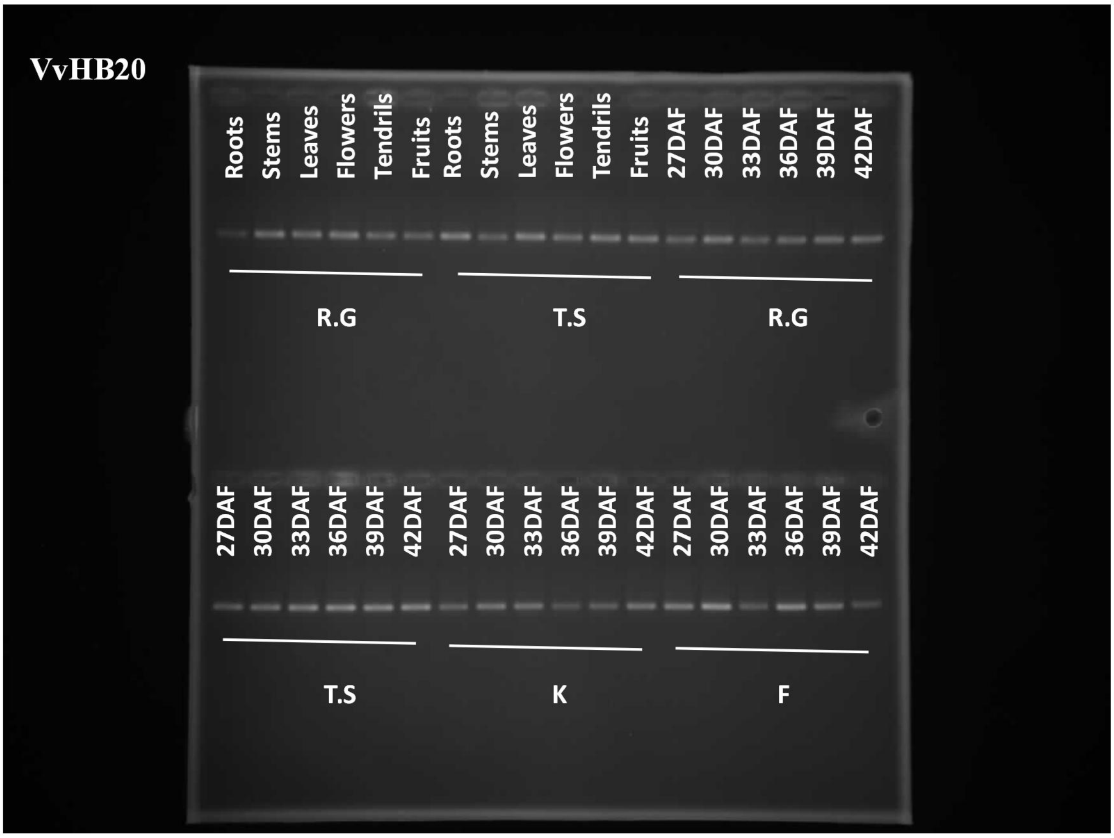
VvHB20
Roots
Stems
Leaves
Flowers
Tendrils
Fruits
Roots
Stems
Leaves
Flowers
Tendrils
Fruits
27DAF
30DAF
33DAF
36DAF
39DAF
42DAF
R.G
T.S
R.G
27DAF
30DAF
33DAF
36DAF
39DAF
42DAF
27DAF
30DAF
33DAF
36DAF
39DAF
42DAF
27DAF
30DAF
33DAF
36DAF
39DAF
42DAF
T.S
K
F

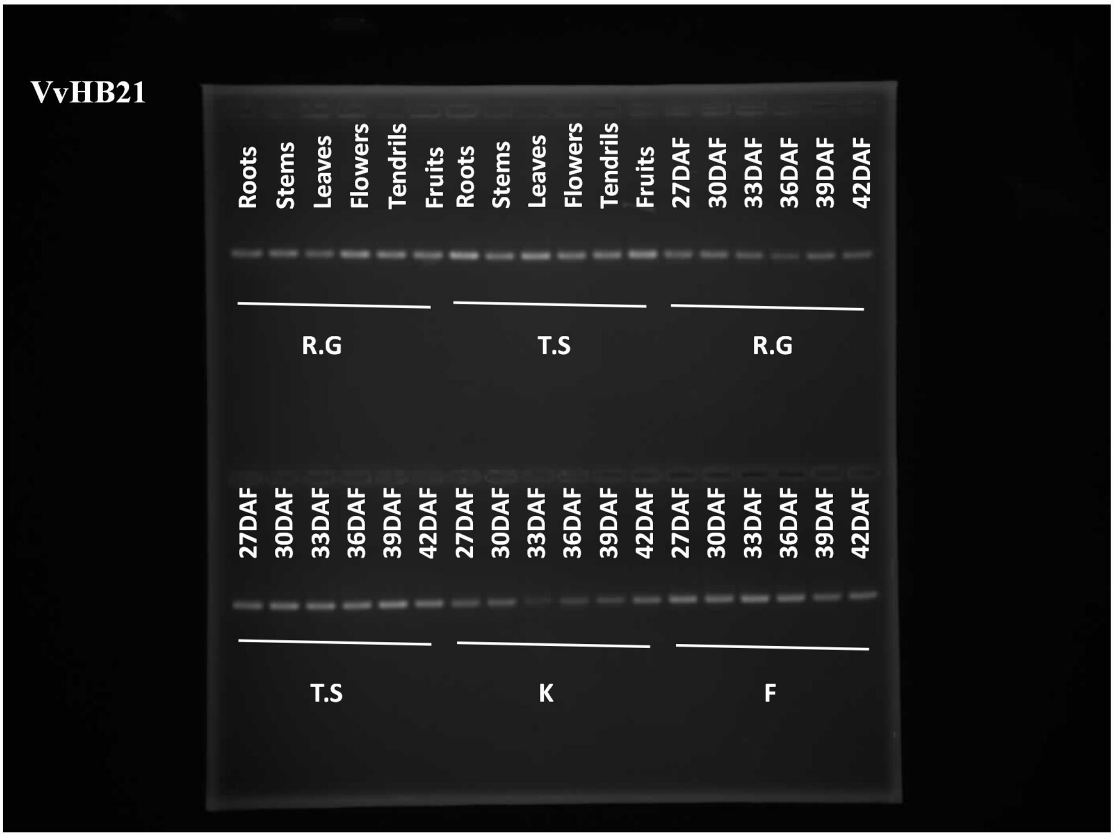
VvHB21
Roots
Stems
Leaves
Flowers
Tendrils
Fruits
Roots
Stems
Leaves
Flowers
Tendrils
Fruits
27DAF
30DAF
33DAF
36DAF
39DAF
42DAF
R.G
T.S
R.G
27DAF
30DAF
33DAF
36DAF
39DAF
42DAF
27DAF
30DAF
33DAF
36DAF
39DAF
42DAF
27DAF
30DAF
33DAF
36DAF
39DAF
42DAF
T.S
K
F

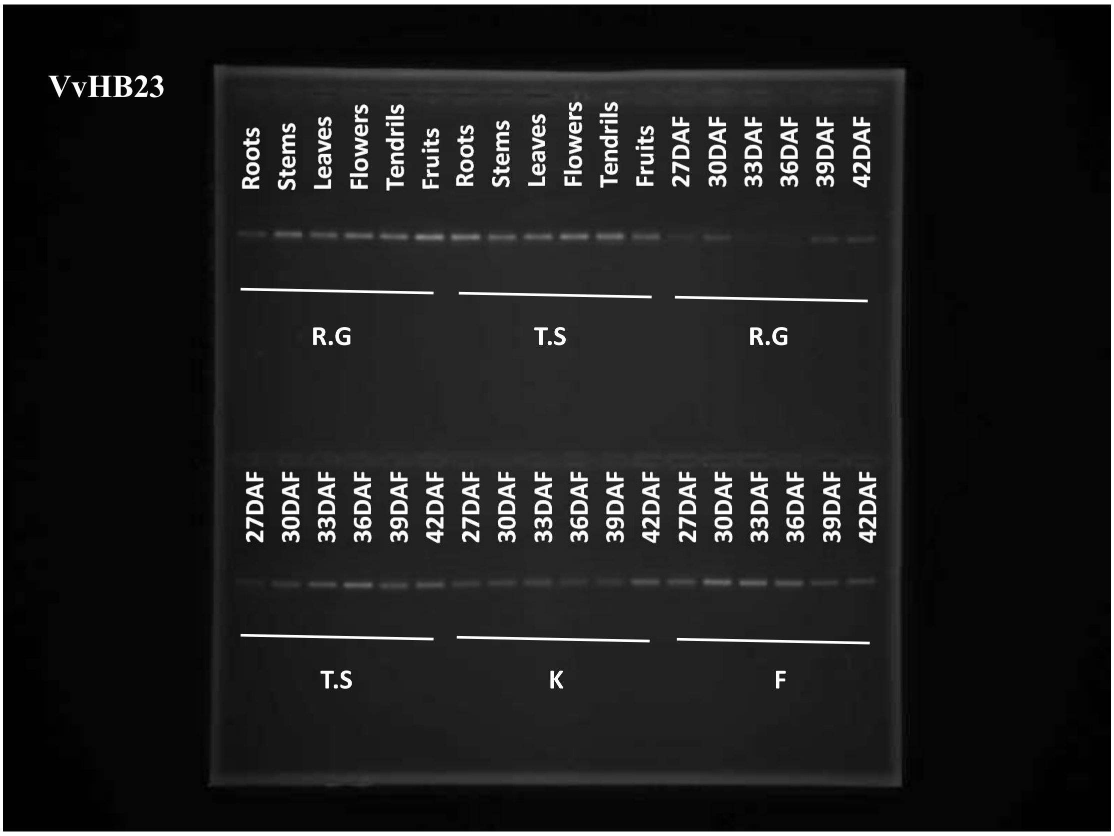
VvHB23
Roots Stems Leaves Flowers Tendrils Fruits Roots Stems Leaves Flowers Tendrils Fruits 27DAF 30DAF 33DAF 36DAF 39DAF 42DAF
R.G
T.S
R.G
27DAF 30DAF 33DAF 36DAF 39DAF 42DAF 27DAF 30DAF 33DAF 36DAF 39DAF 42DAF 27DAF 30DAF 33DAF 36DAF 39DAF 42DAF
T.S
K
F

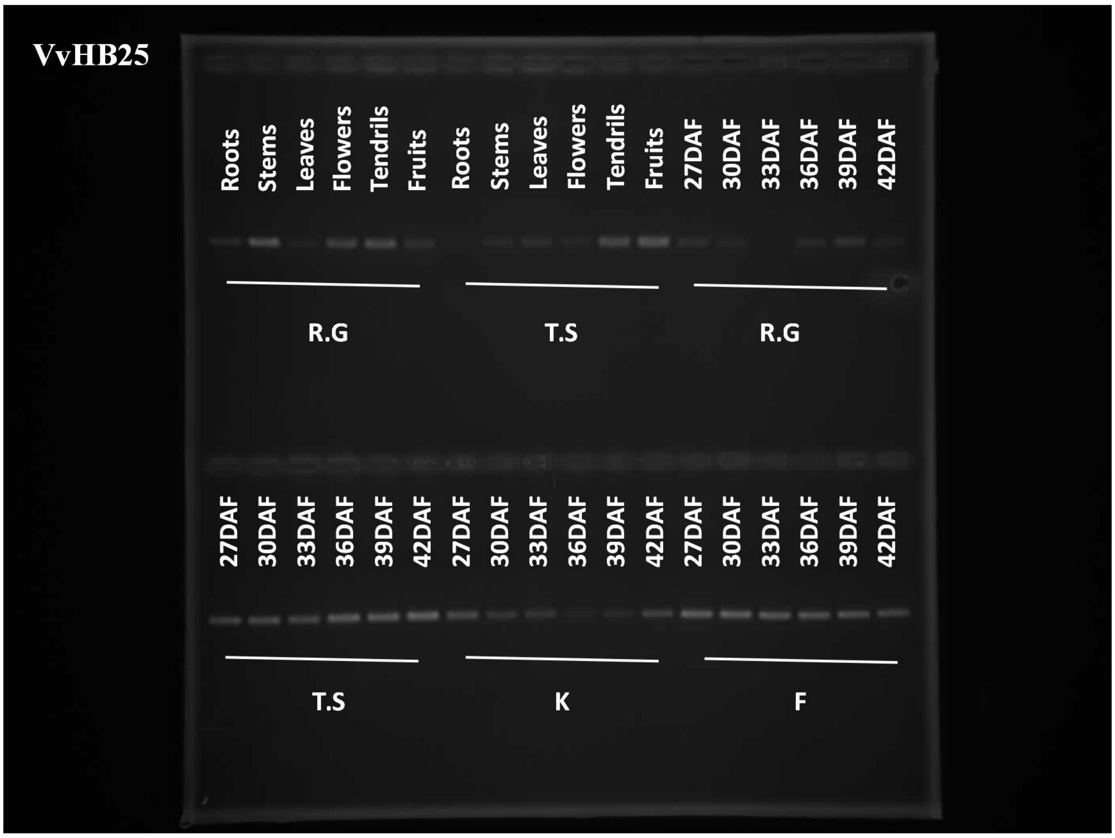
VvHB25
Roots Stems Leaves Flowers Tendrils Fruits Roots Stems Leaves Flowers Tendrils Fruits 27DAF 30DAF 33DAF 36DAF 39DAF 42DAF
R.G
T.S
R.G
27DAF 30DAF 33DAF 36DAF 39DAF 42DAF 27DAF 30DAF 33DAF 36DAF 39DAF 42DAF 27DAF 30DAF 33DAF 36DAF 39DAF 42DAF
T.S
K
F

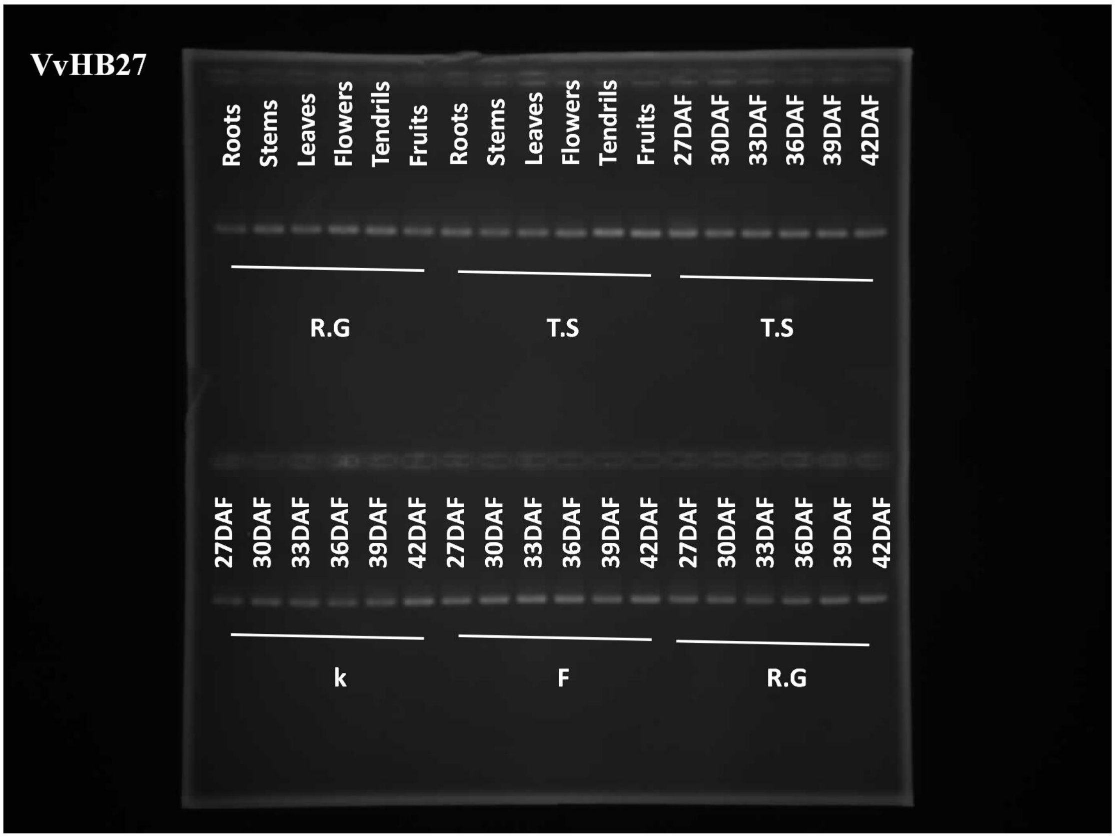
VvHB27
Roots Stems Leaves Flowers Tendrils Fruits Roots Stems Leaves Flowers Tendrils Fruits 27DAF 30DAF 33DAF 36DAF 39DAF 42DAF
R.G
T.S
T.S
27DAF 30DAF 33DAF 36DAF 39DAF 42DAF 27DAF 30DAF 33DAF 36DAF 39DAF 42DAF 27DAF 30DAF 33DAF 36DAF 39DAF 42DAF
k
F
R.G

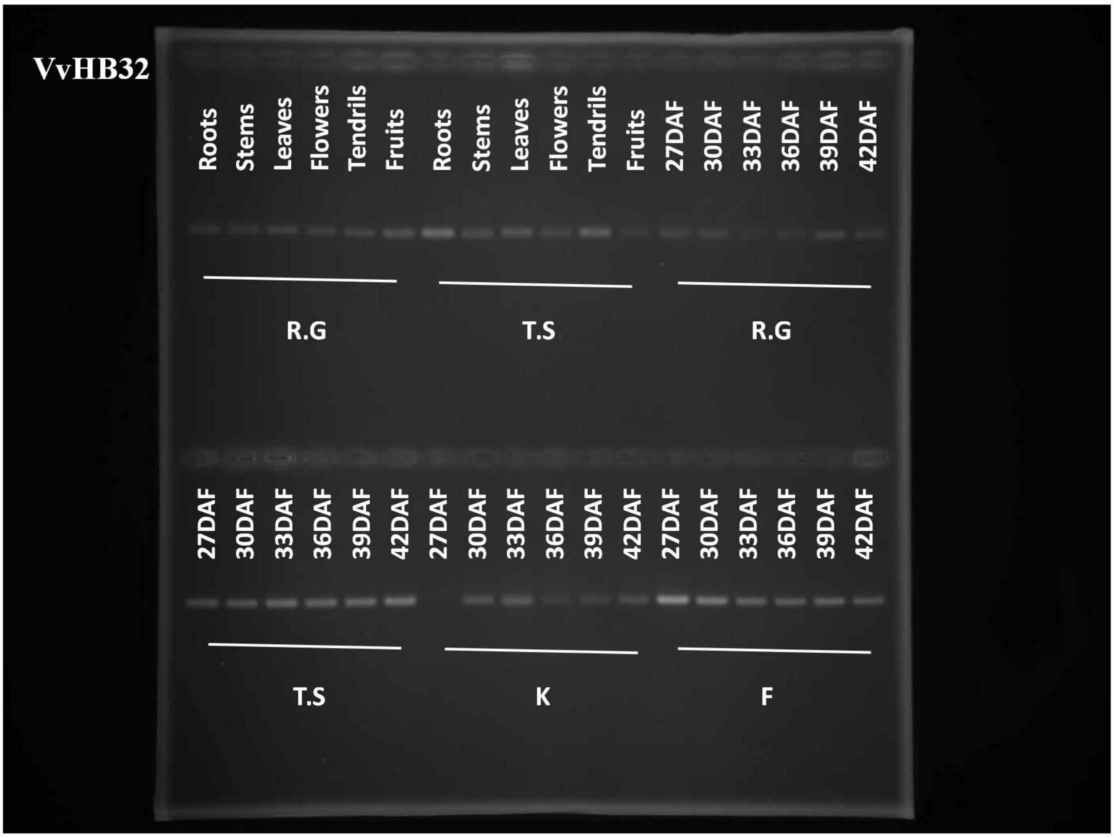
VvHB32
Roots Stems Leaves Flowers Tendrils Fruits Roots Stems Leaves Flowers Tendrils Fruits 27DAF 30DAF 33DAF 36DAF 39DAF 42DAF
R.G
T.S
R.G
27DAF 30DAF 33DAF 36DAF 39DAF 42DAF 27DAF 30DAF 33DAF 36DAF 39DAF 42DAF 27DAF 30DAF 33DAF 36DAF 39DAF 42DAF
T.S
K
F

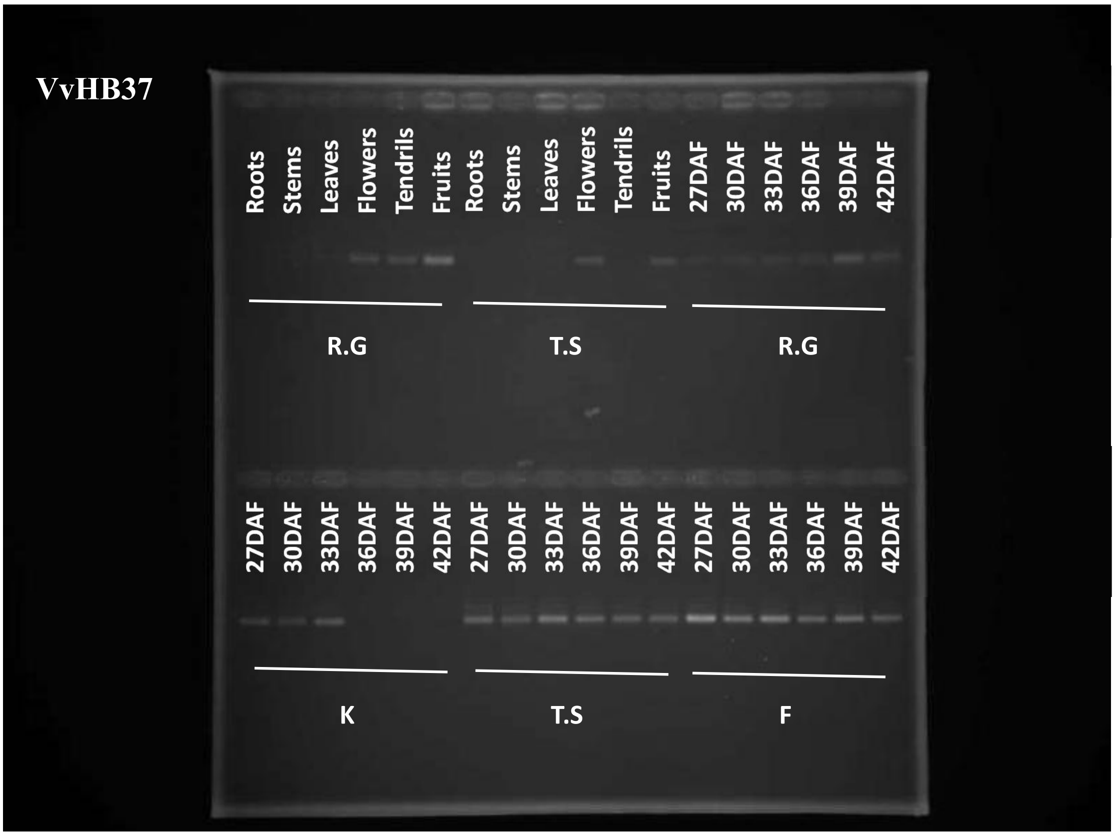
VvHB37
Roots
Stems
Leaves
Flowers
Tendrils
Fruits
Roots
Stems
Leaves
Flowers
Tendrils
Fruits
27DAF
30DAF
33DAF
36DAF
39DAF
42DAF
R.G
T.S
R.G
27DAF
30DAF
33DAF
36DAF
39DAF
42DAF
27DAF
30DAF
33DAF
36DAF
39DAF
42DAF
27DAF
30DAF
33DAF
36DAF
39DAF
42DAF
K
T.S
F

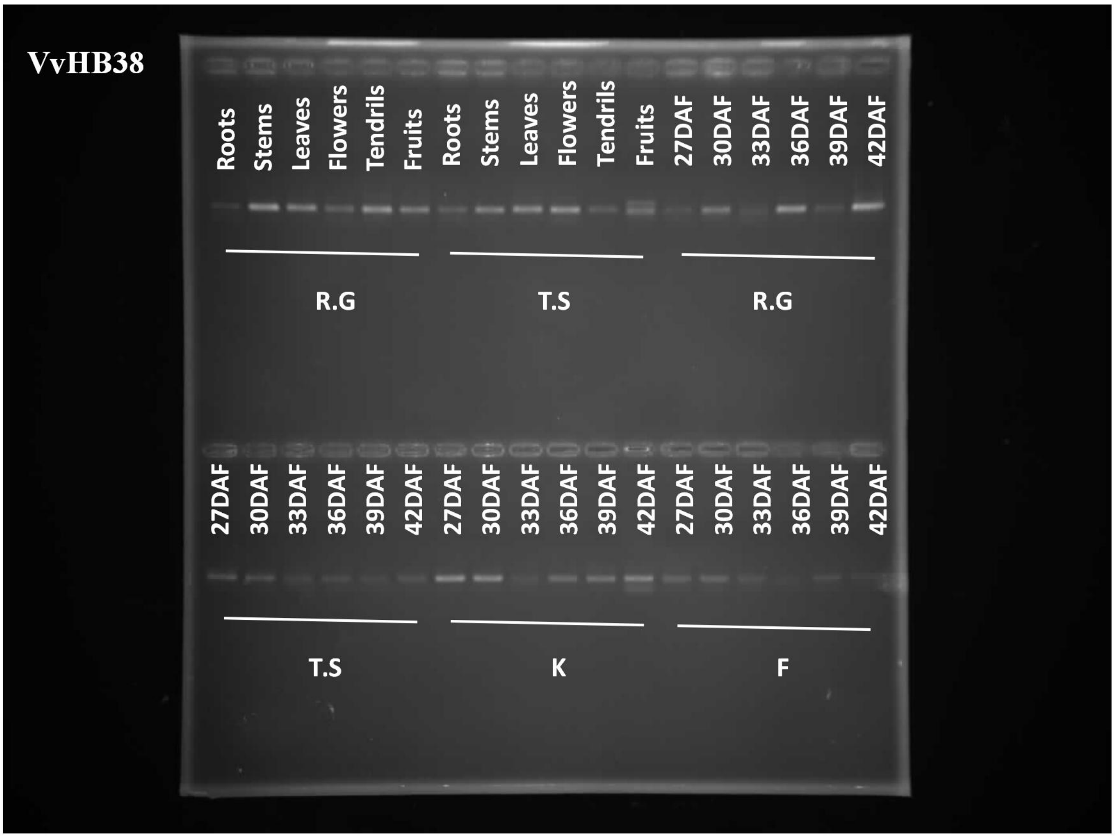
VvHB38
Roots
Stems
Leaves
Flowers
Tendrils
Fruits
Roots
Stems
Leaves
Flowers
Tendrils
Fruits
27DAF
30DAF
33DAF
36DAF
39DAF
42DAF
R.G
T.S
R.G
27DAF
30DAF
33DAF
36DAF
39DAF
42DAF
27DAF
30DAF
33DAF
36DAF
39DAF
42DAF
27DAF
30DAF
33DAF
36DAF
39DAF
42DAF
T.S
K
F

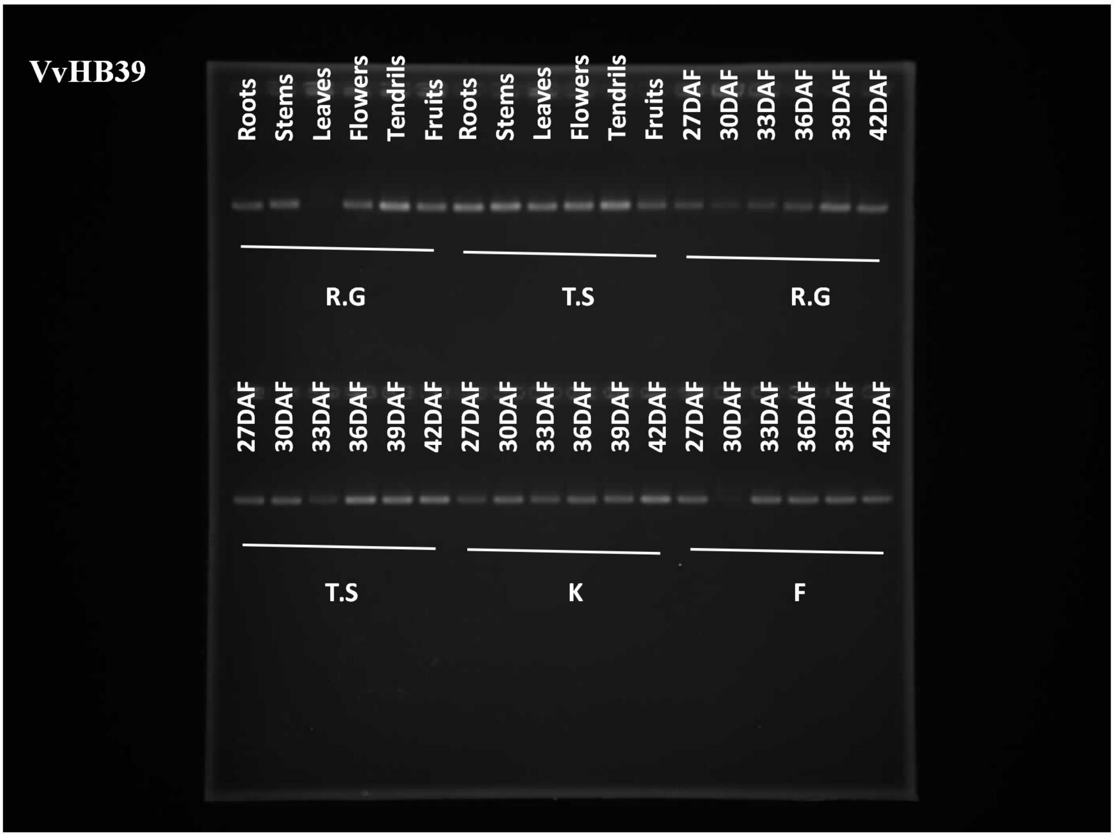
VvHB39
Roots
Stems
Leaves
Flowers
Tendrils
Fruits
Roots
Stems
Leaves
Flowers
Tendrils
Fruits
27DAF
30DAF
33DAF
36DAF
39DAF
42DAF
R.G
T.S
R.G
27DAF
30DAF
33DAF
36DAF
39DAF
42DAF
27DAF
30DAF
33DAF
36DAF
39DAF
42DAF
27DAF
30DAF
33DAF
36DAF
39DAF
42DAF
T.S
K
F

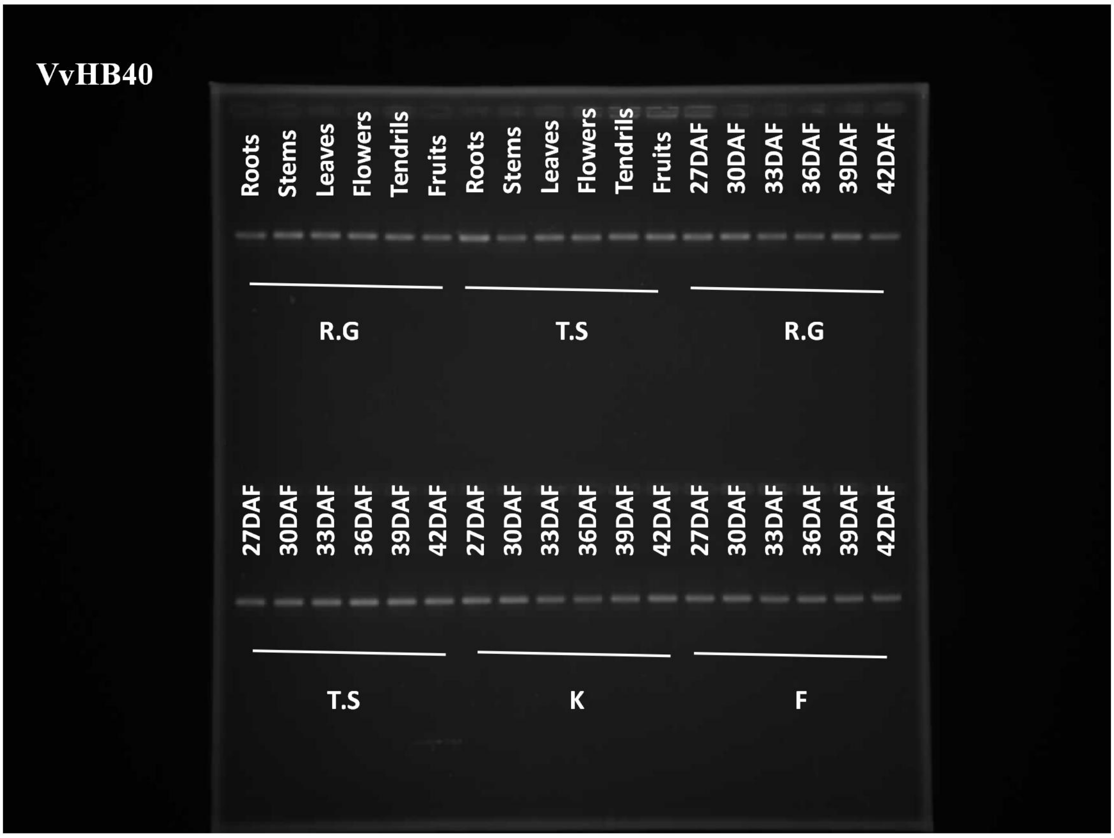
VvHB40
Roots
Stems
Leaves
Flowers
Tendrils
Fruits
Roots
Stems
Leaves
Flowers
Tendrils
Fruits
27DAF
30DAF
33DAF
36DAF
39DAF
42DAF
R.G
T.S
R.G
27DAF
30DAF
33DAF
36DAF
39DAF
42DAF
27DAF
30DAF
33DAF
36DAF
39DAF
42DAF
27DAF
30DAF
33DAF
36DAF
39DAF
42DAF
T.S
K
F

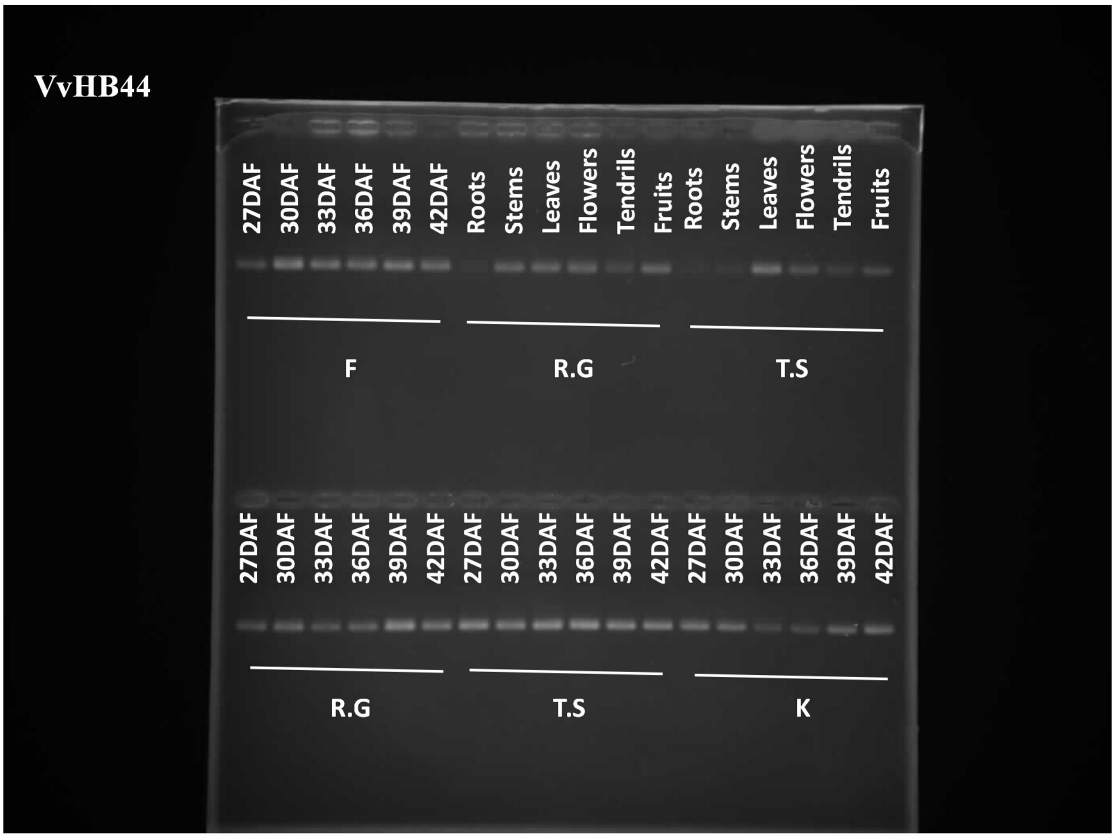
VvHB44
27DAF
30DAF
33DAF
36DAF
39DAF
42DAF
Roots
Stems
Leaves
Flowers
Tendrils
Fruits
Roots
Stems
Leaves
Flowers
Tendrils
Fruits
F
R.G
T.S
27DAF
30DAF
33DAF
36DAF
39DAF
42DAF
27DAF
30DAF
33DAF
36DAF
39DAF
42DAF
27DAF
30DAF
33DAF
36DAF
39DAF
42DAF
R.G
T.S
K

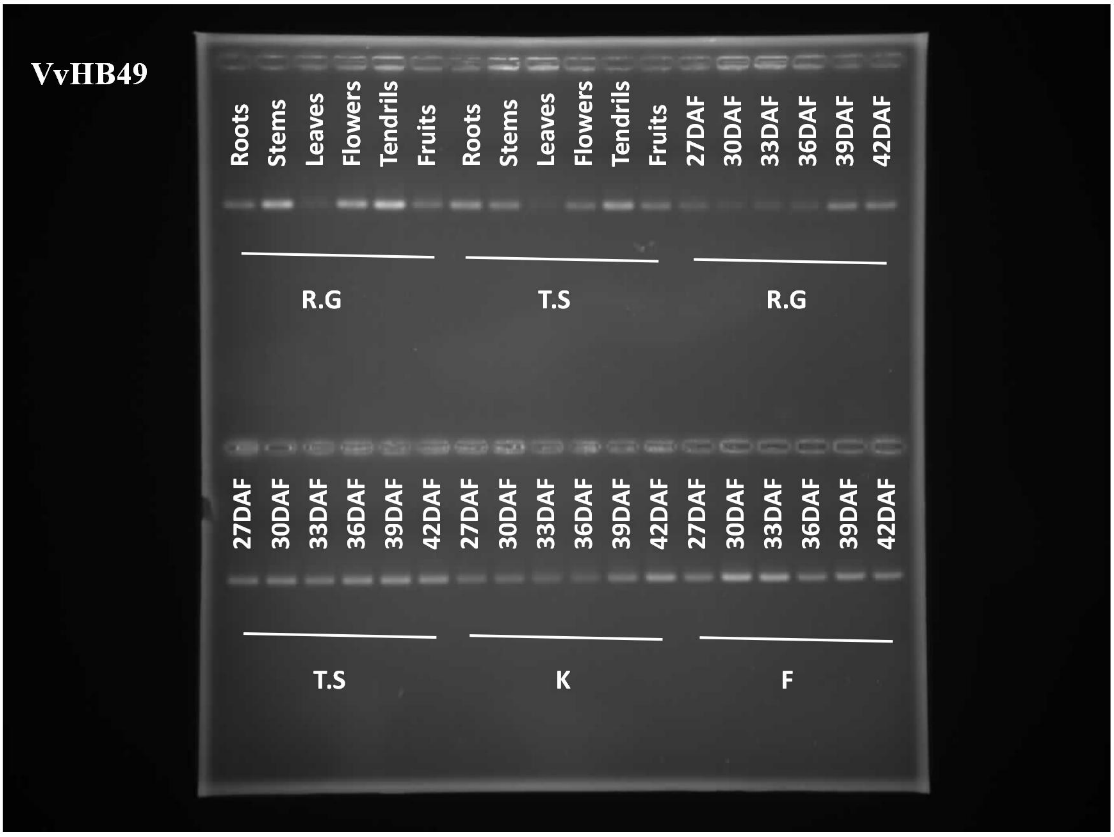
VvHB49
Roots
Stems
Leaves
Flowers
Tendrils
Fruits
Roots
Stems
Leaves
Flowers
Tendrils
Fruits
27DAF
30DAF
33DAF
36DAF
39DAF
42DAF
R.G
T.S
R.G
27DAF
30DAF
33DAF
36DAF
39DAF
42DAF
27DAF
30DAF
33DAF
36DAF
39DAF
42DAF
27DAF
30DAF
33DAF
36DAF
39DAF
42DAF
T.S
K
F

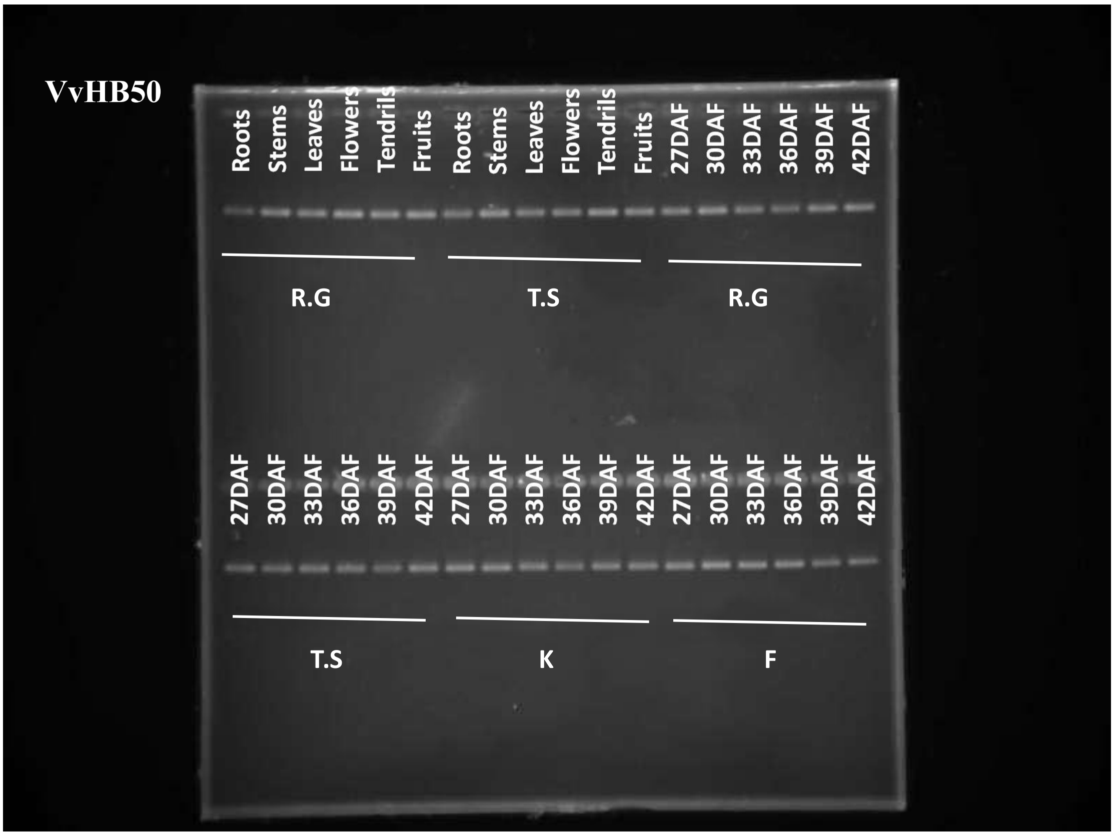
VvHB50
Roots
Stems
Leaves
Flowers
Tendrils
Fruits
Roots
Stems
Leaves
Flowers
Tendrils
Fruits
27DAF
30DAF
33DAF
36DAF
39DAF
42DAF
R.G
T.S
R.G
27DAF
30DAF
33DAF
36DAF
39DAF
42DAF
27DAF
30DAF
33DAF
36DAF
39DAF
42DAF
27DAF
30DAF
33DAF
36DAF
39DAF
42DAF
T.S
K
F

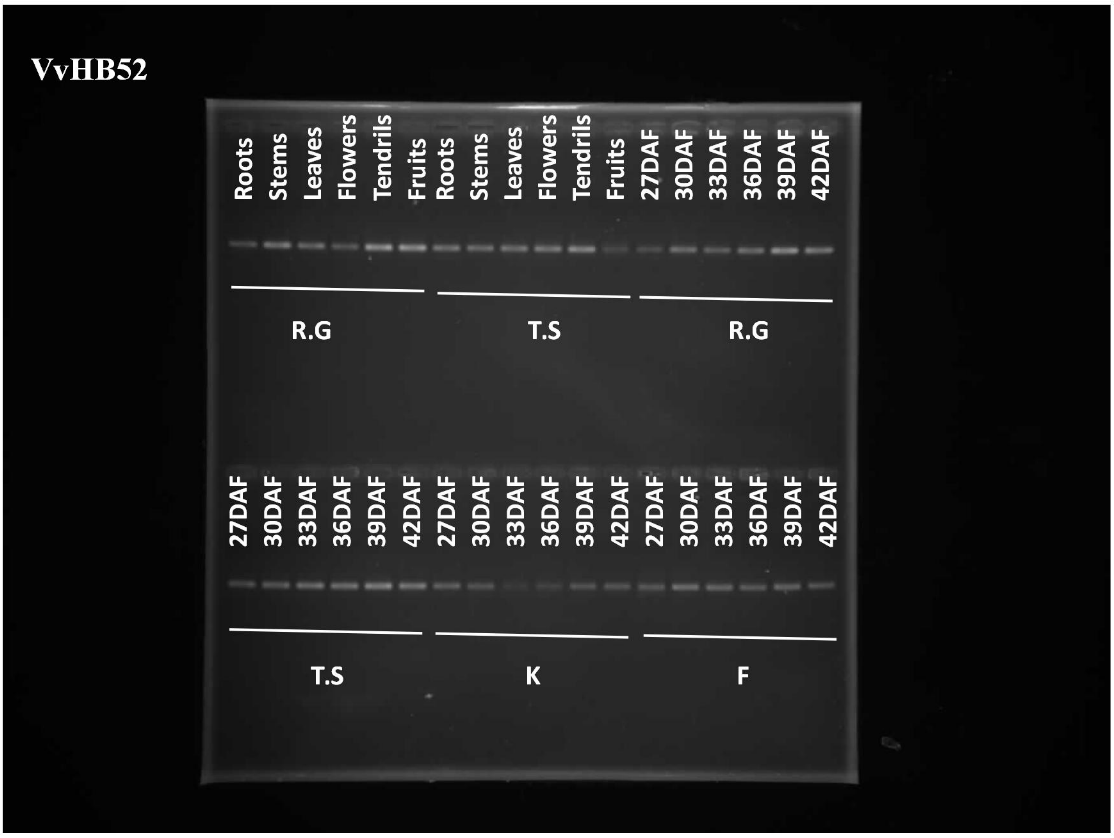
VvHB52
Roots
Stems
Leaves
Flowers
Tendrils
Fruits
Roots
Stems
Leaves
Flowers
Tendrils
Fruits
27DAF
30DAF
33DAF
36DAF
39DAF
42DAF
R.G
T.S
R.G
27DAF
30DAF
33DAF
36DAF
39DAF
42DAF
27DAF
30DAF
33DAF
36DAF
39DAF
42DAF
27DAF
30DAF
33DAF
36DAF
39DAF
42DAF
T.S
K
F

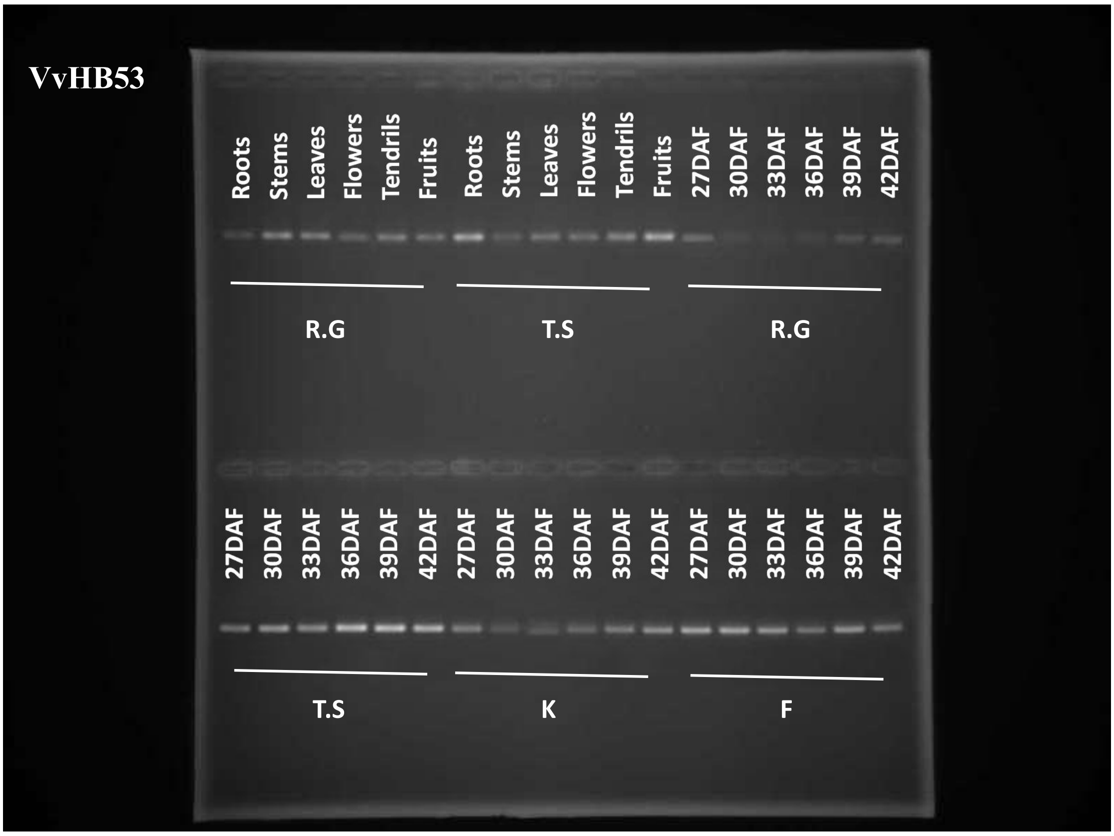
VvHB53
Roots
Stems
Leaves
Flowers
Tendrils
Fruits
Roots
Stems
Leaves
Flowers
Tendrils
Fruits
27DAF
30DAF
33DAF
36DAF
39DAF
42DAF
R.G
T.S
R.G
27DAF
30DAF
33DAF
36DAF
39DAF
42DAF
27DAF
30DAF
33DAF
36DAF
39DAF
42DAF
27DAF
30DAF
33DAF
36DAF
39DAF
42DAF
T.S
K
F

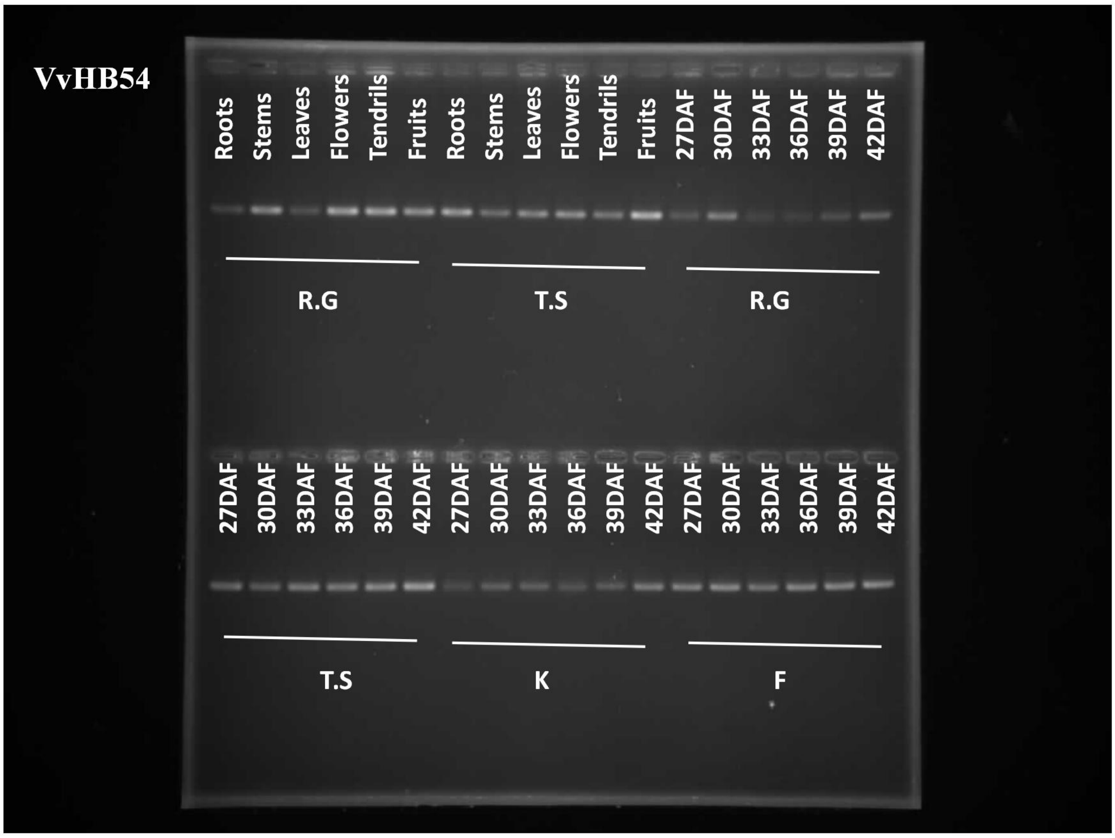
VvHB54
Roots
Stems
Leaves
Flowers
Tendrils
Fruits
Roots
Stems
Leaves
Flowers
Tendrils
Fruits
27DAF
30DAF
33DAF
36DAF
39DAF
42DAF
R.G
T.S
R.G
27DAF
30DAF
33DAF
36DAF
39DAF
42DAF
27DAF
30DAF
33DAF
36DAF
39DAF
42DAF
27DAF
30DAF
33DAF
36DAF
39DAF
42DAF
T.S
K
F

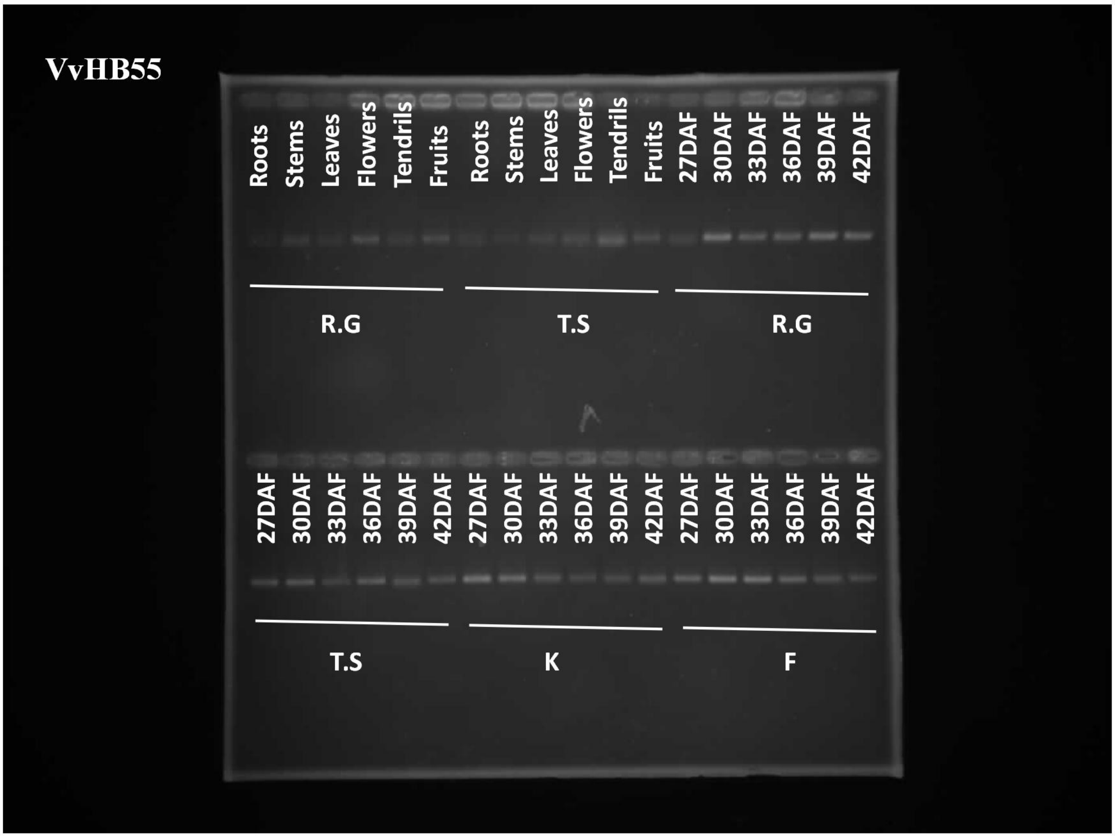
VvHB55
Roots
Stems
Leaves
Flowers
Tendrils
Fruits
Roots
Stems
Leaves
Flowers
Tendrils
Fruits
27DAF
30DAF
33DAF
36DAF
39DAF
42DAF
R.G
T.S
R.G
27DAF
30DAF
33DAF
36DAF
39DAF
42DAF
27DAF
30DAF
33DAF
36DAF
39DAF
42DAF
27DAF
30DAF
33DAF
36DAF
39DAF
42DAF
T.S
K
F

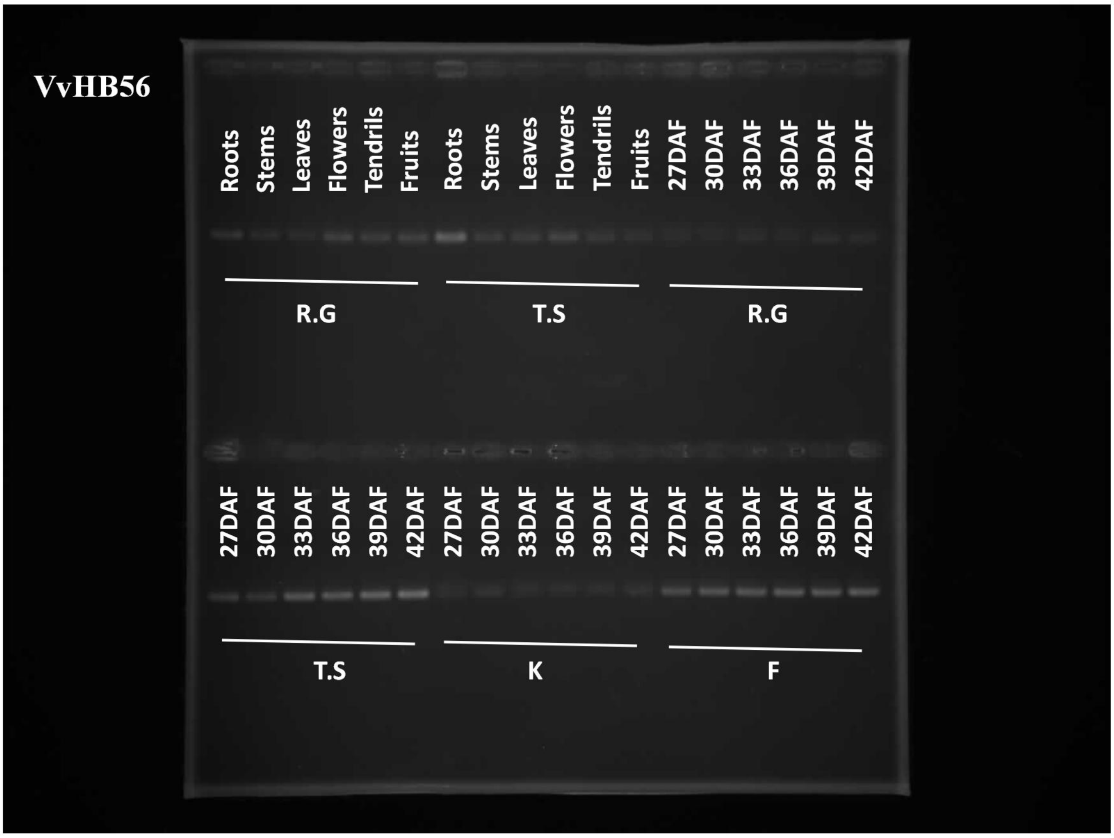
VvHB56
Roots
Stems
Leaves
Flowers
Tendrils
Fruits
Roots
Stems
Leaves
Flowers
Tendrils
Fruits
27DAF
30DAF
33DAF
36DAF
39DAF
42DAF
R.G
T.S
R.G
27DAF
30DAF
33DAF
36DAF
39DAF
42DAF
27DAF
30DAF
33DAF
36DAF
39DAF
42DAF
27DAF
30DAF
33DAF
36DAF
39DAF
42DAF
T.S
K
F

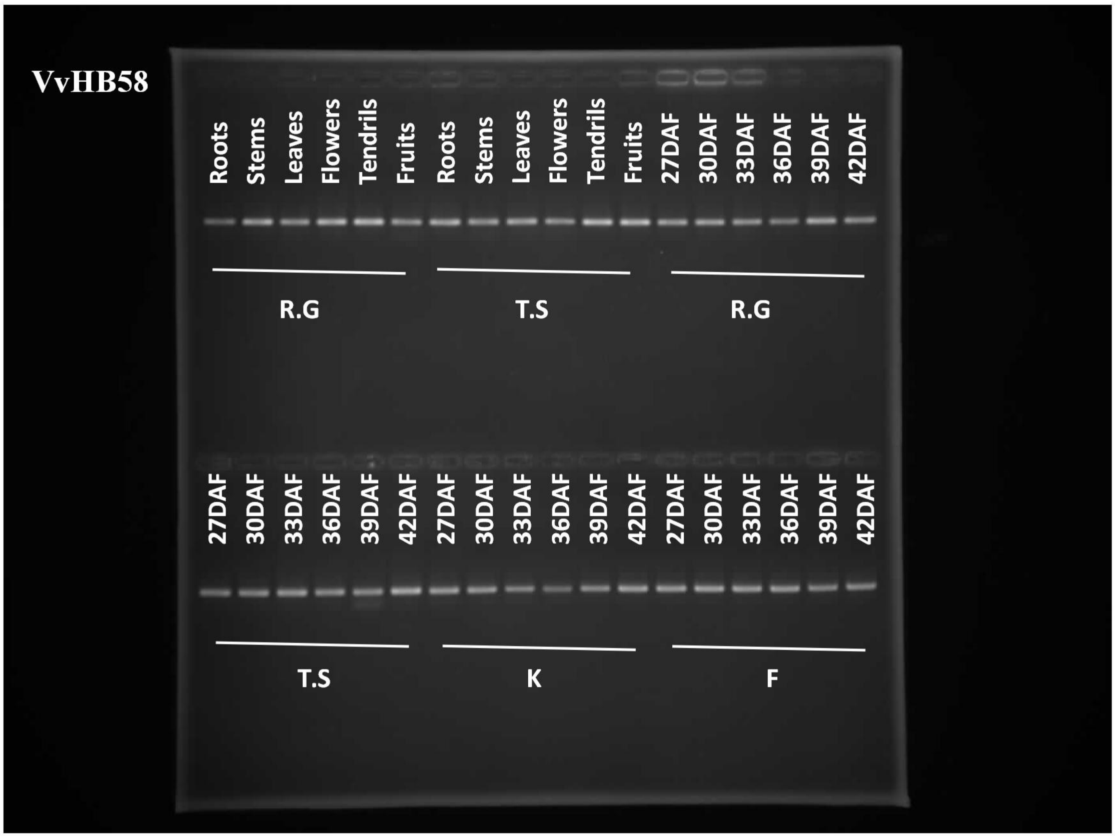
VvHB58
Roots
Stems
Leaves
Flowers
Tendrils
Fruits
Roots
Stems
Leaves
Flowers
Tendrils
Fruits
27DAF
30DAF
33DAF
36DAF
39DAF
42DAF
R.G
T.S
R.G
27DAF
30DAF
33DAF
36DAF
39DAF
42DAF
27DAF
30DAF
33DAF
36DAF
39DAF
42DAF
27DAF
30DAF
33DAF
36DAF
39DAF
42DAF
T.S
K
F

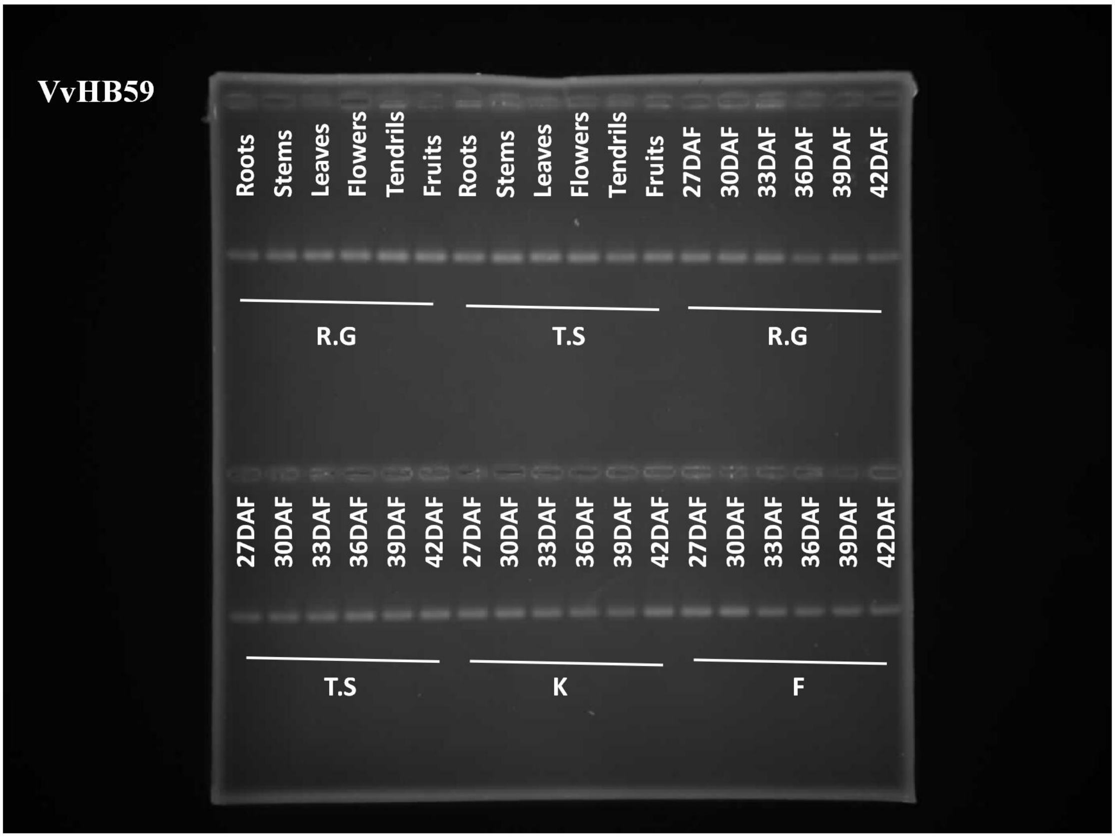
VvHB59
Roots
Stems
Leaves
Flowers
Tendrils
Fruits
Roots
Stems
Leaves
Flowers
Tendrils
Fruits
27DAF
30DAF
33DAF
36DAF
39DAF
42DAF
R.G
T.S
R.G
27DAF
30DAF
33DAF
36DAF
39DAF
42DAF
27DAF
30DAF
33DAF
36DAF
39DAF
42DAF
27DAF
30DAF
33DAF
36DAF
39DAF
42DAF
T.S
K
F

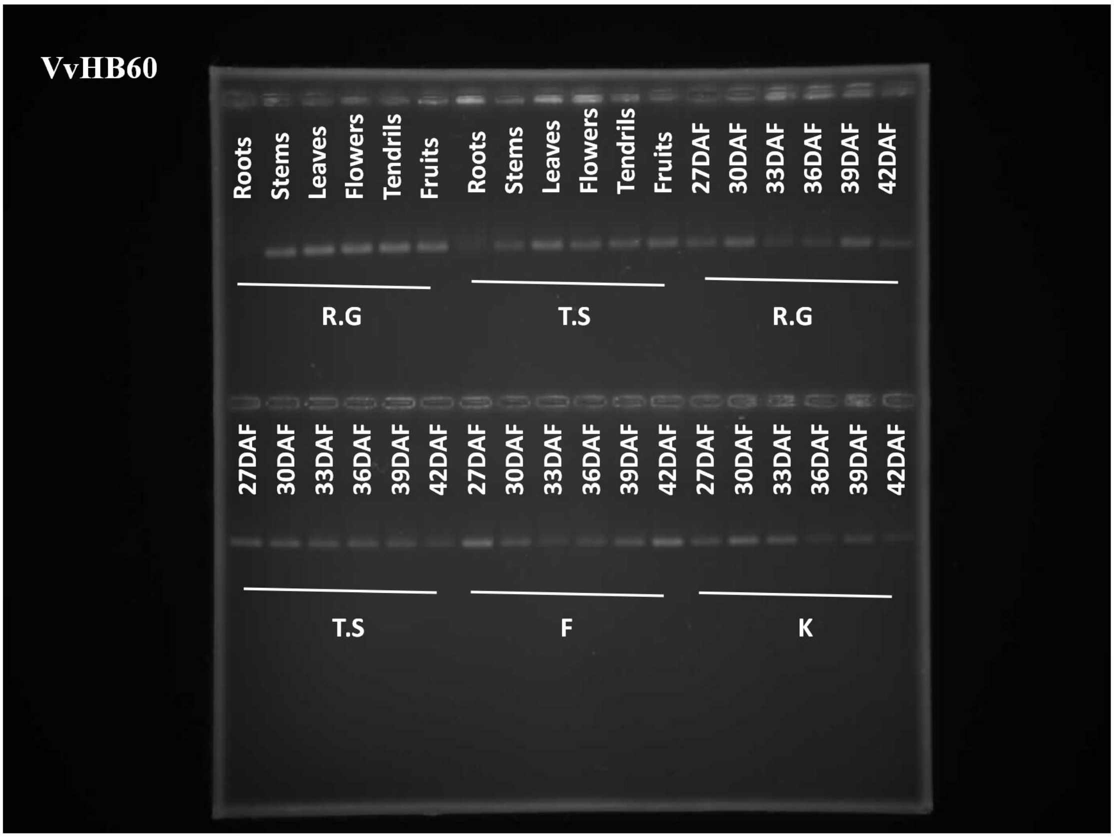
VvHB60
Roots
Stems
Leaves
Flowers
Tendrils
Fruits
Roots
Stems
Leaves
Flowers
Tendrils
Fruits
27DAF
30DAF
33DAF
36DAF
39DAF
42DAF
R.G
T.S
R.G
27DAF
30DAF
33DAF
36DAF
39DAF
42DAF
27DAF
30DAF
33DAF
36DAF
39DAF
42DAF
27DAF
30DAF
33DAF
36DAF
39DAF
42DAF
T.S
F
K

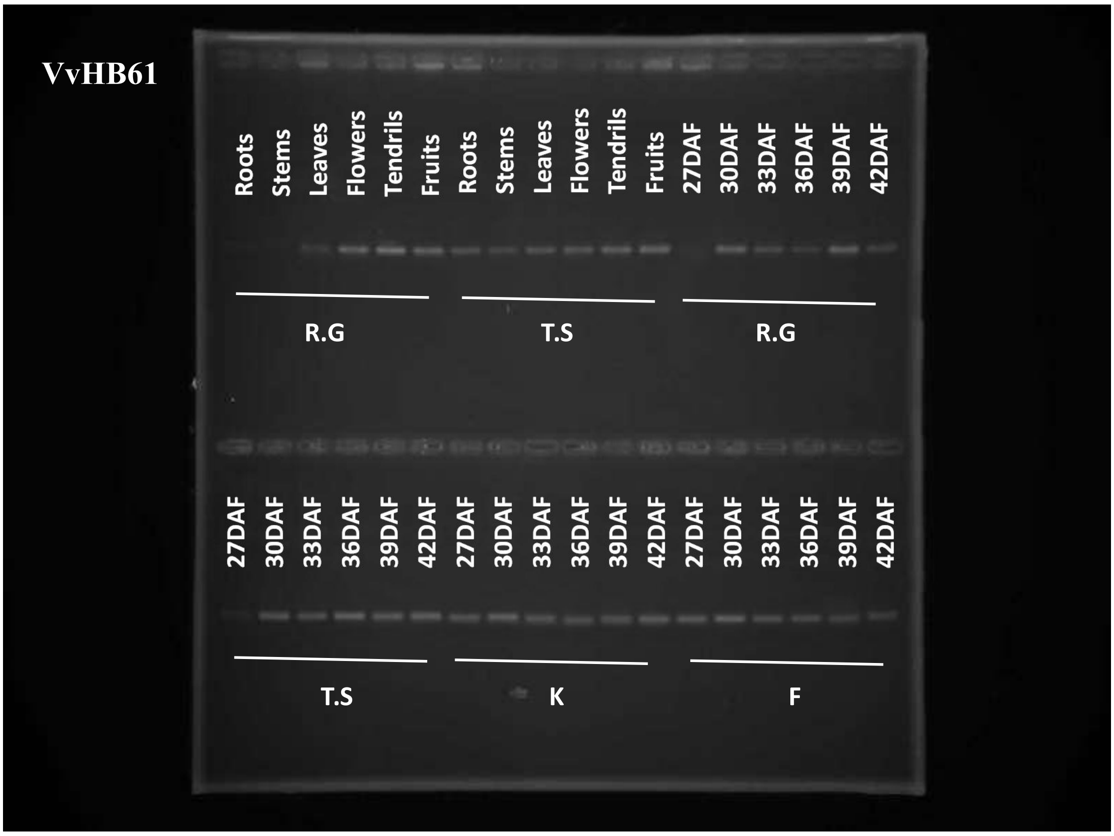
VvHB61
Roots
Stems
Leaves
Flowers
Tendrils
Fruits
Roots
Stems
Leaves
Flowers
Tendrils
Fruits
27DAF
30DAF
33DAF
36DAF
39DAF
42DAF
R.G
T.S
R.G
27DAF
30DAF
33DAF
36DAF
39DAF
42DAF
27DAF
30DAF
33DAF
36DAF
39DAF
42DAF
27DAF
30DAF
33DAF
36DAF
39DAF
42DAF
T.S
K
F

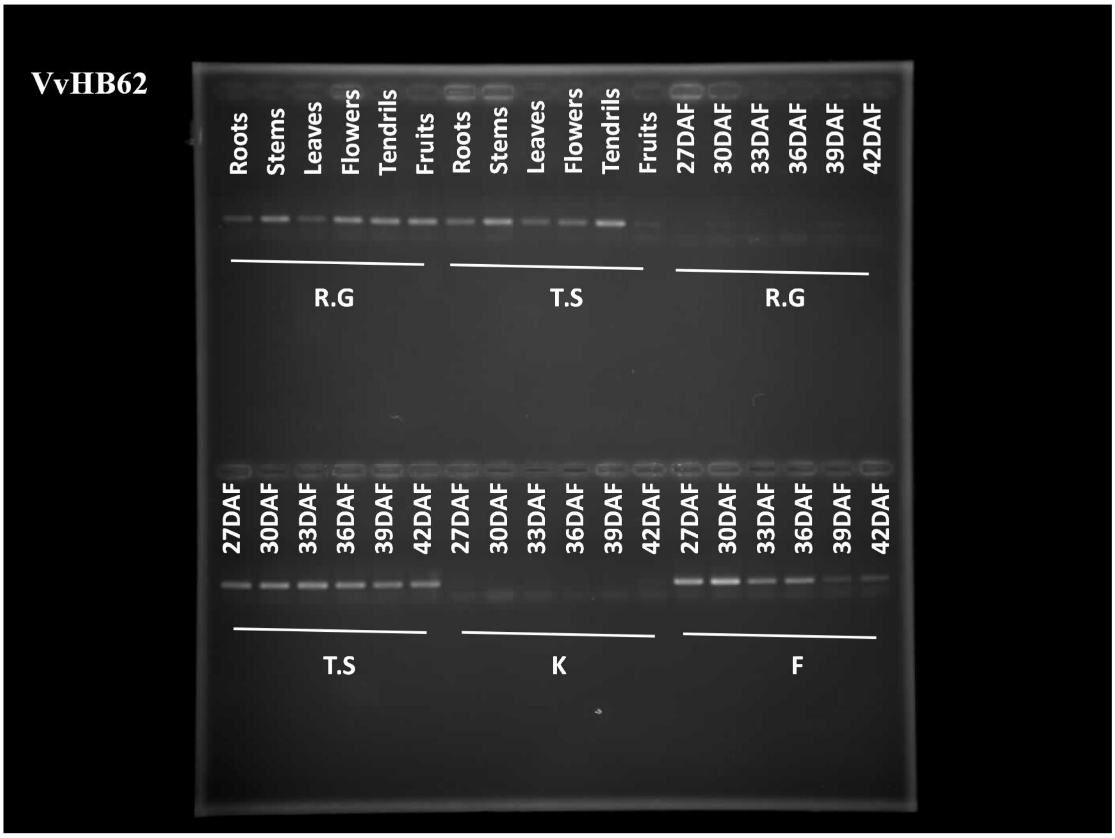
VvHB62
Roots
Stems
Leaves
Flowers
Tendrils
Fruits
Roots
Stems
Leaves
Flowers
Tendrils
Fruits
27DAF
30DAF
33DAF
36DAF
39DAF
42DAF
R.G
T.S
R.G
27DAF
30DAF
33DAF
36DAF
39DAF
42DAF
27DAF
30DAF
33DAF
36DAF
39DAF
42DAF
27DAF
30DAF
33DAF
36DAF
39DAF
42DAF
T.S
K
F

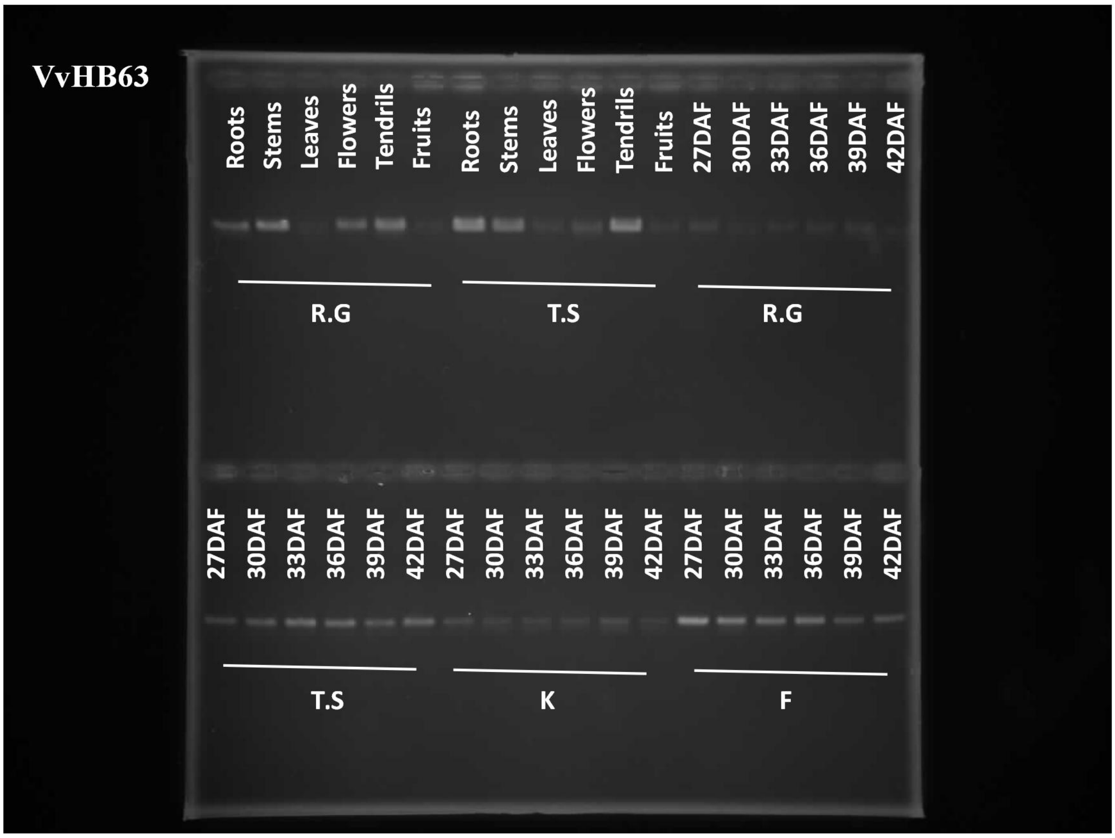
VvHB63
Roots
Stems
Leaves
Flowers
Tendrils
Fruits
Roots
Stems
Leaves
Flowers
Tendrils
Fruits
27DAF
30DAF
33DAF
36DAF
39DAF
42DAF
R.G
T.S
R.G
27DAF
30DAF
33DAF
36DAF
39DAF
42DAF
27DAF
30DAF
33DAF
36DAF
39DAF
42DAF
27DAF
30DAF
33DAF
36DAF
39DAF
42DAF
T.S
K
F

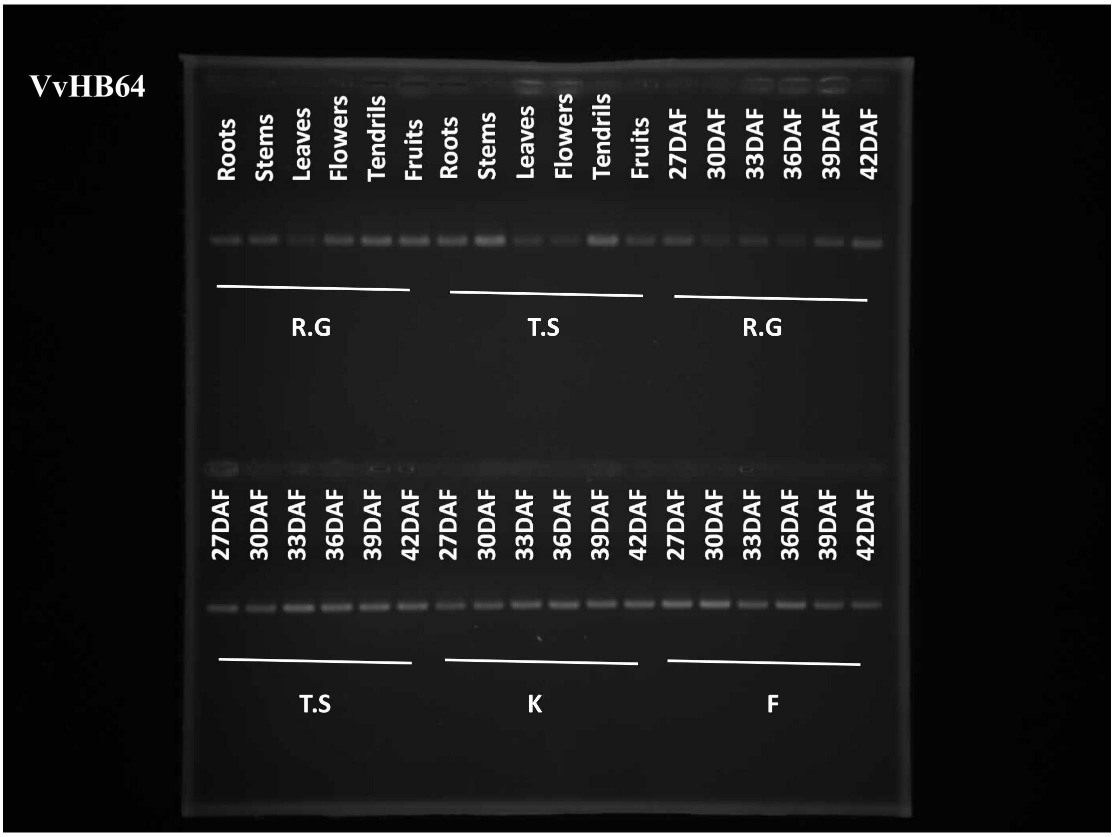
VvHB64
Roots
Stems
Leaves
Flowers
Tendrils
Fruits
Roots
Stems
Leaves
Flowers
Tendrils
Fruits
27DAF
30DAF
33DAF
36DAF
39DAF
42DAF
R.G
T.S
R.G
27DAF
30DAF
33DAF
36DAF
39DAF
42DAF
27DAF
30DAF
33DAF
36DAF
39DAF
42DAF
27DAF
30DAF
33DAF
36DAF
39DAF
42DAF
T.S
K
F

Supplementary Figure S6: Relative expression levels of *VvHB62, VvHB56* and *VvHB63* three genes in sarcocarp, fruits and ovules. Sarcocarp which was separated from fruits (at 42 days post anthesis) and ovules (42 days after full bloom) of 'Thompson Seedless' and 'Red Globle' were used as samples.

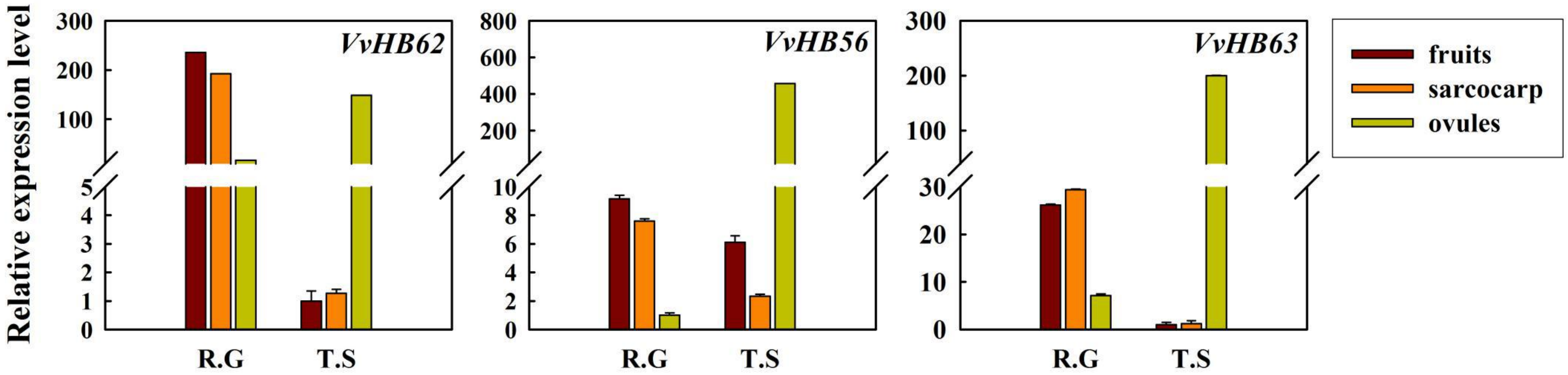

Supplementary Table S1: Number of homeobox genes subfamily members identified in *Arabidopsis* and *Vitis vinifera*. A comparison of subfamily representation between grape and *Arabidopsis* is given as ratio.

**Subfamily**	***A.thaliana***	***V.vinifera***	**Ratio**
HD-ZIP I	17	13	0.76
HD-ZIP II	10	7	0.7
HD-ZIP III	5	5	1
HD-ZIP IV	16	8	0.5
PLINC	14	0	0
WOX	16	11	0.69
KNOX	8	9	1.13
BEL	13	12	0.92
DDT	4	4	1
PHD	2	2	1
NDX	1	0	0
LD	1	0	0
PINTOX	1	1	1
SAWADEE	2	1	0.5
Total	110	73	0.66

Supplementary Table S2: Conserved motifs of grape and *Arabidopsis* HB genes. (P value < 0.05)

**No.**	**Sequence of the motif**	**P value**	**No. of VvHB/ATHB proteins in which motif is present**	
			**VvHB proteins**	**ATHB proteins**
1	LEPKQVKNWFQNRRAREKKKQEE	2.9E-1261	72	109
2	YHRHTPEQIZILERLFKECPH	3.1E-461	51	69
3	ARRLPSGCLIQDCPNGYSKVTWVEHVEVE	3.9E-464	13	21
4	PERAVSVLRAWLFEHFLHPYPKESDKIML	4.4E-440	21	21
5	GGTLZLMYAZLQVPSPLVPTREFYFLRYCKQ	5.9E-411	13	21
6	GEPPGIVLSAATSFWLPVPPQRVFDFLRDERTRSZWDILSNGNPVQEMAH	3.9E-461	8	16
7	IAEETLAEFLSKATGTAVDWVQMPGMKPGPDSIGIVAISHGCSGVAARAC	1.7E-341	5	5
8	RYKQYYHQMQIVVSSFEQVAGLGAAKPYTALALQTISRHFRCLKDAISGQ	1.3E-362	10	12
9	HELYRPLVNSGLAFGAKRWVATLQRQCER	4.6E-284	8	16
10	GVLCAKASMLLQNVPPAILVRFLREHRSEWADYSVDAYSAA	7.1E-256	5	5
11	DGGDPDYIPJLPSGFRIIPDG	6.5E-255	13	21
12	EASRESGVVIMNAINLVEIFMDVNKWSELFPSIVSRAKTLEVJ	1.9e-346	8	16
13	SRLQTVNRKLTAMNKLLMEENDRLQKQVSQLVYENGYMRQQ	1.9E-249	5	5
14	KALCSEFPKIMQQGFACLPAGICLSSMGRPVSYEQAVAWKV	6.7E-231	5	5
15	PVFTFANQAGLDMLETTLVALQDITLDKI	5.3E-182	5	5
16	VYGWGRQPAVLRAFSQRLSRGFNDAVNGFTDDGW	9.8E-181	5	5
17	QSLRIENARLKEELDRLSAJA	7.1E-169	28	43
18	ESHPTAKLALMSVQTVNNLISSTVRVIKA	7.9E-209	12	18
19	HENVALRAENDKLRAENIALREALKNAJCPNCGGPAVIGEM	1.3E-273	8	14
20	AWSVPEVLRPLYESSKVLAQKTTVAALRHJRQIAQETSGEV	2.4E-205	5	5

No.	Subfamilies in which motif is present in Grape/Arabidopsis	
	**in Grape**	**in Arabidopsis**
1	HD-ZIP I-IV, WOX, SAWADEE, PHD, DDT, KNOX, BEL	HD-ZIP I-IV, WOX, SAWADEE, PHD, DDT, KNOX, BEL, NDX, LD, PLINC
2	HD-ZIP I-IV, WOX, SAWADEE, PHD, DDT, PINTOX	HD-ZIP I-IV, WOX, DDT, PINTOX,
3	HD-ZIP III, HD-ZIP IV	HD-ZIP III, HD-ZIP IV
4	KNOX, BEL	KNOX, BEL
5	HD-ZIP III, HD-ZIP IV	HD-ZIP III, HD-ZIP IV
6	HD-ZIP IV	HD-ZIP IV
7	HD-ZIP III	HD-ZIP III
8	BEL	BEL
9	HD-ZIP IV	HD-ZIP IV
10	HD-ZIP III	HD-ZIP III
11	HD-ZIP III, HD-ZIP IV	HD-ZIP III, HD-ZIP IV
12	HD-ZIP IV	HD-ZIP IV
13	HD-ZIP III	HD-ZIP III
14	HD-ZIP III	HD-ZIP III
15	HD-ZIP III	HD-ZIP III
16	HD-ZIP III	HD-ZIP III
17	HD-ZIP I, HD-ZIP II, HD-ZIP IV	HD-ZIP I, HD-ZIP II, HD-ZIP IV
18	HD-ZIP III, HD-ZIP IV	HD-ZIP III, HD-ZIP IV
19	HD-ZIP IV	HD-ZIP IV
20	HD-ZIP III	HD-ZIP III

Supplementary Table S3: Tandem duplication events in the 73 *VvHB* genes

**Cluster number**	**Chromosome**	**Gene**	**Start Site**	**End Site**
1	chr1	VvHB01 (HD-ZIP II)<br>VvHB02 (WOX)	4488301<br>4685930	4491290<br>4687708
2	chr4	VvHB20 (WOX)<br>VvHB21 (WOX)	20843708<br>20861325	20847365<br>20865130
3	chr10	VvHB36 (DDT)<br>VvHB37 (WOX)	1562230<br>1805314	1582041<br>1808227
4	chr18	VvHB63 (KNOX)<br>VvHB64 (HD-ZIP II)	6870320<br>6881993	6876771<br>6883411
5	chr18	VvHB67 (BEL)<br>VvHB68 (BEL)	11196526<br>11201410	11198339<br>11205044
6	chr19	VvHB69 (SAWADEE)<br>VvHB70 (HD-ZIP I)	2065687<br>2259485	2074580<br>2272052

Supplementary Table S4: Synteny blocks of *VvHB* genes within grape genomes

ID*	region1(*Grape*)			region2(*Grape*)			Gene in the synteny region		Gene name	
	Chr	Start	Stop	Chr	Start	Stop	Gene 1	Gene 2	Gene 1	Gene 2
30	chr13	23812858	24096128	chr8	20724328	19994166	GSVIVG01001366001	GSVIVG01033481001	VvHB48 (HD-ZIP II)	VvHB32 (HD-ZIP II)
49	chr17	8454312	10330607	chr1	10011362	7333255	GSVIVG01007715001	GSVIVG01013790001	VvHB59 (KNOX)	VvHB3 (KNOX)
45	chr17	5814755	7784744	chr1	10067645	15346615	GSVIVG01008065001	GSVIVG01020078001	VvHB58 (HD-ZIP I)	VvHB4 (HD-ZIP I)
48	chr17	1666197	4710077	chr1	5375923	2251732	GSVIVG01008424001	GSVIVG01011738001	VvHB57 (WOX)	VvHB2 (WOX)
67	chr18	10068356	12810091	chr4	17429918	20390368	GSVIVG01009633001	GSVIVG01019043001	VvHB66 (BEL)	VvHB17 (BEL)
58	chr18	11196526	12904974	chr3	40975	1730564	GSVIVG01009779001	GSVIVG01024224001	VvHB67 (BEL)	VvHB10 (BEL)
41	chr16	13450982	16162952	chr15	14506625	16138896	GSVIVG01010600001	GSVIVG01027508001	VvHB55 (HD-ZIP IV)	VvHB53 (HD-ZIP IV)
32	chr14	28844329	29813645	chr1	10815152	13910631	GSVIVG01011377001	GSVIVG01020033001	VvHB51 (HD-ZIP I)	VvHB5 (HD-ZIP I)
13	chr11	5869916	7689143	chr4	6447365	5372703	GSVIVG01012897001	GSVIVT01035921001	VvHB40 (KNOX)	VvHB15 (KNOX)
25	chr13	4546379	6087112	chr6	4760340	2948720	GSVIVT01016272001	GSVIVT01025193001	VvHB47 (HD-ZIP III)	VvHB23 (HD-ZIP III)
126	chr9	3404650	7445837	chr4	2669216	5171733	GSVIVT01017010001	GSVIVT01035612001	VvHB33 (HD-ZIP III)	VvHB13 (HD-ZIP III)
81	chr2	236398	2725052	chr15	19083025	16064252	GSVIVT01019655001	GSVIVT01027407001	VvHB7 (HD-ZIP I)	VvHB54 (HD-ZIP I)
86	chr2	1560083	2576046	chr16	21974662	21111941	GSVIVT01019655001	GSVIVT01038619001	VvHB7 (HD-ZIP I)	VvHB56 (HD-ZIP I)
18	chr12	1992495	4110237	chr10	3493620	1346397	GSVIVT01020605001	GSVIVT01021113001	VvHB42 (DDT)	VvHB36 (DDT)
38	chr15	16447701	17409766	chr16	21102338	21974662	GSVIVT01027407001	GSVIVT01038619001	VvHB54 (HD-ZIP I)	VvHB56 (HD-ZIP I)

* ID means the synteny block ID in the synteny analysis (Nucleic Acids Res, 2012. 40(7): e49.)

Supplementary Table S5: Synteny blocks of homeobox genes between grape and *Arabidopsis*

ID*	region1 (Arabidopsis)			region2 (Grape)			Gene in the synteny region	
	Chr	Start	Stop	Chr	Start	Stop	Gene 1	Gene 2
53	Chr1	1502344	1542432	chr12	7051966	7470668	AT1G05230 (HD-ZIP IV)	VvHB44 (HD-ZIP IV)
122	Chr1	6998489	7382462	chr18	1780353	70305	AT1G20700 (WOX)	VvHB61 (WOX)
105	Chr1	8260865	8569996	chr17	10814954	8528864	AT1G23380 (KNOX)	VvHB59 (KNOX)
0	Chr1	8295185	8404301	chr1	7494007	8210725	AT1G23380 (KNOX)	VvHB3 (KNOX)
76	Chr1	9182204	9465444	chr14	30137019	28545588	AT1G26960 (HD-ZIP I)	VvHB51 (HD-ZIP I)
32	Chr1	9295736	9361833	chr1	11687175	11234424	AT1G26960 (HD-ZIP I)	VvHB5 (HD-ZIP I)
60	Chr1	9931934	10231142	chr12	4105274	2352608	AT1G28420 (DDT)	VvHB42 (DDT)
40	Chr1	9911898	10266818	chr10	1335630	3199699	AT1G28420 (DDT)	VvHB36 (DDT)
52	Chr1	22932901	23104647	chr12	5491822	6546456	AT1G62360 (KNOX)	VvHB43 (KNOX)
160	Chr1	23331676	23469204	chr2	4250807	3480175	AT1G62990 (KNOX)	VvHB8 (KNOX)
74	Chr1	26063816	26626503	chr14	30011357	27051487	AT1G69780 (HD-ZIP I)	VvHB51 (HD-ZIP I)
92	Chr1	26363278	26621659	chr17	8454312	10707300	AT1G70510 (KNOX)	VvHB59 (KNOX)
30	Chr1	26561824	26600219	chr1	7875921	7333255	AT1G70510 (KNOX)	VvHB3 (KNOX)
16	Chr1	26735956	26828674	chr1	4491290	4016641	AT1G70920 (HD-ZIP II)	VvHB1 (HD-ZIP II)
206	Chr2	163954	237147	chr1	4359602	4748971	AT2G01430 (HD-ZIP II)	VvHB1 (HD-ZIP II)
259	Chr2	88847	243493	chr17	2869660	1966513	AT2G01500 (WOX)	VvHB57 (WOX)
284	Chr2	7634274	7990586	chr4	20841170	18395062	AT2G17950 (WOX)	VvHB19 (WOX)
307	Chr2	9504659	9975192	chr7	14791101	16682195	AT2G22430 (HD-ZIP I)	VvHB27 (HD-ZIP I)
260	Chr2	9513219	9946269	chr18	5092140	8531571	AT2G22800 (HD-ZIP II)	VvHB64 (HD-ZIP II)
262	Chr2	9904787	10132779	chr18	9477475	10230335	AT2G23760 (BEL)	VvHB66 (BEL)
283	Chr2	10097559	10132779	chr4	17398366	17614321	AT2G23760 (BEL)	VvHB17 (BEL)
297	Chr2	11921433	13011063	chr6	3098092	9133237	AT2G27990 (BEL)	VvHB22 (BEL)
236	Chr2	13713036	13747791	chr12	7470668	7078502	AT2G32370 (HD-ZIP IV)	VvHB44 (HD-ZIP IV)

ID*	region1(Arabidopsis)			region2(Grape)			Gene in the synteny region	
	Chr	Start	Stop	Chr	Start	Stop	Gene 1	Gene 2
214	Chr2	14300772	14515915	chr10	1304688	3273334	AT2G33880 (WOX)	VvHB37 (WOX)
317	Chr2	15106940	15511514	chr8	16424080	18999734	AT2G36610 (HD-ZIP I)	VvHB31 (HD-ZIP I)
252	Chr2	18379564	18853709	chr15	9935686	16207483	AT2G44910 (HD-ZIP II)	VvHB52 (HD-ZIP II)
271	Chr2	19153328	19262404	chr2	2231721	2551460	AT2G46680 (HD-ZIP I)	VvHB7 (HD-ZIP I)
253	Chr2	18855775	19277773	chr15	20079735	16362838	AT2G46680 (HD-ZIP I)	VvHB54 (HD-ZIP I)
256	Chr2	19086561	19235087	chr16	21974662	21228111	AT2G46680 (HD-ZIP I)	VvHB56 (HD-ZIP I)
375	Chr3	1653	350010	chr14	30170199	25221541	AT3G01220 (HD-ZIP I)	VvHB51 (HD-ZIP I)
361	Chr3	3521357	3597952	chr13	5231188	3298345	AT3G11260 (WOX)	VvHB46 (WOX)
394	Chr3	6094098	6403348	chr17	1518274	4537653	AT3G18010 (WOX)	VvHB57 (WOX)
385	Chr3	22222105	22625716	chr15	9984817	15448056	AT3G60390 (HD-ZIP II)	VvHB52 (HD-ZIP II)
386	Chr3	22585655	22658390	chr15	15541545	16213665	AT3G61150 (HD-ZIP IV)	VvHB53 (HD-ZIP IV)
387	Chr3	22662960	22958995	chr15	20114200	16407196	AT3G61890 (HD-ZIP I)	VvHB54 (HD-ZIP I)
536	Chr4	9354321	9604207	chr15	13756927	10988829	AT4G16780 (HD-ZIP II)	VvHB52 (HD-ZIP II)
538	Chr4	9714092	9833006	chr15	13469630	10890572	AT4G17460 (HD-ZIP II)	VvHB52 (HD-ZIP II)
503	Chr4	11548618	11776039	chr10	260075	1213743	AT4G21750 (HD-ZIP IV)	VvHB35 (HD-ZIP IV)
540	Chr4	12901534	13108827	chr16	9082240	18062107	AT4G25530 (HD-ZIP IV)	VvHB55 (HD-ZIP IV)
517	Chr4	14601621	14737978	chr11	18015419	15969015	AT4G29940 (PHD)	VvHB41 (PHD)
519	Chr4	15419404	15525448	chr11	7689143	5932036	AT4G32040 (KNOX)	VvHB40 (KNOX)
588	Chr4	15383201	15500847	chr4	5247592	5930252	AT4G32040 (KNOX)	VvHB15 (KNOX)
589	Chr4	15831576	15926628	chr4	480827	846927	AT4G32980 (BEL)	VvHB12 (BEL)
594	Chr4	16865488	17279201	chr4	20881776	18296443	AT4G35550 (WOX)	VvHB20 (WOX)
597	Chr4	17298200	17373930	chr4	17886811	17429918	AT4G36740 (HD-ZIP I)	VvHB18 (HD-ZIP I)
552	Chr4	17093088	17541799	chr18	11980862	9477475	AT4G36870 (BEL)	VvHB66 (BEL)
554	Chr4	17540490	17874804	chr18	8237760	5846804	AT4G37790 (HD-ZIP II)	VvHB64 (HD-ZIP II)

ID*	region1(Arabidopsis)			region2(Grape)			Gene in the synteny region	
	**Chr**	**Start**	**Stop**	**Chr**	**Start**	**Stop**	**Gene 1**	**Gene 2**
549	Chr4	18384100	18584524	chr18	3963411	5336005	AT4G40060 (HD-ZIP I)	VvHB62 (HD-ZIP I)
790	Chr5	383153	659733	chr8	15471385	14139513	AT5G02030 (BEL)	VvHB30 (BEL)
788	Chr5	908563	1015345	chr8	17614517	18269207	AT5G03790 (HD-ZIP I)	VvHB31 (HD-ZIP I)
657	Chr5	1734487	1836722	chr13	4547834	3298345	AT5G05770 (WOX)	VvHB46 (WOX)
792	Chr5	2029897	2129324	chr8	20735141	19953117	AT5G06710 (HD-ZIP II)	VvHB32 (HD-ZIP II)
735	Chr5	3459273	3519449	chr4	5372703	6059248	AT5G11060 (KNOX)	VvHB15 (KNOX)
740	Chr5	3459273	3747125	chr4	5390746	2657661	AT5G11270 (PINTOX)	VvHB14 (PINTOX)
663	Chr5	4477354	5057411	chr14	24525304	30253071	AT5G15150 (HD-ZIP I)	VvHB51 (HD-ZIP I)
644	Chr5	17763681	17843751	chr12	3753289	4425457	AT5G44180 (DDT)	VvHB42 (DDT)
626	Chr5	18507489	18700656	chr10	3919935	1588546	AT5G45980 (WOX)	VvHB37 (WOX)
681	Chr5	19090298	19217909	chr15	10798618	12593884	AT5G47370 (HD-ZIP II)	VvHB52 (HD-ZIP II)
685	Chr5	20797017	21643562	chr16	8000259	19401729	AT5G52170 (HD-ZIP IV)	VvHB55 (HD-ZIP IV)
683	Chr5	20913553	21199959	chr15	14614856	16138896	AT5G52170 (HD-ZIP IV)	VvHB53 (HD-ZIP IV)
724	Chr5	21725441	21948913	chr19	3310746	1981264	AT5G53980 (HD-ZIP I)	VvHB70 (HD-ZIP I)
772	Chr5	26083328	26275919	chr7	14787888	15197600	AT5G65310 (HD-ZIP I)	VvHB27 (HD-ZIP I)
715	Chr5	25977621	26365998	chr18	4121977	7292423	AT5G65310 (HD-ZIP I)	VvHB62 (HD-ZIP I)
736	Chr5	26584780	26644402	chr4	17449433	17936828	AT5G66700 (HD-ZIP I)	VvHB18 (HD-ZIP I)

* ID refers to the synteny block ID in the synteny analysis (Nucleic Acids Res, 2012. 40(7): e49.)

Supplementary Table S6: Transcriptome analysis result of 33 grape HB genes at three ovules/seeds developmental stages. "Ratio" refers to the ratio of the gene expression levels in the seedless grape varieties to the gene expression levels in the seeded grape varieties. P-value less than 0.05 indicates significantly changed pathways and all the genes are significant differentially expressed genes at least at one developmental stage.

		**Stages of ovule development**					
**Gene ID**	**Gene locus ID**	**Stage 1**		**Stage 2**		**Stage 3**	
		**Ratio**	**P-value**	**Ratio**	**P-value**	**Ratio**	**P-value**
VvHB27	GSVIVT01003431001	4.996838111	6.03E-09	4.39826087	4.55E-68	5.336307481	4.17E-11
VvHB39	GSVIVT01004811001	268	1.90E-08	185	3.23E-09	104	1.78E-07
VvHB59	GSVIVT01007715001	70.75	3.88E-05	34.25	0.000729715	277	4.07E-05
VvHB58	GSVIVT01008065001	9.471451021	1.12E-19	10.69092332	1.20E-120	6.157422081	3.43E-12
VvHB62	GSVIVT01009083001	221.25	1.33E-15	106.8888889	9.69E-29	14.25	0.01441004
VvHB63	GSVIVT01009273001	28.66666667	2.22E-12	32.64285714	6.47E-18	25.38235294	5.16E-13
VvHB64	GSVIVT01009274001	31.26923077	3.36E-08	7.95	4.54E-06	1.07480916	0.634675366
VvHB55	GSVIVT01010600001	0.400440044	3.12E-08	0.326139089	1.98E-30	3.207133059	0.001607896
VvHB40	GSVIVT01012897001	2.561125445	0.021634725	2.493445279	6.13E-25	5.813516098	3.75E-12
VvHB61	GSVIVT01013388001	4.964356436	9.18E-06	2.607907743	2.92E-11	2.888268156	0.013952167
VvHB3	GSVIVT01013790001	107.5	4.54E-11	395	4.43E-09	125.75	1.18E-07
VvHB52	GSVIVT01018247001	3.564453125	0.004619071	2.63285834	6.78E-10	4.893867925	5.02E-05
VvHB18	GSVIVT01019012001	257.2365591	1.51E-72	4.00109529	3.09E-27	89.83076923	3.79E-48
VvHB17	GSVIVT01019043001	0.234348076	2.11E-05	0.236607143	1.76E-53	0.877865961	0.111375672
VvHB6	GSVIVT01019399001	7.384444444	1.14E-14	6.815384615	2.77E-66	7.859916782	0.000134842
VvHB7	GSVIVT01019655001	79.48484848	1.73E-34	1.971875	0.000108701	75.70689655	7.02E-16
VvHB5	GSVIVT01020033001	4.349232013	2.74E-06	6.60952381	1.24E-67	6.493975904	2.55E-09
VvHB37	GSVIVT01021144001	53.42857143	2.06E-18	59.72727273	1.24E-21	6.019230769	0.013637546
VvHB38	GSVIVT01021625001	4.19760479	1.36E-05	0.171428571	5.19E-62	0.862393968	0.034097501
VvHB23	GSVIVT01025193001	4.164133739	0.000637408	2.213333333	8.14E-06	3.983122363	0.000234732

Gene ID	Gene locus ID	Stages of ovule development					
		Stage 1		Stage 2		Stage 3	
		Ratio	P-value	Ratio	P-value	Ratio	P-value
VvHB21	GSVIVT01026636001	4.815724816	2.11E-07	3.422758621	5.58E-25	5.592682927	5.05E-09
VvHB20	GSVIVT01026638001	5.581679389	6.85E-08	5.438247012	1.74E-26	10.91623037	1.64E-11
VvHB54	GSVIVT01027407001	30.39130435	1.48E-21	9.941176471	4.32E-16	9.383900929	6.22E-13
VvHB53	GSVIVT01027508001	8.577319588	1.01E-15	6.553846154	2.09E-45	4.099156118	0.014826987
VvHB60	GSVIVT01029396001	3.214285714	0.004427312	2.898039216	9.15E-05	1.662337662	0.951488647
VvHB44	GSVIVT01030605001	3.50502008	0.000115857	2.040256176	1.10E-08	2.354679803	0.147958527
VvHB49	GSVIVT01031241001	17.05555556	2.02E-19	8.583333333	1.09E-30	15.01382488	2.94E-19
VvHB50	GSVIVT01032491001	4.13152094	3.71E-06	3.241618497	6.09E-26	4.300859599	3.85E-07
VvHB32	GSVIVT01033481001	10.70779221	6.99E-10	7.623188406	5.82E-19	28.23636364	0.000120755
VvHB16	GSVIVT01035238001	0.111111111	0.008867926	0.057220708	3.45E-17	0.658653846	0.08332567
VvHB15	GSVIVT01035921001	8.721774194	1.22E-17	5.461113899	1.91E-74	8.91973792	1.32E-18
VvHB25	GSVIVT01037575001	17.57030016	4.30E-31	16.32782369	1.72E-178	18.31573276	1.01E-30
VvHB56	GSVIVT01038619001	376.8333333	5.20E-29	226.1	3.07E-57	185	4.34E-38

Supplementary Table S7: Primer sequences used in expression analysis of the homeobox genes in grape

Gene	Gene ID (Accession No.)		Forward and reverse primer sequence (5'~3')
VvHB27	GSVIVT01003431001<br>XM_003632428.2	F<br>R	AAGTAGCCGTTTGGTTCC<br>GCTTCATTGTCGTGTTGG
VvHB39	GSVIVT01004811001<br>XM_002271908.2	F<br>R	CTACCTAAAGAAGCCCG<br>GCATCCATAACCACAAAC
VvHB59	GSVIVT01007715001<br>XM_002282040.2	F<br>R	TCCGATTACCACCACCCGC<br>GACCACCAAAGACGACGCC
VvHB58	GSVIVT01008065001<br>XM_002271656.3	F<br>R	GAGAGGGGAAACTTGGAGGTGT<br>TTTGTGGGAGGGTCGGGGTAAT
VvHB62	GSVIVT01009083001<br>XM_002285707.2	F<br>R	GCAGGGTAAGAACAATGGAAAT<br>CCAAACTGATGCTGAAACGC
VvHB63	GSVIVT01009273001<br>XM_002285485.3	F<br>R	GCCATACCCCTCAGAGACAG<br>GCCCCAAACGATAAGCACC
VvHB64	GSVIVT01009274001<br>XM_002283511.2	F<br>R	GCTTCAAACAACACAGCACTCT<br>GCAACGCTTTCTCAACAACTC
VvHB55	GSVIVT01010600001<br>XM_010664205.1	F<br>R	TGGGGAAGGATTATGGGCTG<br>CGGTAGAGATAGTGGACGGC
VvHB40	GSVIVT01012897001<br>XM_010658241.1	F<br>R	AGTTGCTTTCCGCTCACG<br>GCCATTACGGCTTCCATAG
VvHB61	GSVIVT01013388001<br>XM_002272384.2	F<br>R	GTTGTTGGAGGAGGGAGTG<br>CTTGTGACCAGCGGATGC
VvHB3	GSVIVT01013790001<br>XM_002263277.2	F<br>R	GCTCTTACGGAGGTTTGGTG<br>CAGTGCCGCTTTCGTTGAT
VvHB52	GSVIVT01018247001<br>XM_010663078.1	F<br>R	TTGAAGCAGACGGAGGTGGAC<br>GAGGCGGGTGATGGAGAG
VvHB18	GSVIVT01019012001<br>XM_002272971.2	F<br>R	GCCGAGAAGGAGATACGACG<br>CCCCATTCCATGCCACTAAC
VvHB17	GSVIVT01019043001<br>XM_002269634.2	F<br>R	CTTTCCTTGTCCCTATCCTCT<br>CTCATAACATTCACCGCT
VvHB6	GSVIVT01019399001<br>XM_002279194.3	F<br>R	GGTTCATCAATGCGAGGGTG<br>TTGTCGTCGGGGTCTGTAG
VvHB7	GSVIVT01019655001<br>XM_002280012.2	F<br>R	CCCTCGTCAGGTTGCCAT<br>GAATTAGTGTACCCGATCTCCG
VvHB5	GSVIVT01020033001<br>XM_002276853.2	F<br>R	GCCTCCTAACTCTCTCAATCC<br>TCATCAGACAAATCATCCTCC

Gene	Gene ID (Accession No.)		Forward and reverse primer sequence (5'~3')
VvHB37	GSVIVT01021144001<br>XM_002273152.2	F<br>R	ATCAACCCATCTCTCATTTC<br>CTTCCTTATCTCATCCCTCG
VvHB38	GSVIVT01021625001<br>XM_002281832.3	F<br>R	CAGCAAAACCCAACACCT<br>GGCAGTCACGAAACCAAG
VvHB23	GSVIVT01025193001<br>XM_010652862.1	F<br>R	TGGCTCGTCAGTATGTTCGG<br>AAGTCCTGCCTGGTTTGC
VvHB21	GSVIVT01026636001<br>XM_002272827.2	F<br>R	GCAGGAGTCAGACTGGGAAATC<br>GCTGGCTCAAATCAGAGGTTATC
VvHB20	GSVIVT01026638001<br>XM_002279906.2	F<br>R	GAAGGTGATGACAGACGC<br>TCAAAAATACGCTCAAGAAT
VvHB54	GSVIVT01027407001<br>XM_002262914.3	F<br>R	GTGAAGCAGACAACAGAGAC<br>TCAAGCAACCATCAACAGG
VvHB53	GSVIVT01027508001<br>XM_010663259.1	F<br>R	CTTTCACCTTTGGTTCCTGT<br>GCACTCTCATCGTATTCT
VvHB60	GSVIVT01029396001<br>XM_002270976.2	F<br>R	AGACAAAACCCCAACTCATCG<br>ACAAGCAAATCTCTCGCACAT
VvHB44	GSVIVT01030605001<br>XM_010659009.1	F<br>R	GCGAATGGTCAAGACACAGG<br>GACAGGAGCATAAATCACGAAAG
VvHB49	GSVIVT01031241001<br>XM_002277895.2	F<br>R	CAGTGGAGAACAGTAACG<br>TCAATGTAGGCGGAGAC
VvHB50	GSVIVT01032491001<br>XM_002278836.3	F<br>R	GTGACTCCTACTTCCAAAATGC<br>CAACAAAGCCTCTCCCTCC
VvHB32	GSVIVT01033481001<br>XM_002275711.2	F<br>R	GACTGGAATTATTGGGAAGGGAC<br>CGAAGAAGGATGATGGCGAG
VvHB16	GSVIVT01035238001<br>XM_002268236.2	F<br>R	TCCCACAATCGTTACCAAAGC<br>TAGGACACATCCACCATCACC
VvHB15	GSVIVT01035921001<br>XM_010650411.1	F<br>R	GATTTGGTCCTCTTGTCCCT<br>CAACTTTGCCTTGTCCTCCTC
VvHB25	GSVIVT01037575001<br>XM_002275062.3	F<br>R	CCTGAGTGGAGGCGAAAG<br>AGCCAAGGCAGTGTATGATT
VvHB56	GSVIVT01038619001<br>XM_002271487.2	F<br>R	CAGTATCTGGAGTCTATTTTCG<br>GCTCTGAGTGCTTTGTAGTC
Actin1	NC_012010	F<br>R	GATTCTGGTGATGGTGTGAGT<br>GACAATTTCCCGTTCAGCAGT
EF1-α	NC_012012	F<br>R	AGGAGGCAGCCAACTTCACC<br>CAAACCCTGCATCACCATTC
